# Quantifying quality in DNA self-assembly

**DOI:** 10.1038/ncomms4691

**Published:** 2014-04-22

**Authors:** Klaus F. Wagenbauer, Christian H. Wachauf, Hendrik Dietz

**Affiliations:** 1 Physik Department, Walter Schottky Institute, Technische Universität München, Am Coulombwall 4a, 85748 Garching, Germany; 2 These authors contributed equally to this work

## Abstract

Molecular self-assembly with DNA is an attractive route for building nanoscale devices. The development of sophisticated and precise objects with this technique requires detailed experimental feedback on the structure and composition of assembled objects. Here we report a sensitive assay for the quality of assembly. The method relies on measuring the content of unpaired DNA bases in self-assembled DNA objects using a fluorescent de-Bruijn probe for three-base ‘codons’, which enables a comparison with the designed content of unpaired DNA. We use the assay to measure the quality of assembly of several multilayer DNA origami objects and illustrate the use of the assay for the rational refinement of assembly protocols. Our data suggests that large and complex objects like multilayer DNA origami can be made with high strand integration quality up to 99%. Beyond DNA nanotechnology, we speculate that the ability to discriminate unpaired from paired nucleic acids in the same macromolecule may also be useful for analysing cellular nucleic acids.

## Abstract (web summary)

Sequence-programmable self-assembly of DNA enables the formation of a variety of complex structures; however, determining the quality of these multi-chain structures is challenging. Here the authors address this problem by using a fluorescent probe to measure the amount of unpaired bases in the DNA assemblies.

## Graphical abstract

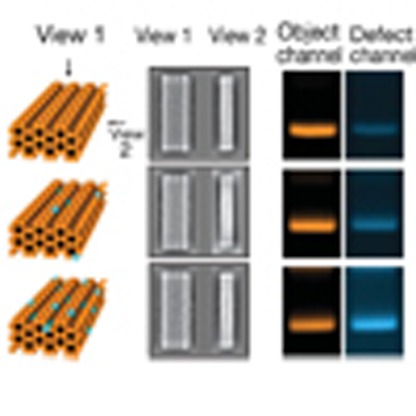

A key goal of nanotechnology is the construction of nanoscale devices and machines that can accomplish custom tasks. Molecular self-assembly with DNA is a candidate route towards building such objects^1^, because it offers the possibility to construct user-defined, chemically registered and structurally complex objects with absolute dimensions on the tens to hundreds of nanometres scale. Several DNA nanodevices highlight the potential for utility of a DNA-based approach to nanotechnology^2^,^3^,^4^,^5^,^6^,^7^,^8^,^9^,^10^,^11^. Designed DNA crystals^12^ and high-resolution cryo-electron microscopy (EM) structures of discrete DNA objects^13^,^14^ strengthen a perspective in which target objects may even be specified with near atomic-level positional accuracy. Advanced functionalities such as molecular recognition or enzyme-like catalysis might thus be achievable with ultra-precise DNA positioning scaffolds. However, making more sophisticated and more precise DNA objects will depend not only on advanced methods of design, but also on the ability to synthesize objects with the highest possible quality. Achieving both requires detailed experimental feedback. But as it has also been pointed out elsewhere^15^, evaluating the quality of assembly with sufficient detail has so far been challenging.

Here we report a solution to this problem in the form of a sensitive assay for the content of unpaired DNA bases in self-assembled DNA objects. Why is the content of unpaired DNA bases a good measure for the quality of assembly? First, double-helical DNA (dsDNA) domains represent the fundamental building blocks in DNA nanotechnology and thus typically constitute the bulk of designed objects^16^,^17^. Unpaired DNA elements, by contrast, are used much more rarely by designers in contexts that exploit their flexibility, that is, as linkers and hinges^18^, as entropic passivation against blunt-ended dsDNA domain contacts^17^,^19^,^20^ and as a transducer of tension^21^. Second, all current DNA nanostructure design approaches rely on the simple principle that the target structure should be the one that minimizes the number of unpaired DNA bases. A remainder of unpaired DNA bases in assembled objects that exceeds the one specified by the designer hence indicates incomplete self-assembly.

## Results

### Detecting unpaired DNA

**Figure 1:**
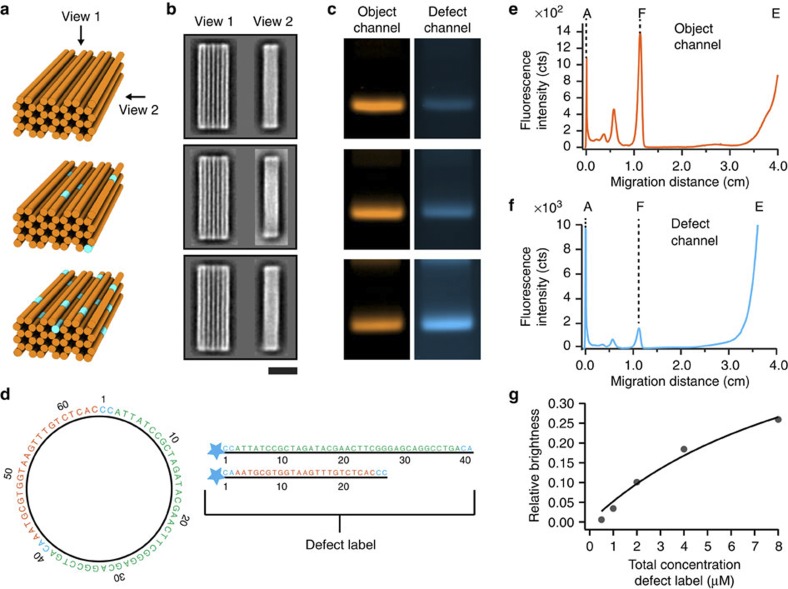
Motivation and proof-of-concept of a defect assay for DNA nanotechnology. (**a**) Schematic representation of 42-helix multi-layer DNA origami objects. Orange cylinders indicate double-helical DNA domains and segments drawn in cyan indicate unpaired DNA elements (structural defects). (**b**) Averaged negative-stain TEM images of self-assembled 42-helix-bundle variants. Top: all 207 required DNA staple strands included in the reaction mixture, middle and bottom panel: 5 and 15 staple strands were omitted from the mixtures. The strands were picked at random. See Supplementary Figs 1–3 for design details. Scale bar, 20 nm. (**c**) Dual-channel laser-scanned fluorescence images (false coloured) of agarose gels on which the 42-helix-bundle variants were electrophoresed. (**d**) Left: a circular 64-bases de-Bruijn sequence of order 3 for the alphabets A, T, G, C. Right: the two oligonucleotide sequences that were derived from the de-Bruijn sequence. The star indicates a cyanine 3 modification at the 5′ terminus. (**e**,**f**) Gel-electrophoretic lane intensity profiles in the object and defect channel, respectively. A indicates the gel pocket, F indicates the ‘folded’ target object band and E indicates the band produced by excess object and defect labels. (**g**) Relative defect label brightness as a function of the total defect label concentration. Circles: experimental data. Solid line: fit with a bimolecular reaction model.

How can one detect the existence of unpaired DNA ‘defects’ in DNA nanostructures? Routine structural feedback in DNA nanotechnology is obtained at multiple-nanometre resolution by direct imaging of single particles by atomic force microscopy or transmission EM (TEM). Direct imaging of single particles by atomic force microscopy may be used to evaluate the assembly quality of flat, two-dimensional (2D) DNA objects by counting obvious holes and other shape deformations^17^,^22^. However, to illustrate the difficulty of experimentally resolving defects at multiple-nanometre resolution in objects that extend in all 3D, we designed a 42-helix multilayer DNA origami object^20^,^23^ and prepared variants of it in which subsets (up to 7%) of the required DNA staple strands (207 DNA ‘staple’ oligonucleotides’ total, 1 DNA scaffold strand) were omitted from the self-assembly reaction mixtures, thus creating systematic unpaired DNA pseudo-defects (Fig. 1a, see Supplementary Figs 1–3 for design details). We collected negative-staining TEM micrographs from each variant and computed average micrographs from two dominant projection views (Fig. 1b, see Supplementary Figs 4–9 for single particle TEM micrographs)^24^. At this level of analysis, no difference could be discerned between the pseudo-defective variants and the putative non-defective variant, that is, the one produced in the reaction mixture containing all required strands. All variants might be judged as high-quality assembly products. But a background of up to 7% defects constitutes a significant and undesirable deviation from designed specifications. A more sophisticated analysis of cryo-EM data might be able to resolve site-specific defects, but it will be blind to stochastically distributed defects.

Our method for detecting unpaired DNA elements consists in (A) adding a fluorescent object label, (B) incubating the self-assembly reaction products with a fluorescent label for unpaired DNA (detailed below), (C) separating the double-labelled reaction products from excess object and defect labels via gel electrophoresis and (D) recording the fluorescence intensity of the target object in the emission channels of the object and defect label, respectively. The working principle of the assay is illustrated exemplarily with the three variants of the 42-helix bundle discussed above (Fig. 1c). The specific object labelling was achieved by replacing one of the DNA staple strands with a cyanine-5-modified version in each of the three self-assembly reaction mixtures. The defect labelling was accomplished by adding the defect label (detailed below) at 8 μM effective concentration to the self-assembly reaction products. In all three samples, a band is visible in the defect label emission channel that colocalizes with the target object band as seen in the object label emission channel (Fig. 1c). Importantly, the intensity of the target object band in the defect channel increased with increasing amount of unpaired DNA prepared by strand omission. Therefore, in contrast to imaging by TEM as in Fig. 1b, the defect label did capture the differences in composition of the three variants.

### A fluorescent de-Bruijn probe as defect label

What is the label for unpaired DNA? A key feature of unpaired DNA is that it can still form DNA base pairs (bp). Therefore, unpaired DNA itself can serve as a probe for complementary unpaired DNA. On the basis of target sequence space considerations (Supplementary Note 1, Supplementary Fig. 10), we developed circular de-Bruijn sequences of order 2, 3 and 4 for an alphabet of four DNA bases for our probe (Supplementary Fig. 11). In these sequences, every base type occurs with the same frequency, and also every possible subsequence of the length given by the order occurs exactly once as a sequence of consecutive characters. For chemical synthesis, the strings needed to be linearized and were split such as to reduce the propensity for forming secondary structures that could compete with the binding to the target (Supplementary Fig. 11). For example, the circular de-Bruijn sequence of order 3 with 64 bases length was split asymmetrically into two strings of length 41 and 27 bases to prevent occurrence of a hairpin motif. Four additional bases had to be introduced to restore subsequences that were lost by linearizing and splitting the de-Bruijn sequence of order 3 (Fig. 1d). All DNA oligonucleotides were synthesized and functionalized with fluorescent cyanine-3 dyes at the 5′ termini. The oligonucleotides derived from a de-Bruijn sequence of given order were each mixed in a 1:1 stoichiometry. On the basis of the sequence-matching statistics between target and probe, propensity for side reactions, signal strength and bias for AT-rich versus GC-rich unpaired DNA (Supplementary Note 1, Supplementary Figs 12–14) when compared with the de-Bruijn sequences of order 2 and 4, the oligonucleotides derived from the de-Bruijn sequence of order 3 were the best choice for the intended probe. Together, they form what we call herein the ‘defect label’.

For a quantitative evaluation of the relative amount of unpaired DNA bases, we recorded gel-electrophoretic lane intensity profiles with a multicolour fluorescence laser scanner equipped with a photomultiplier (FLA 9500, GE Healthcare). Peak intensities of the target object bands were determined in the object label (Fig. 1e) and defect label emission channels (Fig. 1f). We computed the ratio of the target band intensities in the defect label channel versus object label channel (Fig. 1g) to give the relative defect label brightness, which is proportional to the number of defect labels bound per object. When titrating the total concentration of the defect label, we observed a behaviour typical for bimolecular reactions with an apparent dissociation constant in the μM regime (Fig. 1g). Furthermore, the labelling by the defect probe was only detectable in our setup when the electrophoretic separation was done in an ice-water bath (Supplementary Figs 15 and 16). Both findings are consistent with weak binding of the defect label to short (~3 bp) DNA motifs. The labelling intensity of enzymatically prepared dsDNA plasmids (Supplementary Fig. 17) and nicked dsDNA plasmids with phosphate backbone nicks occurring every 42 bases (Supplementary Fig. 18) by the defect probe was extremely low. These data support the notion that the defect labelling occurs predominantly at unpaired DNA sites and point against alternative sources of labelling such as binding through triple-helix formation, fluorescent dye intercalation or double-helical DNA domain breathing in the target DNA. Also, the electrophoretic mobility of target objects remained unaffected by the defect label (Supplementary Fig. 19).

### Estimating the remainder of unpaired DNA

**Figure 2:**
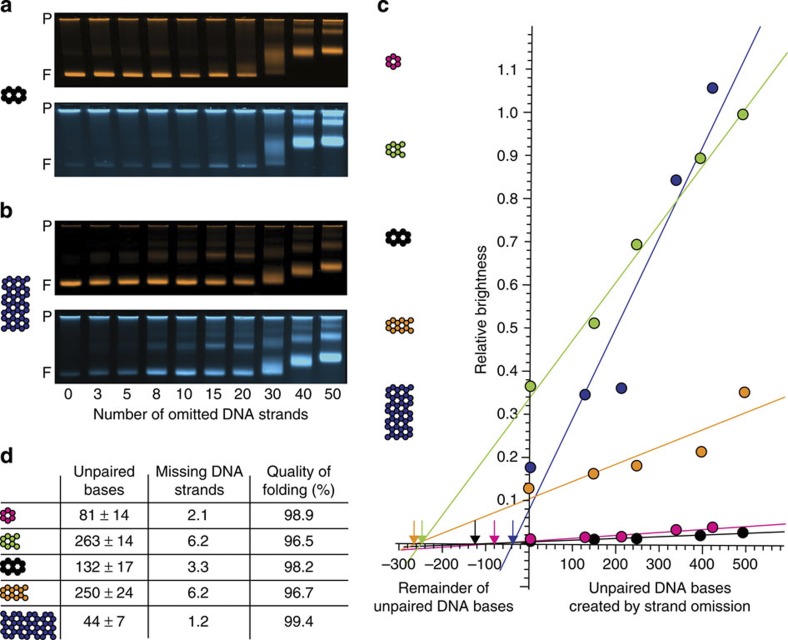
Quantifying the remainder of unpaired DNA bases in self-assembled DNA objects. (**a**,**b**) Exemplary false-coloured laser-scanned photographs of agarose gels on which pseudo-defective variants of a 10-helix DNA origami object (**a**) and a 42-helix DNA origami object (**b**) were electrophoresed. Top and bottom images show the fluorescence intensity in the object and defect label channels, respectively. P indicates the gel pocket and F, the target object band. (**c**) Relative defect label brightness (ratio of band peak intensity as recorded in the defect channel over band peak intensity in object channel) as a function of the content of unpaired DNA bases as created by omitting strands from self-assembly reactions. Coloured circles: data obtained for five different structures (6-, 8-, 10-, 12- and 42-helix bundle DNA origami objects, see coloured cross-sections). Solid lines give linear fits to the data, including an extrapolation to the negative *x* axis to determine the zero point. (**d**) Table details properties of self-assembled objects from reactions including all strands, as estimated from data in **c**. Errors are errors of the linear fit and should only be considered as the minimum extrapolation error with respect to the true unpaired DNA remainder. ‘Missing DNA strands’ were computed as an average strand equivalent by making use of the average length of staple strands in each design. The ‘quality of folding’ was defined as the ratio of formed base pairs over designed base pairs in an object under study.

We now illustrate how the defect label may be employed to estimate the remainder of unpaired DNA in self-assembled DNA objects. Our strategy relies on titrating the content of unpaired DNA elements in a given target object by omitting subsets of DNA strands from the self-assembly reactions to obtain information on the dependency of the defect labelling brightness on a known content of unpaired DNA in the object. The strategy was tested experimentally with a panel of multilayer DNA origami objects comprising 6-, 8-, 10- and 12-helix bundles that were all designed not to contain unpaired DNA elements (Supplementary Figs 20–23). Another sample was the 42-helix bundle from Fig. 1 that was planned with single-stranded TT tails at each helical interface to prevent blunt-end association. The 42-helix bundle was also designed with an improved staple strand breaking rule^25^. All objects were labelled with a single cyanine-5 dye on a selected staple strand to provide a reference signal for object concentration. Self-assembly reaction mixtures were prepared for each object that sampled an increasing amount of unpaired DNA pseudo-defects by omitting more and more DNA strands from the self-assembly reaction mixtures (Supplementary Tables 1–10). After completion of the self-assembly reactions, the products were mixed with the defect label and gel-electrophoresed side-by-side (see exemplary gel data in Fig. 2a,b and Supplementary Figs 24–28 for the source data set). For all objects studied herein, the relative defect label brightness increased fairly linearly with increasing amounts of unpaired DNA pseudo-defects in the regime of up to ~500 unpaired DNA bases (Fig. 2c). The slopes varied considerably from object to object, which we attribute mostly to variations of the fluorescence brightness of the Cy5-labelled DNA oligonucleotides that served as object concentration reference (Supplementary Fig. 29). For the evaluation of the relative brightness in the context of a pseudo-defect titration, the object label fluorescence brightness and the rate of object label incorporation does not matter as long as it remains the same among the samples that are being compared (Supplementary Fig. 30).

The defect probe also labelled objects that were prepared with unpaired DNA pseudo-defects that were placed in the interior of the object (Supplementary Figs 31 and 32 and Supplementary Tables 11 and 12). Presumably, the porous nature of the DNA objects allowed the defect label molecules to diffuse into the object’s internal cavities, in agreement with previous observations^26^.

Importantly, there was also a faint defect labelling intensity of the target object bands produced in reaction mixtures that contained all required strands (Fig. 2a–c, Supplementary Figs 24–28). This finding hinted thus at incomplete self-assembly, since these objects were designed not to contain unpaired DNA (except for the TT tails in the 42-helix bundle). By extrapolating a linear fit of the relative defect label brightness as a function of unpaired DNA bases and determining the intersection with the *x* axis, an estimate of the remainder of unpaired DNA defects can now be given (Fig. 2c,d). Accordingly, the remainder of unpaired DNA ‘defect’ bases in the objects tested herein ranged from 44 to 263 unpaired bases (in a background of 7,560 planned dsDNA bases per object), with the lowest remainder in the 42-helix bundle and the highest remainder in the 8-helix bundle. The average length of individual DNA staple strands in the folding reactions was ~42 bases. Therefore, on the average, the equivalent of 1.2 strands in the case of the 42-helix bundle or 6.2 strands in the case of the 8-helix bundle out of the 207 and 184 required DNA staple strands, respectively, failed to incorporate during folding. If we define the quality of folding as the ratio of formed dsDNA base pairs over designed dsDNA base pairs in an object, this measure was above 99% for the 42-helix bundle and 96.5% for the 8-helix bundle.

In an enzymatically prepared and thus putatively fully double-stranded DNA plasmid control, faint defect labelling was detectable when overloading the gel lane (Supplementary Fig. 17), corresponding to an extrapolated content of ~10 unpaired bases in a background of ~5,700 DNA base pairs or 99.8% quality for the plasmid. This value may indicate the upper limit of quality that may be detectable with our assay due to artefactual background labelling. Nicked dsDNA plasmids with backbone nicks occurring every 42 bases that were prepared through self-assembly of staple DNA strands on a scaffold DNA as the other multilayer DNA origami objects gave negligible signal strength in the defect label channel (Supplementary Fig. 18). An, in this case, unreliable defect extrapolation as in Fig. 2 for the nicked dsDNA plasmid yielded an estimated content of unpaired DNA between 15 and 70 bases out of 7,560 designed DNA base pairs, depending on the choice of background correction. These values correspond to an assembly quality between 99.1 and 99.8% for the nicked dsDNA plasmid, which is free from topological problems and electrostatic repulsion as it occurs in DNA origami objects.

A strong assumption that underlies the defect extrapolation is that of an one-to-one relationship between the number of bases in omitted strands and the number of unpaired DNA bases that emerge in the folded object as a consequence. This is debatable since the omission of a given strand from a reaction could potentially inhibit the incorporation of other strands that are present in the reaction. But the linear increase in defect labelling when leaving out more and more strands from the reaction also implies that the inhibitory effect would have to be fairly independent of the identity of the strand that has been left out, which we consider unlikely, given the asymmetry of the object design in terms of sequences and strand routing.

### Refining self-assembly protocols using the defect label

**Figure 3:**
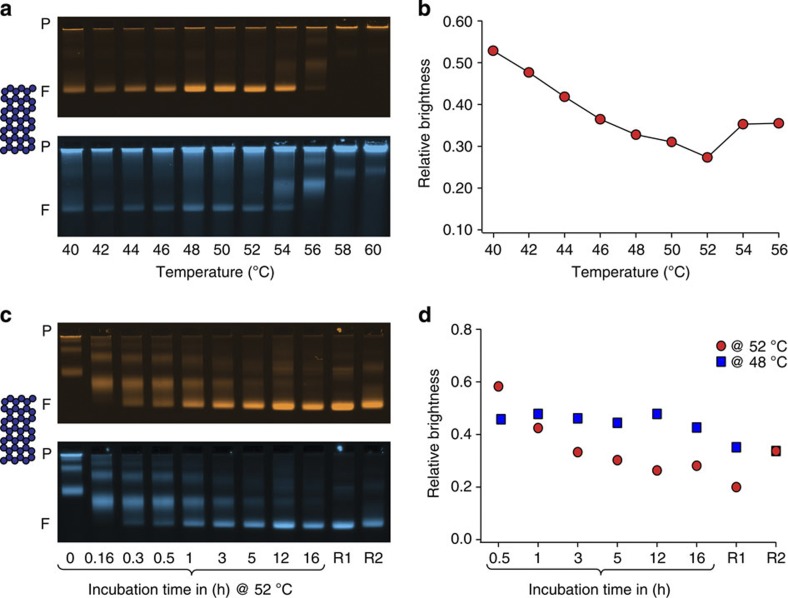
Guiding the refinement of self-assembly protocols by quality feedback. (**a**) Laser-scanned false-coloured photographs of agarose gels on which a screen of self-assembly reaction mixtures for isothermal folding of a 42-helix-bundle DNA origami object was electrophoresed. Reaction mixtures were incubated for 2 h at the indicated temperatures (after a brief scaffold-denaturing heatshock to 65 °C). Top and bottom images give object and defect label fluorescence intensities, respectively. P indicates the gel pocket and F, the target object band. (**b**) Relative defect label brightness as a function of incubation temperature. (**c**,**d**) As in **a**,**b**, but for a time-resolved analysis of a 42-helix-bundle self-assembly reaction mixture incubated at constant 52 °C. The electrophoretic mobility of the target object band displays a slight ondulation that stems from the apparatus used. R1: stepwise ‘annealing’ from 60 to 44 C° with a cooling rate of 1 °C per hour. R2: 30 min at 52 °C; 30 min at 45 °C; and 30 min at 25 °C. Note that the relative brightness values in **c**,**d** should not be compared directly because the data sets were obtained from two different gels. Blue squares give data obtained for a design variant of the 42-helix bundle that shows best constant-temperature folding at 48 °C. See also Supplementary Fig. 35.

Finally, we illustrate exemplarily how our assay may be employed to refine self-assembly protocols. As was described previously^27^, scaffolded DNA origami objects are capable of assembling at constant temperature, which allowed for reducing reaction times as compared with annealing-based protocols while at the same time providing high yields of assembled objects. The success of assembly was previously judged by electrophoretic mobility and TEM imaging alone^27^, which may not have resolved subtle differences in the quality of the reaction products. By using the defect label, this property can now be assessed in greater detail. Therefore, we have setup an exemplary screen of constant-temperature self-assembly reactions with a duration of 2 h for making two versions of a 42-helix bundle object and analysed the products with the defect label as above (Fig. 3a, see also Supplementary Figs 33 and 34 for complete gel images and Supplementary Fig. 35 for results with the other variant). For the variant analysed in Fig. 3, the highest yield (as judged by band brightness in the object channel) and the best folding quality (as judged by the lowest relative defect labelling intensity) were both achieved in the reaction performed at 52 °C (Fig. 3a,b). The other 42-helix bundle variant folded best at 48 °C (Supplementary Fig. 35). A time-resolved analysis of the evolution of assembly quality at 52 °C of the 42-helix bundle version that folded best at 52 °C (Fig. 3c, see Supplementary Fig. 34 for full gel images) revealed a subtly decreasing defect labelling brightness on the timescale of hours (Fig. 3d), but it did not reach the slightly lower level that was obtained when subjecting the reaction mixture to a 16 h long thermal annealing ramp (Fig. 3d, R1). A similar trend, but less pronounced, was observed for the other 42-helix bundle version that folded best at 48 °C. The difference in defect labelling intensity corresponded to an estimated equivalent of two to three staple strands that did not incorporate (out of a pool of 207 strands) in the reaction products from the constant temperature reaction as compared with the products from traditional annealing. This finding indicated that the 42-helix bundle design features some subtle details whose realization requires special attention during practical synthesis. A refined 1.5 h three-temperature-step reaction protocol then yielded products with a quality that appeared comparable to that obtained from 16-h long annealing (Fig. 3d, R2).

## Discussion

To conclude, we have presented a method for quantifying the quality of assembly for DNA nanostructures in terms of unpaired DNA bases. The assay is easy to apply and works in conjunction with the standard method of analysis in the field of DNA nanotechnology, namely, gel electrophoresis. The quality of assembly of multilayer DNA origami nanostructures that we estimated herein was satisfyingly high, thus underlining the capability of DNA nanotechnology for producing well-defined high-quality objects that can meet the designer’s compositional specifications. Our assay helps rationalizing the refinement of assembly protocols, which is especially important when testing new design rules for which the requirements for practical assembly are at first unknown. Beyond DNA nanotechnology, we speculate that the ability to discriminate unpaired nucleic acids in the context of paired nucleic acids within the same macromolecular object may also open interesting perspectives for testing cellular nucleic acids for this property (Supplementary Fig. 36), but this possibility remains to be explored.

## Methods

### Molecular self-assembly with scaffolded DNA origami

Structures were designed using caDNAno v.02^28^. DNA scaffold strands of 7,560 bases length derived from the genome of bacteriophage M13 were prepared recombinantly as described previously^20^. Staple oligonucleotide strands were prepared by solid-phase chemical synthesis (Eurofins MWG, Ebersberg, Germany, HPSF grade). Production of the multihelix bundles was accomplished in one-pot reaction mixtures. The reaction mixtures contained scaffold strands at a concentration of 50 nM and oligonucleotide strands at 200 nM each. To create single-stranded DNA pseudo-defects, a subset of the required oligonucleotides was omitted from the assembly reactions. The cyanine-5-modified object label oligonucleotide was included in the reaction mixture at a concentration of 200 nM. The buffer that was used included 5 mM TRIS, 1 mM EDTA, 20 mM MgCl_2_ and 5 mM NaCl (pH 8). The reaction mixtures were subjected to a thermal annealing ramp using TETRAD (MJ Research, now Biorad) thermal cycling devices. If not otherwise noted, the reaction mixtures were first incubated at 65 °C for 15 min and then cooled from 60 to 44 °C in steps of 1 °C per hour. The reaction products were stored protected from light in a refrigerator at around 4 °C.

### Incubation with defect probe

Reaction products (multi-helix bundles) were incubated with the cyanine-3-modified defect label at room temperature. The final incubation mixture contained folded DNA objects at concentrations of ~25 nM. The total concentration of the defect label in the incubation mixture was set to 8 μM. Each oligonucleotide of the defect label was set to the same concentration (i.e. 4 μM in the case of the de-Bruijn probe of order 3 that consisted of 2 oligonucleotides). If not indicated otherwise, the incubation was done for 1–2 h.

### Gel electrophoresis

The folded DNA nanostructures incubated with defect label were electrophoresed on 3% agarose gels containing 0.5 × TBE (1 mM EDTA, 44.5 mM Tris base, 44.5 mM Boric acid) and 11 mM MgCl_2_ for around 3.5 h at 90 V in a gel box immersed in an ice-water bath. If not otherwise specified.

### TEM and image processing

Unpurified reaction products were adsorbed on glow-discharged formvar-supported carbon-coated Cu400 TEM grids (Science Services, Munich, Germany) and stained using a 2% aqueous uranyl formate solution containing 25 mM sodium hydroxide. Imaging was performed using a Philips CM100 EM operated at 100 kV. Images were acquired using an AMT 4 Megapixel charge-coupled device camera. Micrograph scale bars were calibrated by imaging 2D catalase crystals and using the lattice constants as length reference. Imaging was performed at × 28,500 magnification. For image processing, libraries of individual particle micrographs were created by particle picking using the EMAN2^29^ boxing routine. Generation of average particle micrographs was performed using IMAGIC (Image Science, Berlin) cross-correlation algorithm. To this end, 250 particles from each sample (as shown in Supplementary Figs 4–9) were aligned to a randomly picked reference particle via rotation and translation operations. In a next step, the individual particle images were averaged. Thus, for every sample an averaged image was obtained. These images were finally aligned and contrast-adjusted relative to each other.

### Analysis of gel-electrophoresis data

The electrophoresed agarose gels were scanned using a Typhoon 9500 FLA laser scanner (GE Healthcare) at a resolution of 50 μm per px. The resulting 16-bit tif images were analysed using Igor Pro V6.22 (Wavemetrics). The analysis was done with a script written in Igor Pro. For each lane that contained sample, a cross-sectional intensity profile was calculated by averaging over grayscale values within a 60-pixel wide box.

The peak intensity of the target band was determined in both channels (defect label and object label). The relative brightness was calculated by division of the maximum intensity in defect channel through the maximum intensity in the object channel.

### Construction of de-Bruijn sequences

A de-Bruijn sequence^30^ of order *k* on an alphabet of length (number of symbols) *n* is a cyclic sequence that contains every subsequence of length *k* (every *k*-mer, in total: *n*^*k*^) exactly once. In our case, the alphabet consists of the four (*n*=4) nucleobases {A, C, G, T}. The length of the (circular) sequence is *n*^*k*^. A linear sequence of length *m* contains only (*m*−*k*+1) subsequences of length *k*. Thus, additional bases have to be added when the sequence is linearized. The length of a linearized de-Bruijn sequence is *n*^*k*^+*k*−1.

We generated de-Bruijn sequences using a recursive algorithm that generated sequences by adding one letter (nucleobase) at each recursion step. The recursion was terminated as soon as the created sequence did not fulfil the de-Bruijn condition anymore (that is, as soon as at least one *k*-mer was present more than once in the sequence). Thus, only sequences fulfilling the de-Bruijn property were propagated. The generation of a de-Bruijn sequence (containing all possible *k*-mers exactly once) was completed as soon as the recursion depth (and thus the sequence length) was equal to the length of a de-Bruijn sequence ((*n*^*k*^+*k*−1) for the linearized variant).

This algorithm can be implemented easily, however, for large values of *k* it is advisable to use approaches based on de-Bruijn graphs.

A de-Bruijn graph of dimension *k* on an alphabet with *n* letters is a directed graph whose number of vertices corresponds to the number of distinct *k*-mers (*n*^*k*^). Directed edges from one vertex to another exist if the (*k*−1)-letter long suffix of the former is equal to the (*k*−1)-letter long prefix of the latter. The number of edges is thus (*n*^(*k*+1)^) and corresponds to the number of vertices in a (*k*+1)-dimensional de-Bruijn graph. De-Bruijn sequences of order *k* can for example be constructed by taking a path that visits each of the *n*^*k*^ vertices of a *k*-dimensional de-Bruijn graph exactly once (Hamiltonian path). Another way to construct a de-Bruijn sequence of order *k* is to follow a path that visits each of the *n*^*k*^ edges of a (*k*−1)-dimensional de-Bruijn graph exactly once (Eulerian path).

Efficient algorithms exist only for the latter construction method. A nice overview over de-Bruijn graphs, their usage in the construction of de-Bruijn sequences and their application in genome assembly can be found in ref. 31.

## Author contributions

C.H.W. and K.F.W. performed the research and H.D. designed the research. All authors analysed and discussed the data. H.D. wrote the paper, and C.H.W. and K.F.W. commented on the manuscript.

## Additional information

**How to cite this article:** Wagenbauer, K. F. *et al.* Quantifying quality in DNA self-assembly. *Nat. Commun.* 5:3691 doi: 10.1038/ncomms4691 (2014).

## Supplementary Material

Supplementary Information Supplementary Figures 1-36, Supplementary Tables 1-14, Supplementary Note 1 and Supplementary Reference
