# Supplementary material for: Quantifying quality in DNA self-assembly
Source: Nat Commun. 2014 Apr 22;5:3691. doi: 10.1038/ncomms4691 (PMC3997801; doi:10.1038/ncomms4691)
Supplement: Supplementary Information — Supplementary Figures 1-36, Supplementary Tables 1-14, Supplementary Note 1 and Supplementary Reference [file ncomms4691-s1.pdf]

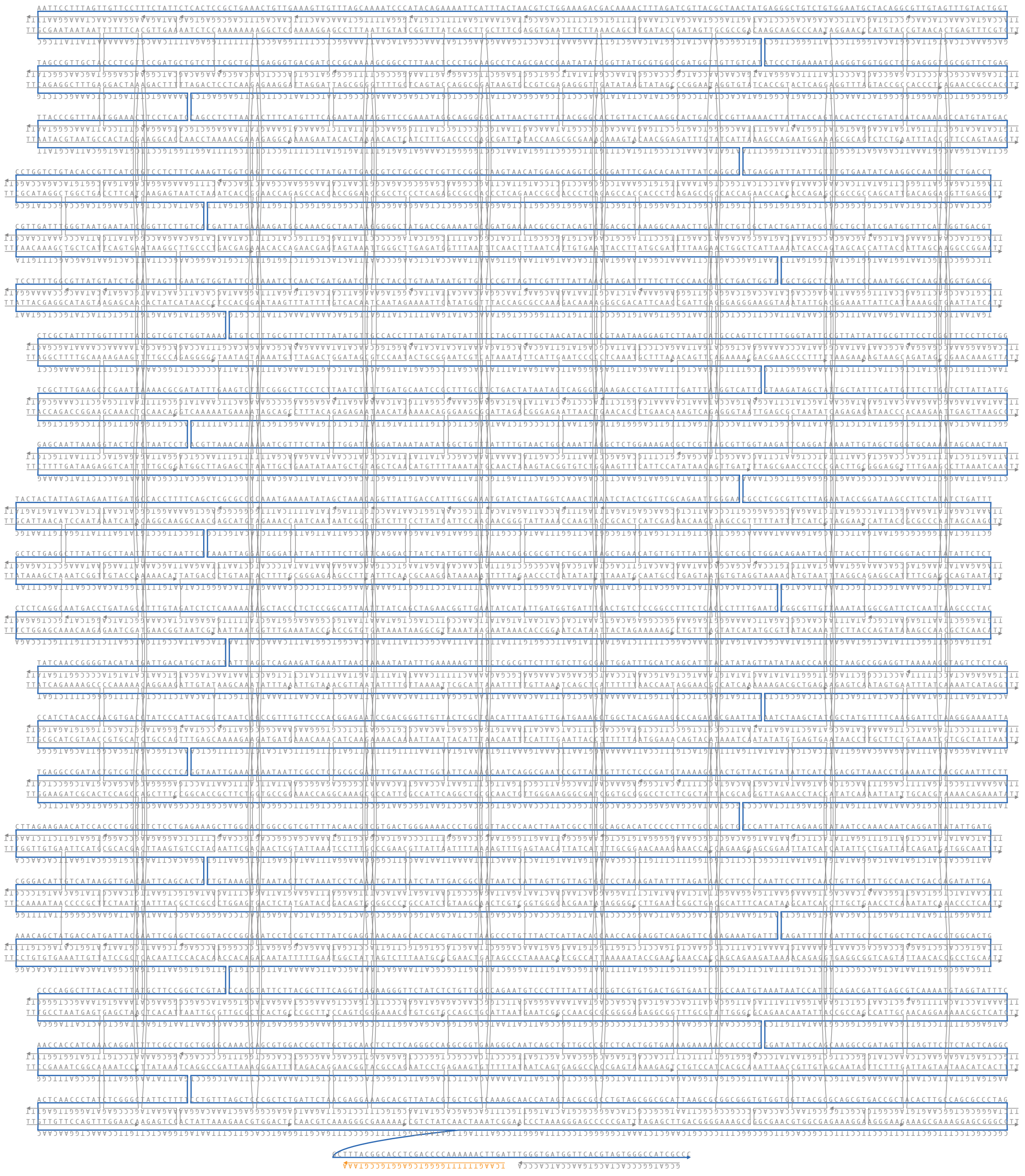

Supplementary Figure 1: Scaffold / staple layout of the 42-helix bundle object. Generated with caDNAno v0.2. Object-label is colored orange.

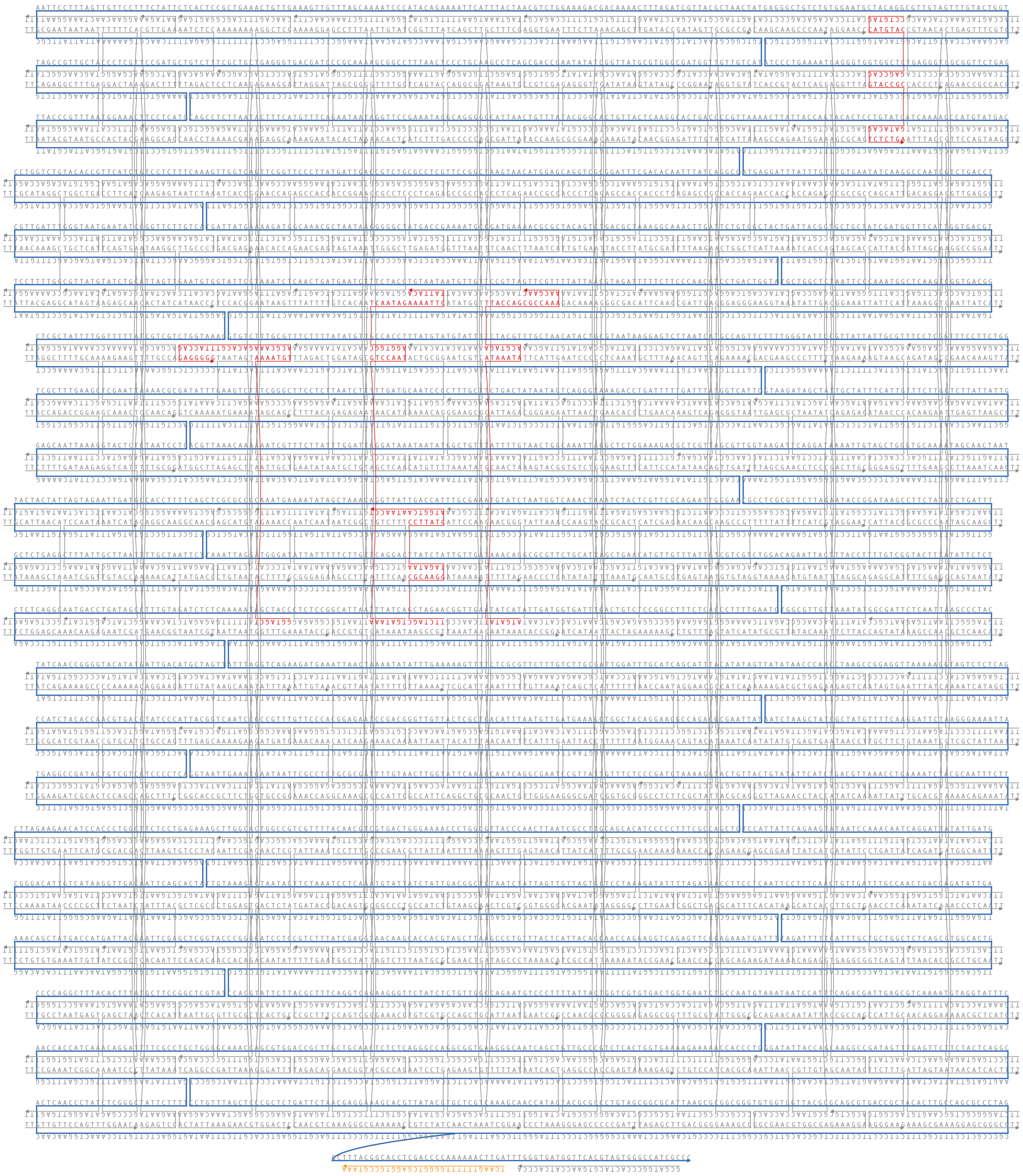

Supplementary Figure 2: Scaffold / staple layout of the 42-helix bundle object (pseudo-defect-variant with 5 defects). Generated with caDNAno v0.2. Object-label is colored orange. Omitted staples are depicted in red.

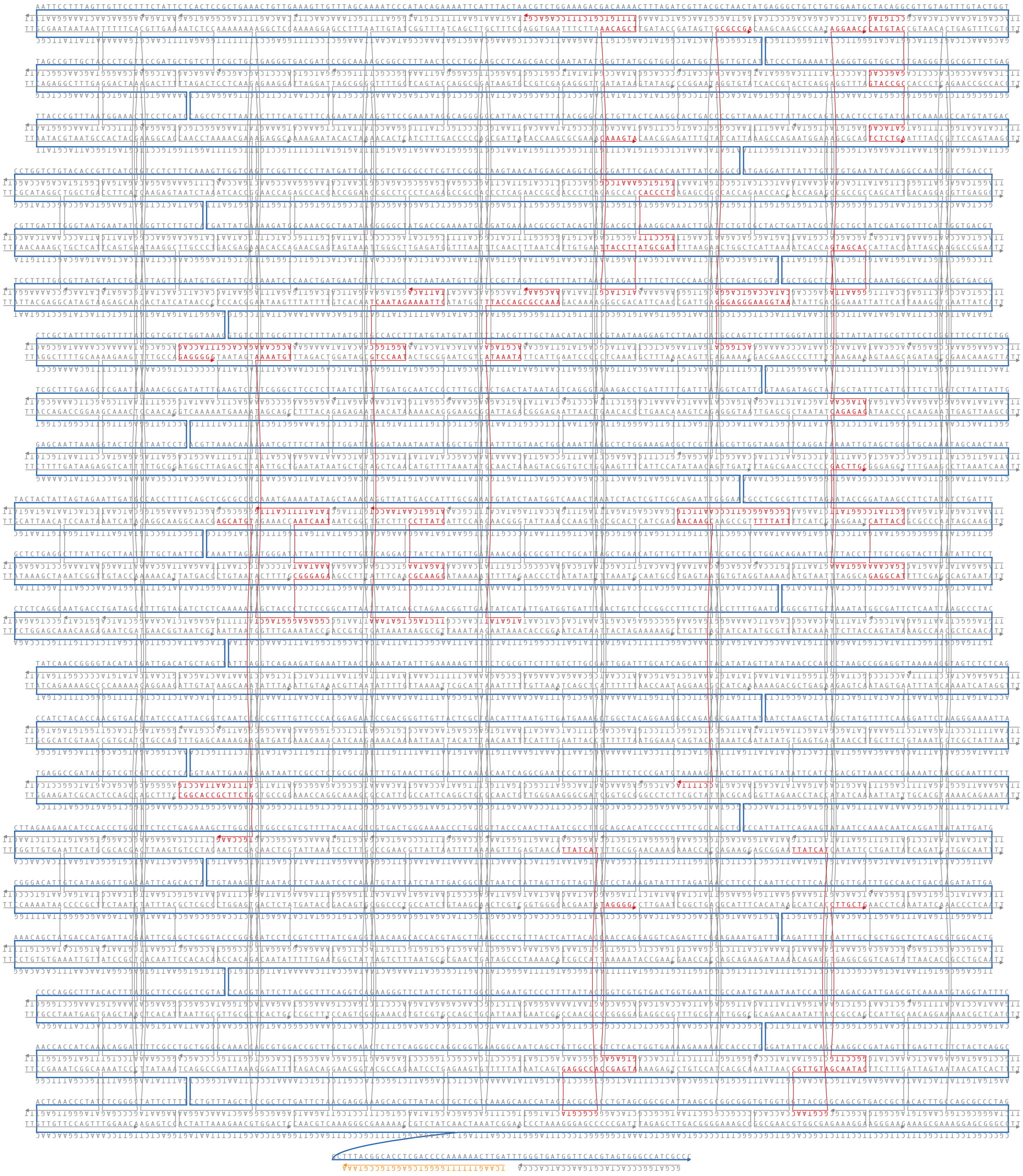

Supplementary Figure 3: Scaffold / staple layout of the 42-helix bundle object (pseudo-defect-variant with 15 defects). Generated with caDNAno v0.2. Object-label is colored orange. Omitted staples are depicted in red.

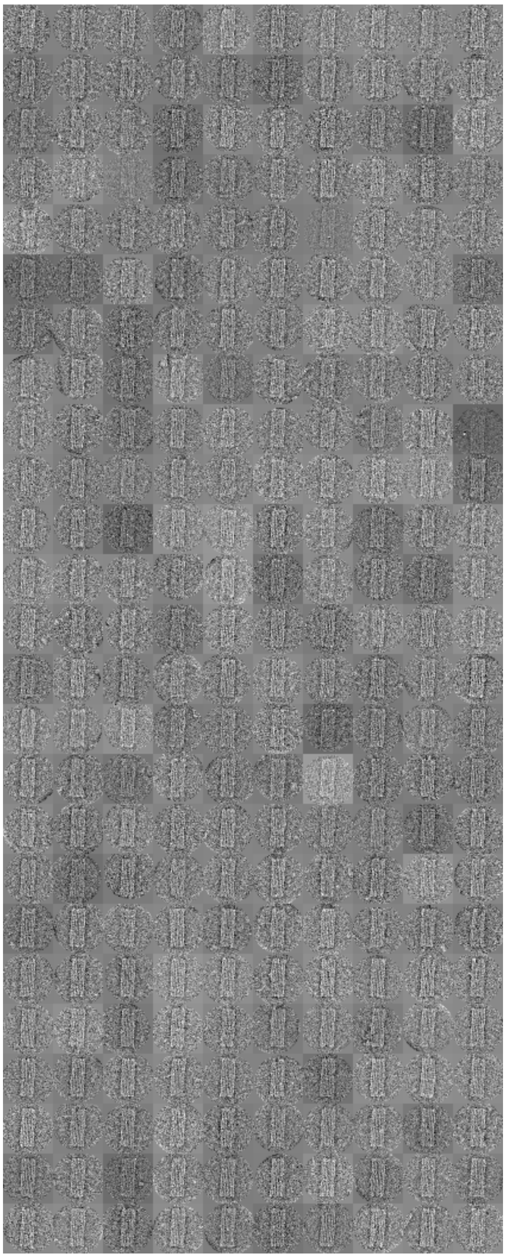

Supplementary Figure 4: Negative-stain TEM-images of the 42-helix-bundle object with 0 pseudo-defects.
Image size = 85 x 85 nm. View 1 (see fig. 1a)

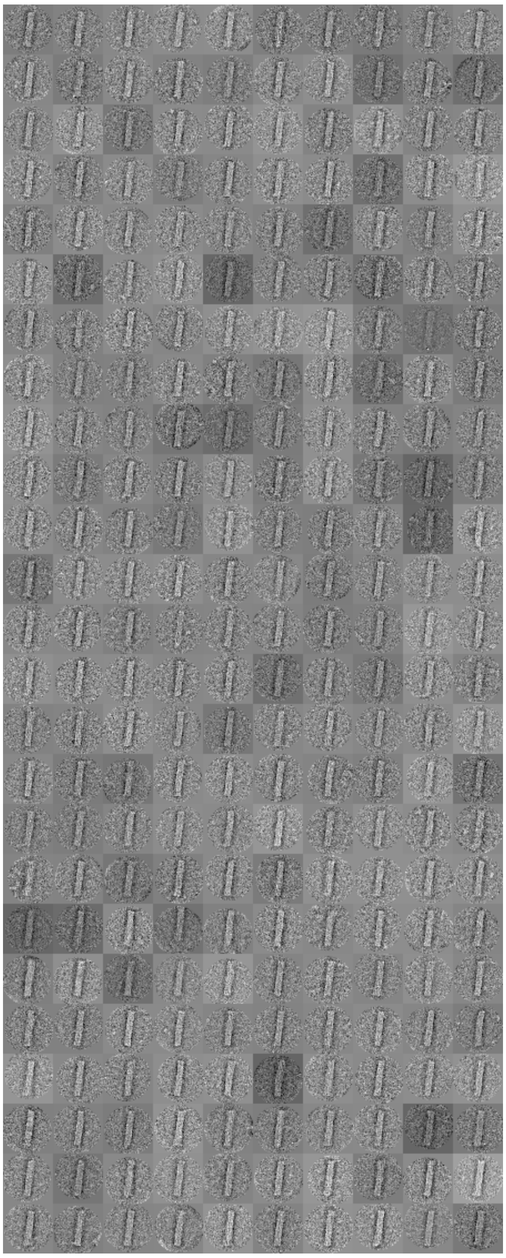

Supplementary Figure 5: Negative-stain TEM-images of the 42-helix-bundle object with 0 pseudo-defects.
Image size = 85 x 85 nm. View 2 (see Fig. 1a)

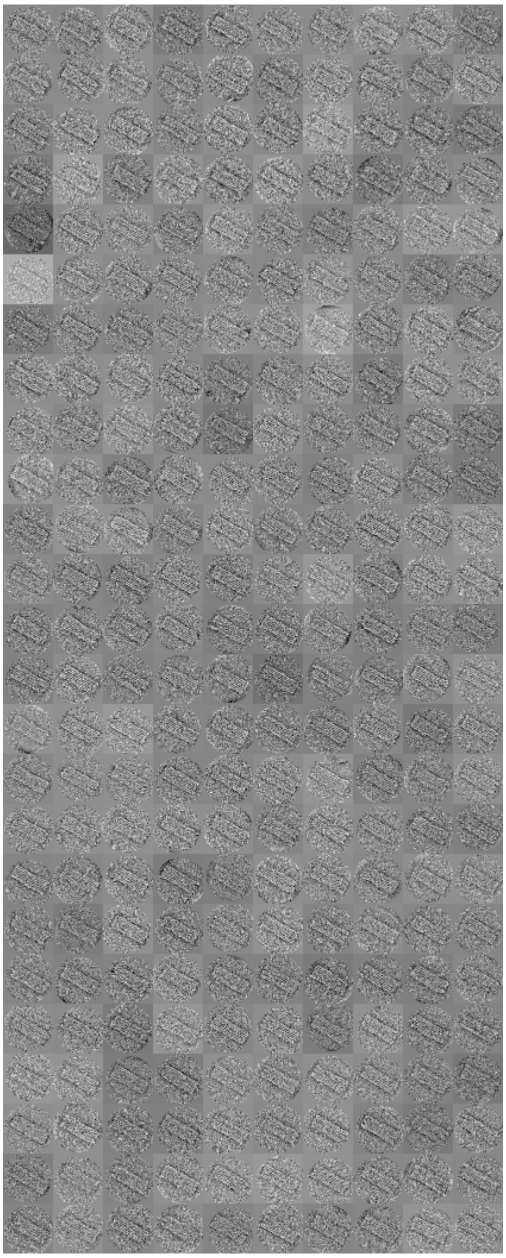

Supplementary Figure 6: Negative-stain TEM-images of the 42-helix-bundle object with 5 pseudo-defects.
Image size = 85 x 85 nm. View 1 (see fig. 1a).

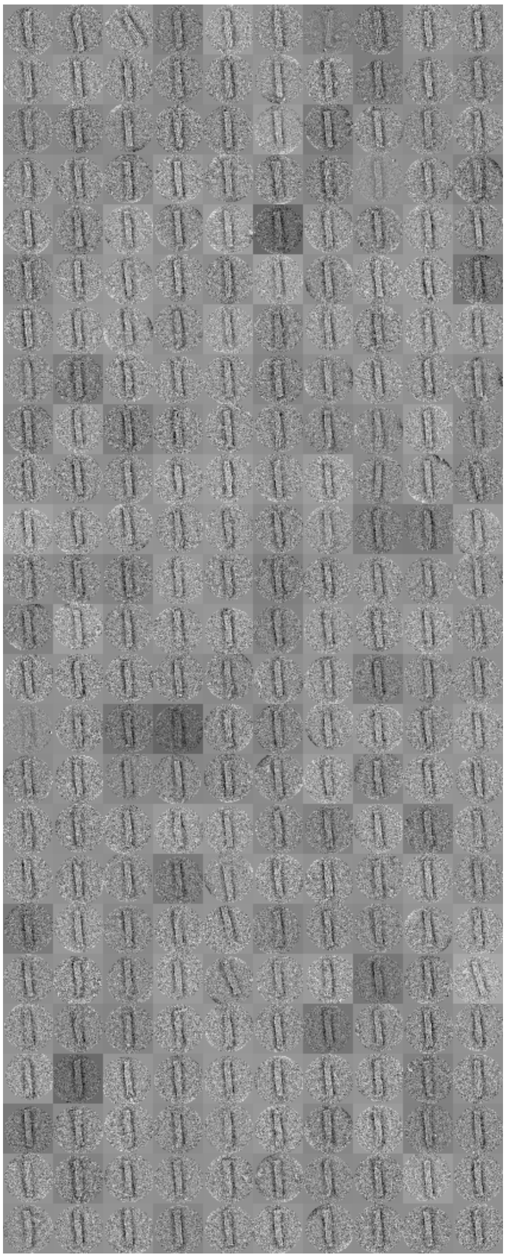

Supplementary Figure 7: Negative-stain TEM-images of the 42-helix-bundle object with 5 pseudo-defects.
Image size = 85 x 85 nm. View 2 (see fig. 1a)

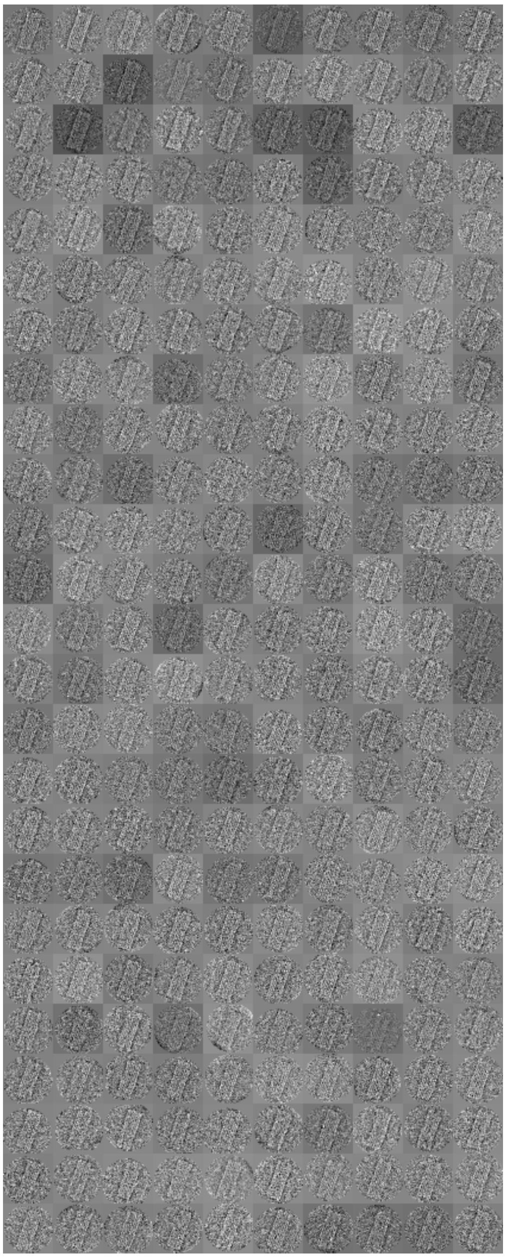

Supplementary Figure 8: Negative-stain TEM-images of the 42-helix-bundle object with 15 pseudo-defects.
Image size = 85 x 85 nm. View 1 (see fig. 1a).

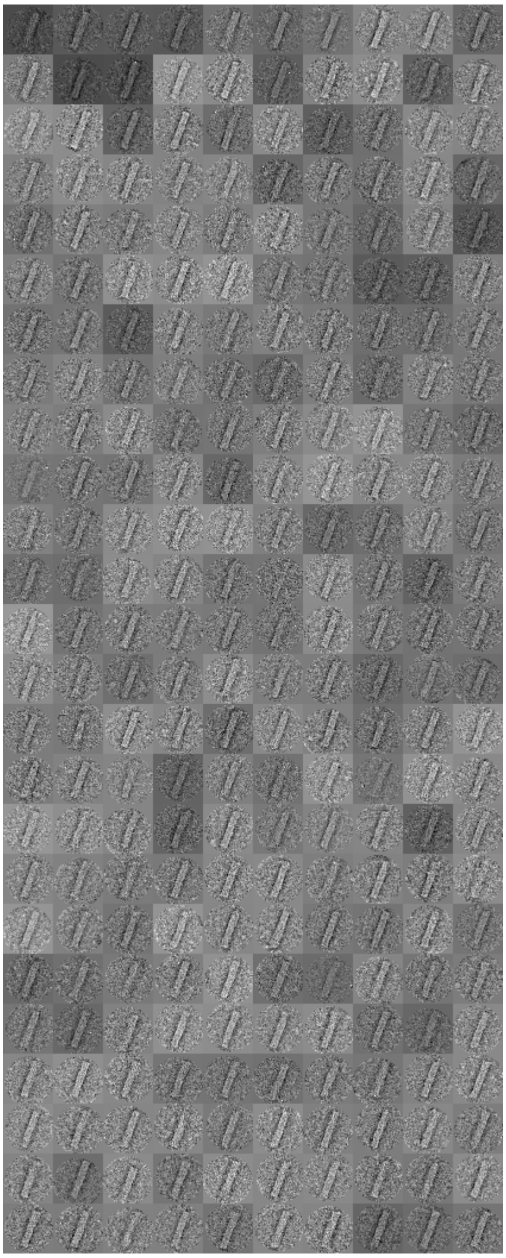

Supplementary Figure 9: Negative-stain TEM-images of the 42-helix-bundle object with 15 pseudo-defects.
Image size = 85 x 85 nm. View 2 (see fig. 1a).

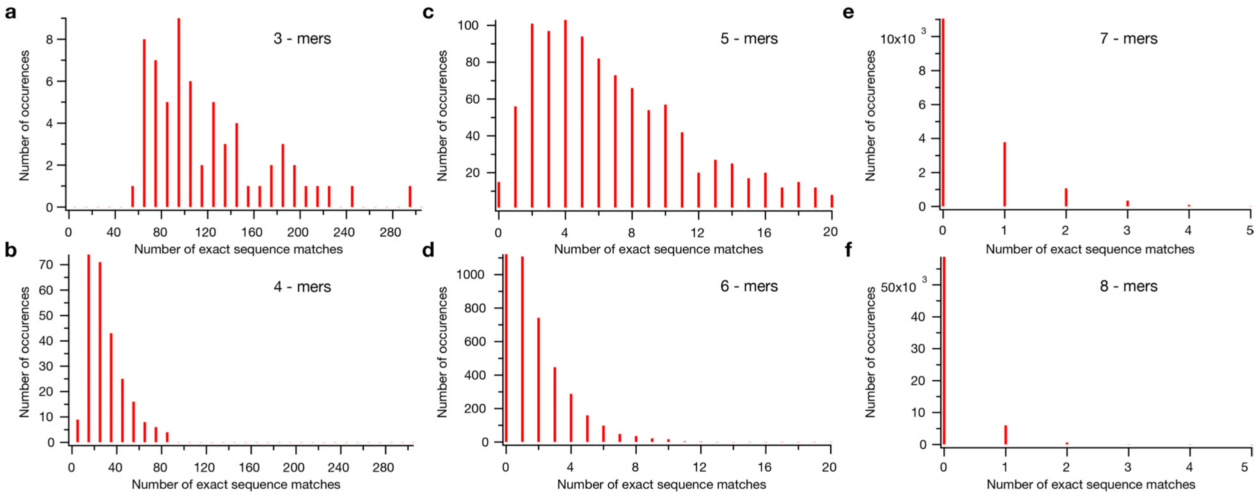

Supplementary Figure 10: Analysis of the target sequence space in the scaffold DNA. Scaffold DNA was searched for all 4^N N-base long sequence strings and the number of exact matches was counted. Histogram give the frequency by which a certain number of exact matches was observed. Please see discussion in the supplementary text.

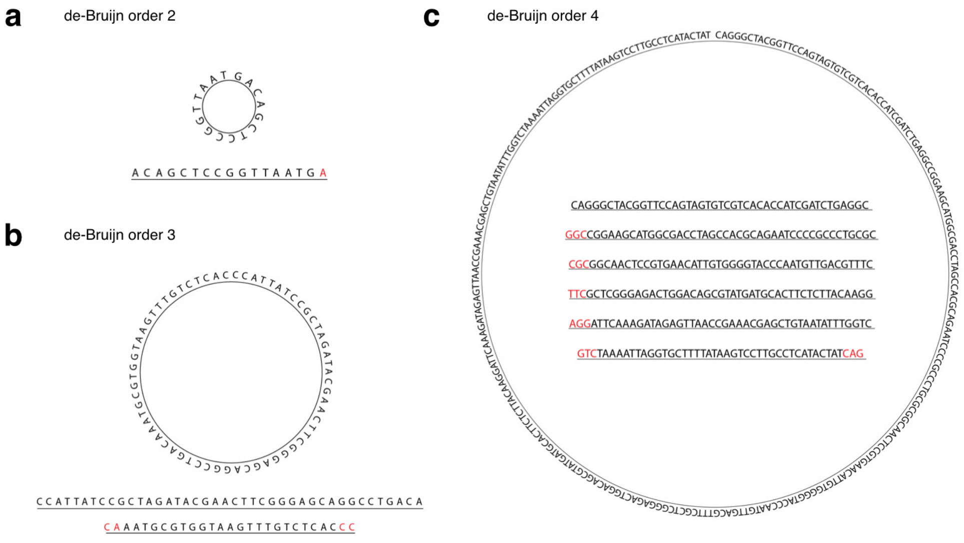

Supplementary Figure 11: Circular de-Bruijn sequence strings of order 2, 3, and 4 and the linearized sequence strings that were chemically synthesized. Bases marked in red were added to restore sequence strings that are destroyed when linearizing the circular de-Bruijn graph.

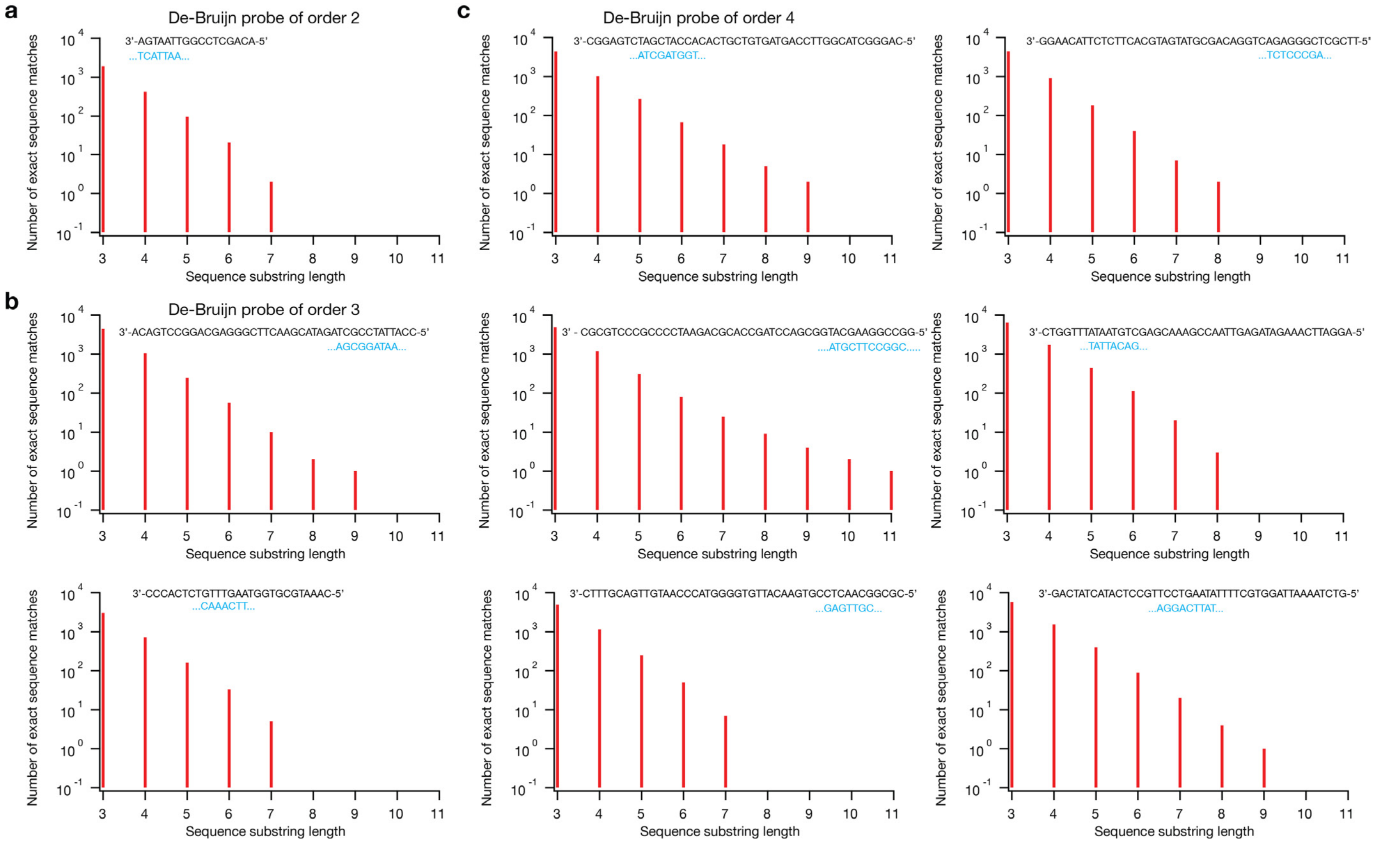

Supplementary Figure 12: Statistics of exact complementary sequence matches between target DNA and de-Bruijn probe sequences from Fig. S11. One of the longest match string is indicated in each panel in blue.

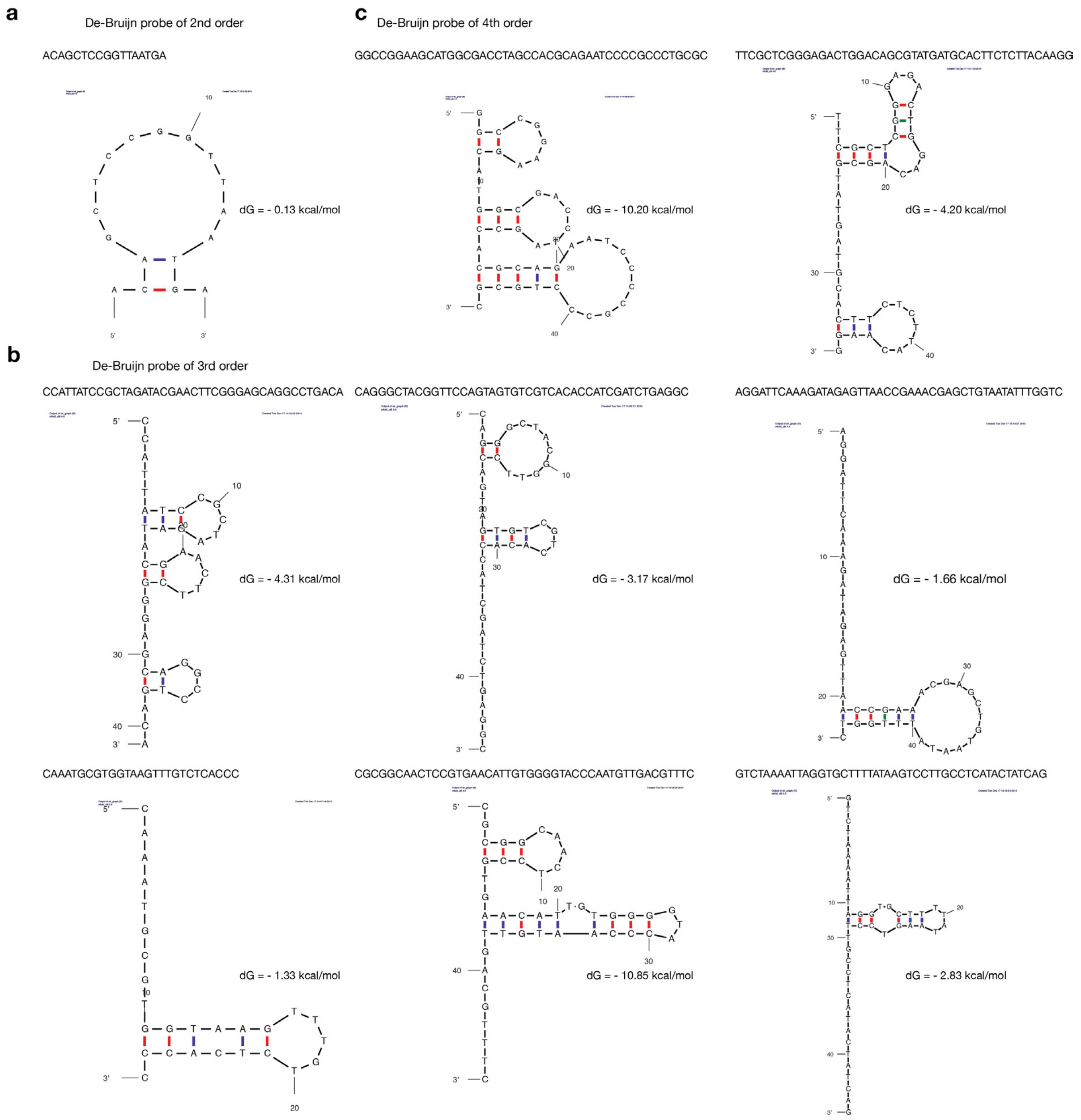

Supplementary Figure 13: Lowest-energy secondary structures in de-Bruijn probes of order two (a), three (b), and four (c) according to an analysis using mfold [1].

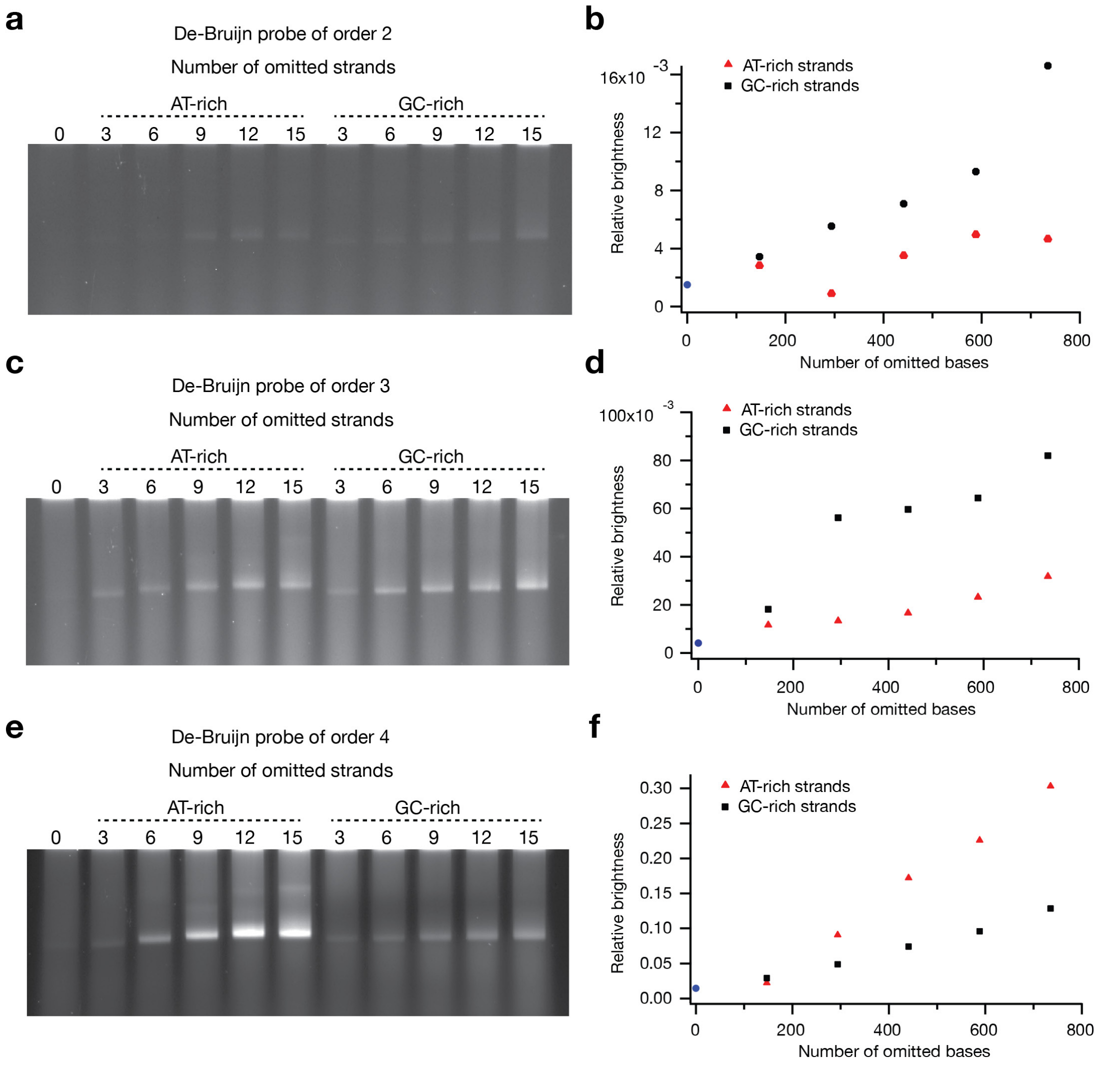

Supplementary Figure 14: Testing de-Bruijn probes of order 2 to 4 for bias against labeling AT-rich versus GC-rich defects (see Supplementary Tables 13, 14 for lists of the corresponding oligonucleotides with highest AT and GC-content).

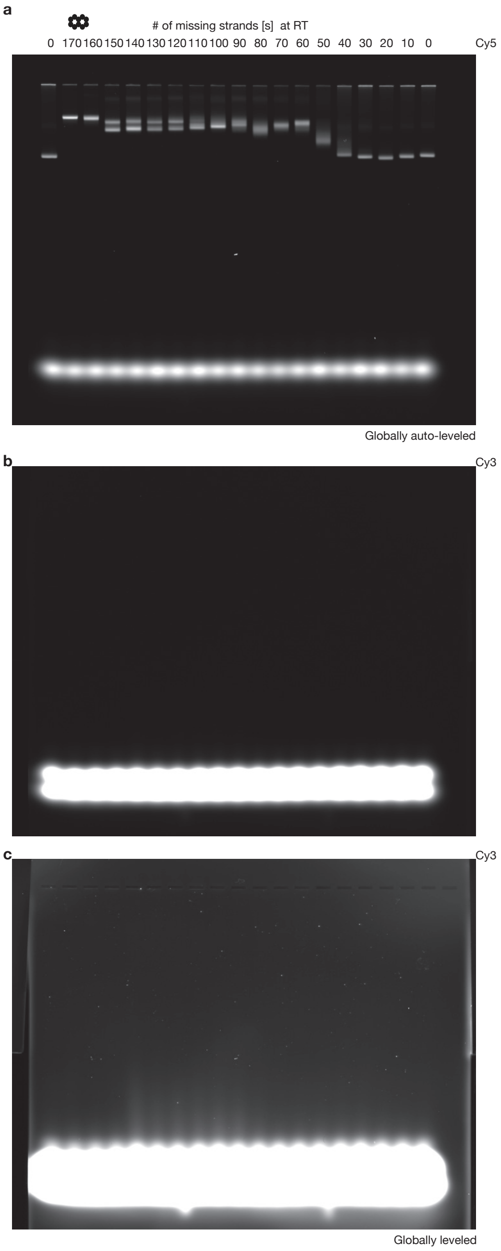

Supplementary Figure 15: Agarose-gel images for ten-helix bundle object variants. Gel-box was immersed in water-bath without ice. Cy5 (object label) and Cy3 (defect label) channel (two different brightness-adjustments) are shown. RT = room temperature.

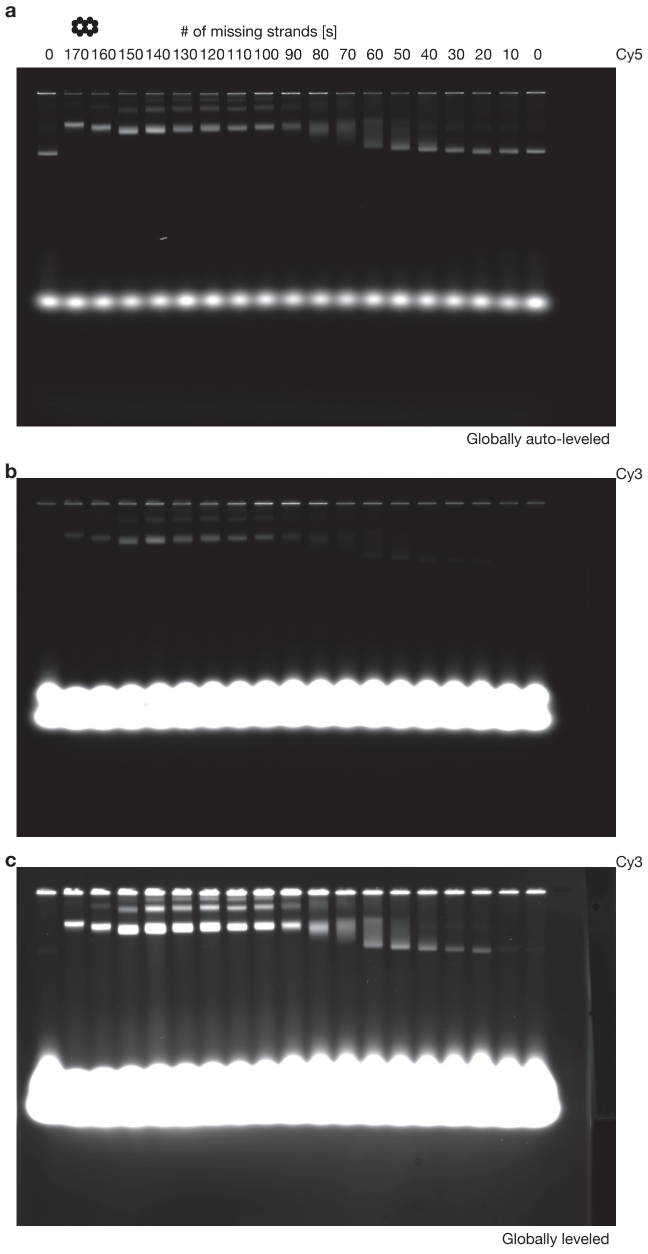

Supplementary Figure 16: Agarose-gel of ten-helix bundle object variants. Same samples as in Supplementary Figure 10. Gel-box was immersed in ice-water-bath. Cy5 and Cy3-channel (two different brightness-adjustments) are shown.

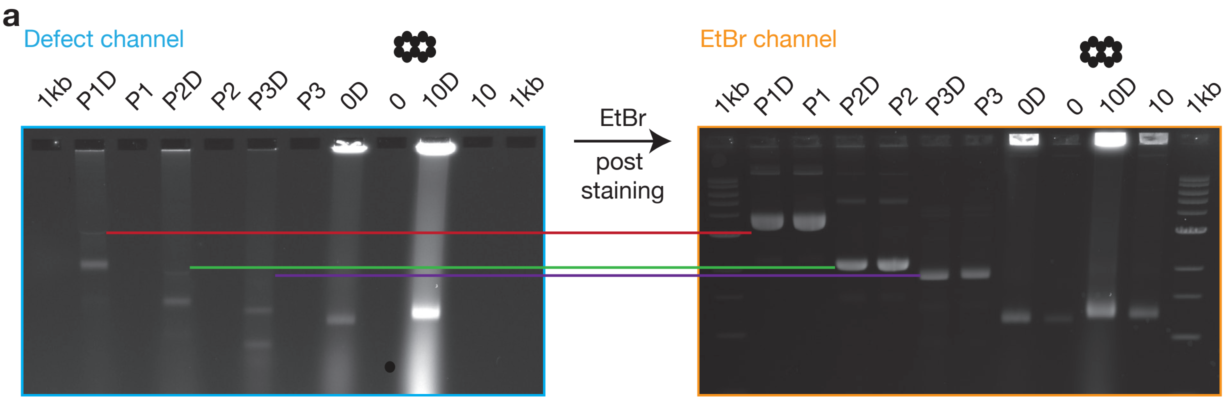

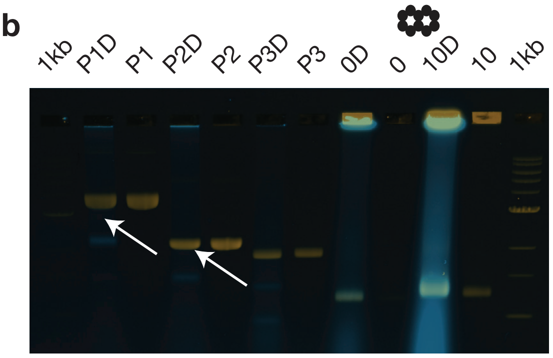

c

	10hb 0D	P1
Band intensity defect channel [cts]	122	10
Band intensity EtBr channel [cts]	1424	1928
Relative brightness	0.065	0.005
Eqv. missing DNA bases	130	10
Quality [%]	98.2	99.8

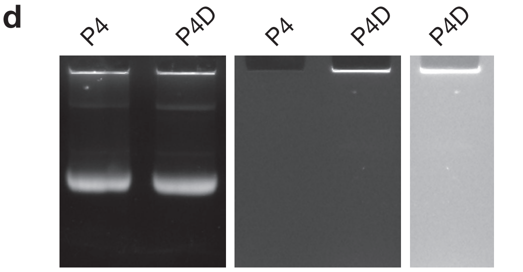

Supplementary Figure 17: Labeling dsDNA plasmids with the de-Bruijn probe. (a) Three dsDNA plasmids with various lengths were electrophoresed together with the de-Bruijn probe of order 3. The gel was laser scanned in the de-Bruijn emission channel (left image), then soaked in EtBr solution, and scanned again (right image). Only samples labeled with “D” were incubated with the de-Bruijn probe. The dominant plasmid bands in the right image (P1D - P3D) are labelled very faintly by the de-Bruijn probe only at its leading edge. Also, additional bands show up that are not resolvable in the EtBr channel. These bands most likely arise from single-stranded nucleic acids contaminants. As positive controls for defect labeling, a ten-helix bundle with zero missing staple strands (0) and a version with 10 missing staple strands were also included. The bright leading bands that correspond to folded 10-helix bundle objects appear in both channels. (b) False-colored channel merge image. (c) Table gives peak intensities at the indicated bands. Based on the known defect content of the 10 helix bundle sample (see Fig 2.), the faint defect labeling intensity of the plasmid P1 was converted into an estimated amount of missing bases. (d) Another plasmid control was electrophoresed together with de-Bruijn probes. In this case zero defect labeling is detected.

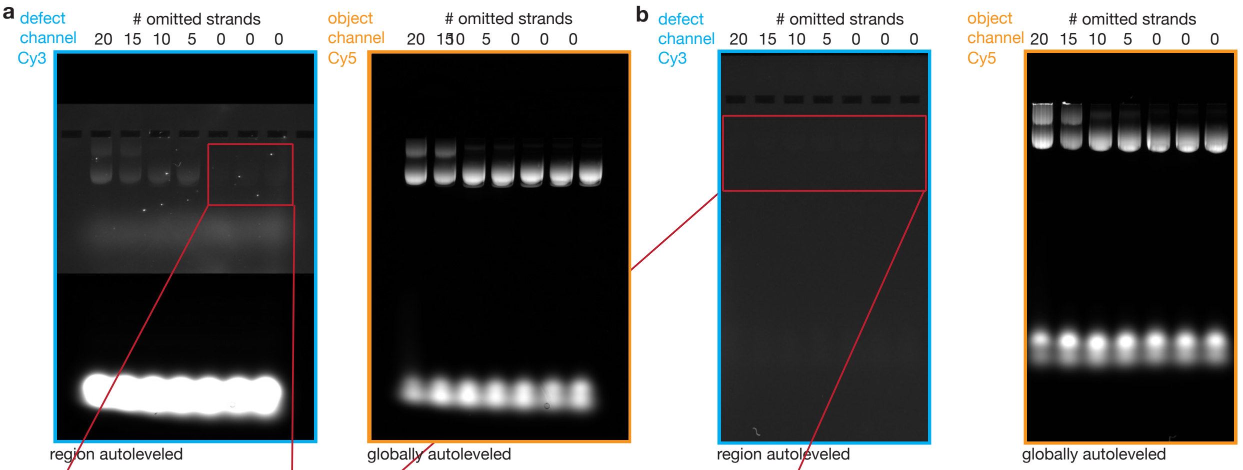

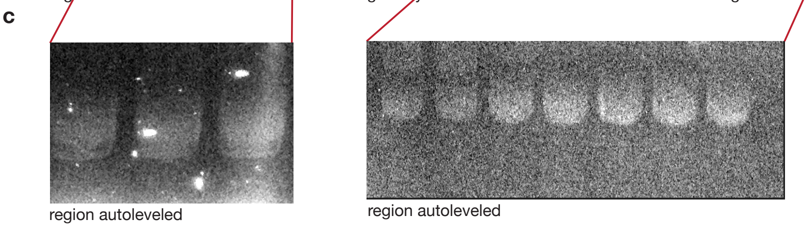

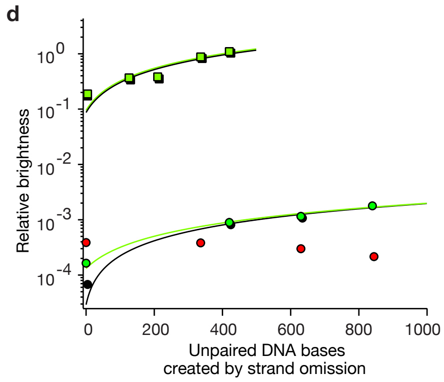

e

	6 hb	8hb	10 hb	12 hb	42 hb	1 hb
Band intensity defect channel [cts]	632	429	42	45	384	1 (4)
Band intensity object channel [cts]	3903	1172	6341	349	2164	51442 (52772)

Supplementary Figure 18: Labeling a nicked dsDNA control structure with the de-Bruijn probe. The construct was composed of DNA scaffold and 180 DNA staples with 42 bases length each that do not undergo any crossovers. Therefore, the construct consists of a one-dimensional circular array of 42-basepairs long double-helical DNA domains that are delimited by phosphate backbone nicks. (a) The nicked dsDNA control was electrophoresed on a 3% agarose gel together with the de-Bruijn probe of order 3 and laser scanned in the Cy3 and in the Cy5 emission channel. (b) The nicked dsDNA control was electrophoresed on a 3% agarose gel together *without* the de-Bruijn probe of order 3 and laser scanned in the Cy3 and in the Cy5 emission channel to evaluate channel crosstalk. (c) Emission channel leakage from Cy5 into Cy3 filter sets explains the residual band intensity in the cy3 emission channel when only a cy5 emitter is included in the reaction. (d) Defect titration as in Fig (2) main with the nicked dsDNA control (circles) and the 42 helix bundle sample (squares). Red circles give relative brightness obtained from channel bleedthrough in b. Two methods of correcting for background were employed (subtracting background in front of the band under study, black data; and subtracting background from a sample free region in the gel, green data). Solid lines give linear fits to the data. For the nicked dsDNA sample: black line intersects at -15±5 bases, green line intersects at -70±7 bases. For the 42 helix bundle sample: both lines intersect at -44±7 bases. Errors give error of the fit. (e) Table gives absolute signal strengths in the defect label channel obtained for various all-staple DNA origami reaction products after correcting for background by subtracting counts in front of the band under study. The nicked dsDNA plasmid (denoted as 1hb) had significantly lower signal strength. Value in parentheses indicates counts when using a different method of background correction (green method in d)).

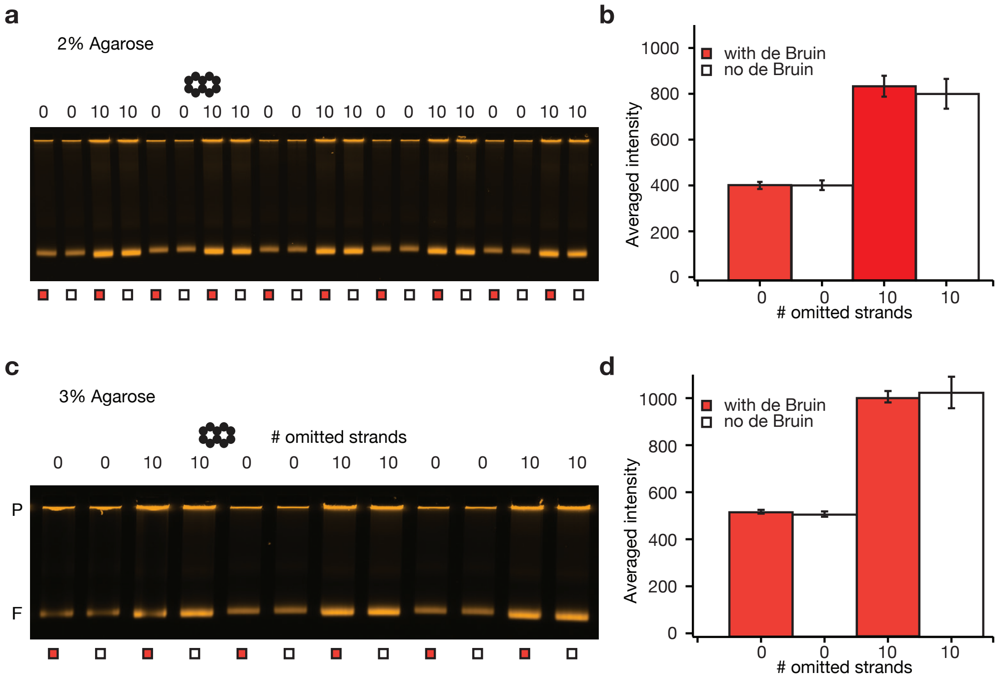

Supplementary Figure 19: The defect label does not affect the electrophoretic mobility of folding products. Versions of a non-“defective” and a defective 10-helix-bundle were electrophoresed on 2% and 3% agarose gels with and without the de-Bruijn probe of order 3 at 8μM concentration. No difference can be discerned between the labeled vs unlabeled lanes.

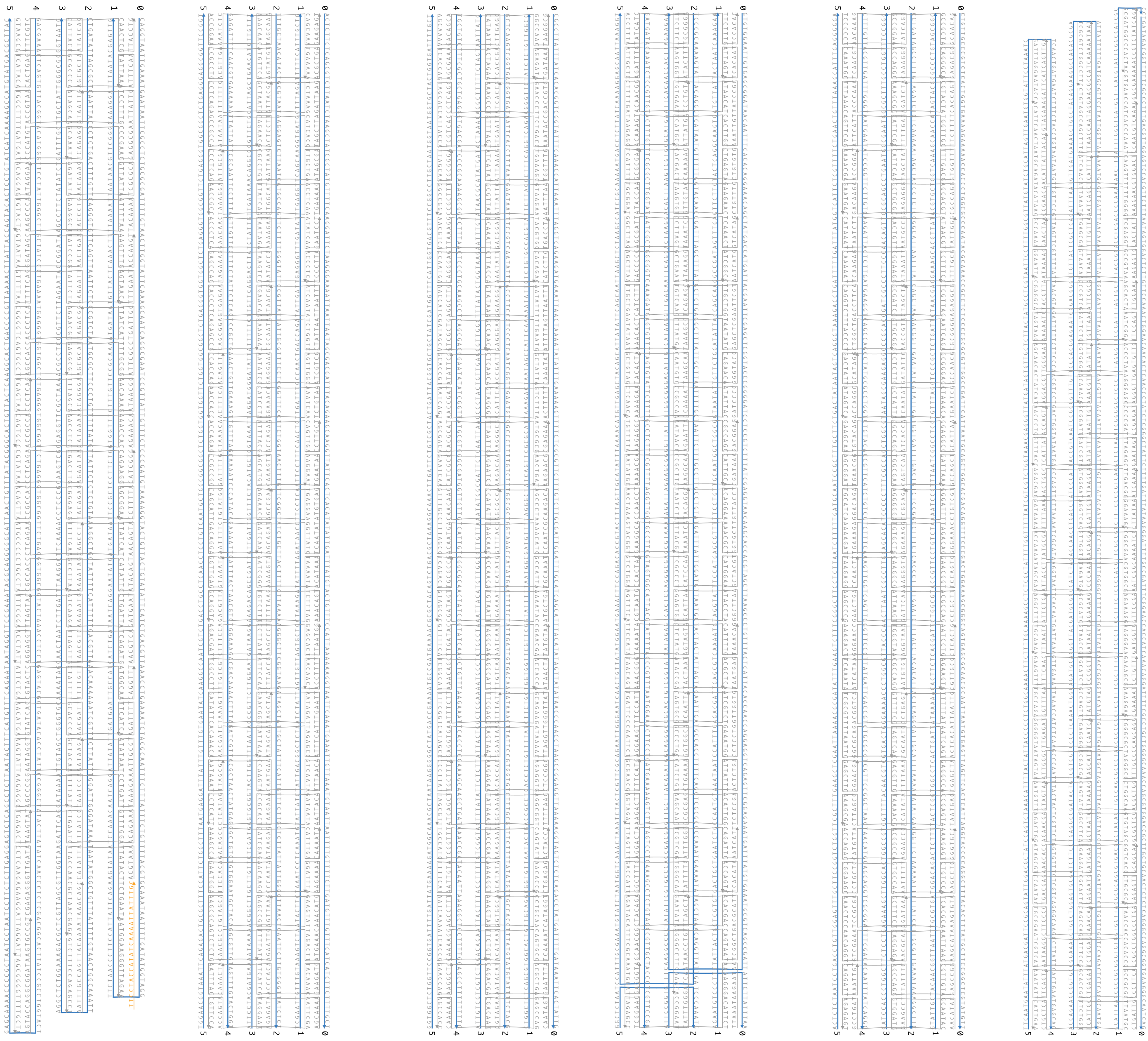

Supplementary Figure 20: Scaffold / staple layout of the 6-helix bundle object. Generated with caDNAno v0.2. Object-label is colored orange.

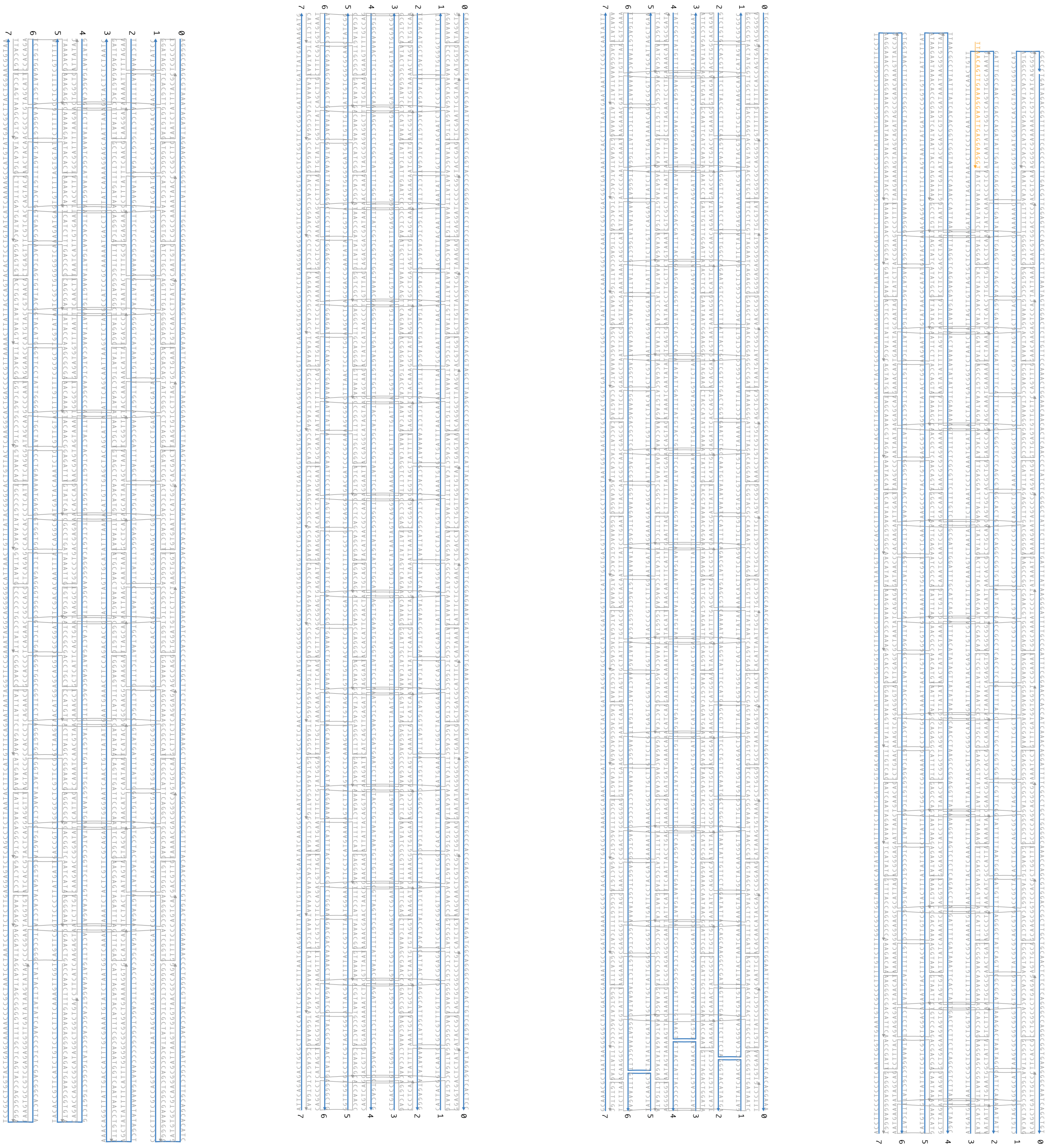

Supplementary Figure 21: Scaffold / staple layout of the 8-helix bundle object. Generated with caDNAno v0.2. Object-label is colored orange.

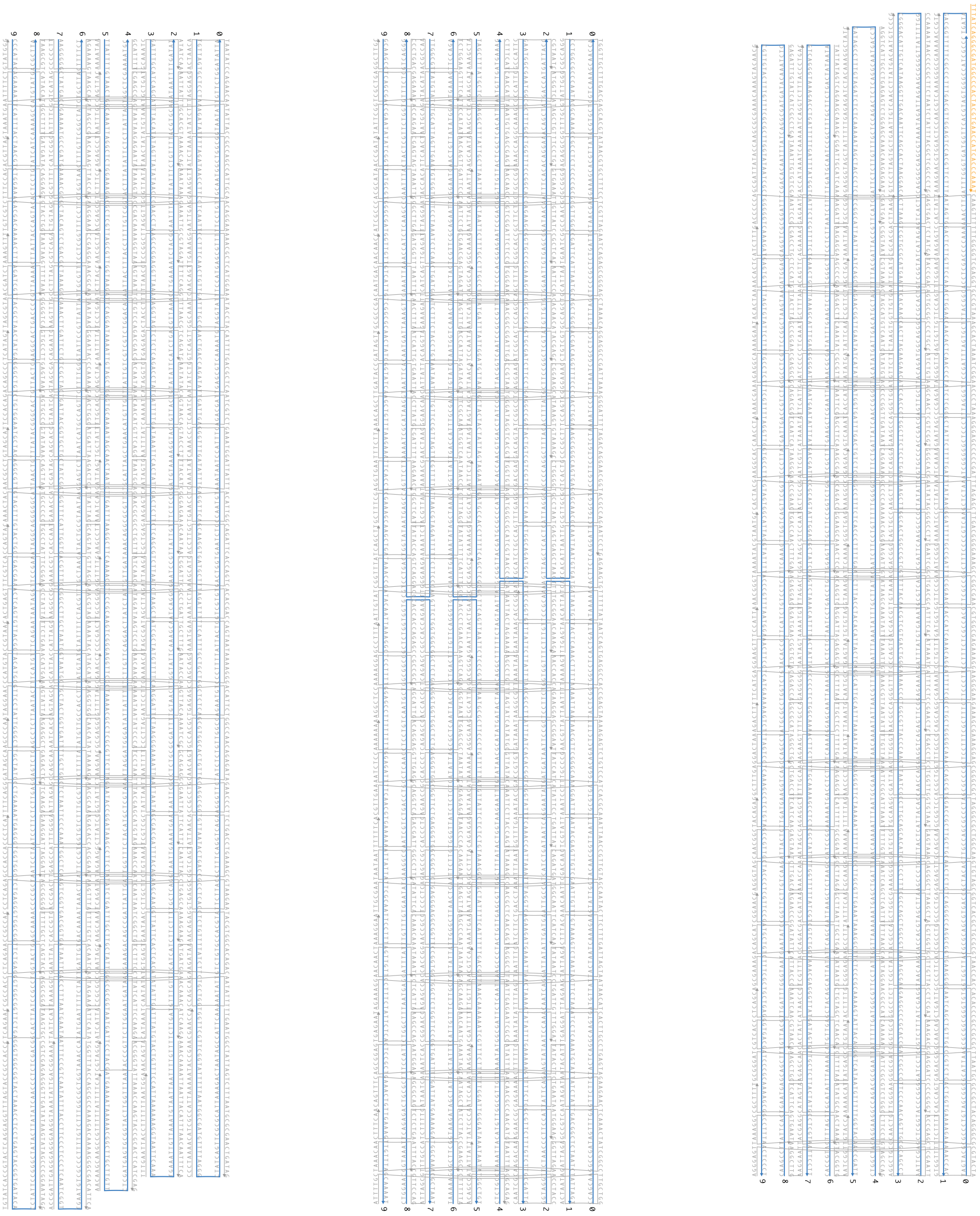

Supplementary Figure 22: Scaffold / staple layout of the 10-helix bundle object. Generated with caDNAno v0.2. Object-label is colored orange.

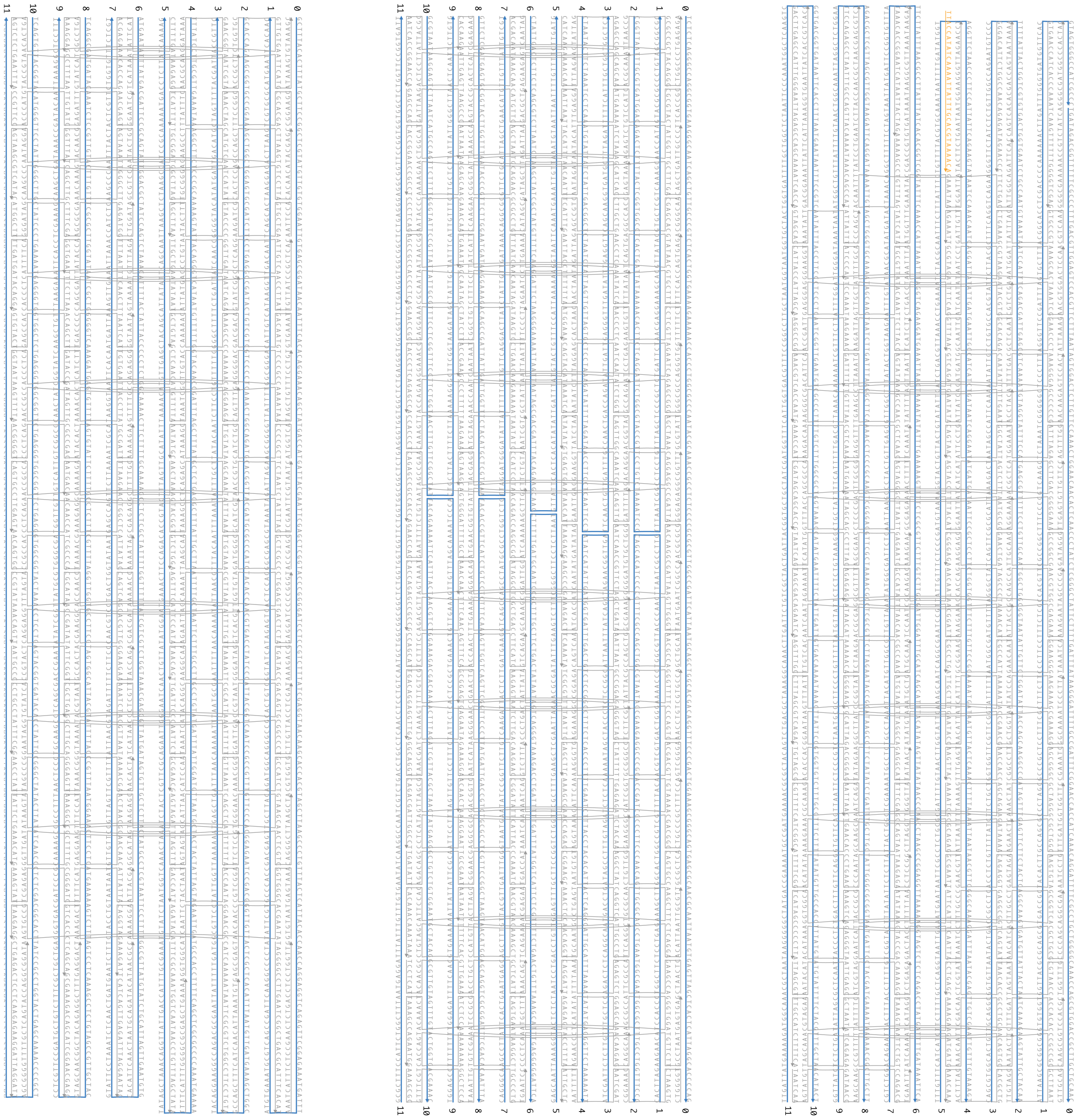

Supplementary Figure 23: Scaffold / staple layout for the 12-helix bundle structure. Generated with caDNAno v0.2. Object-label is colored orange.

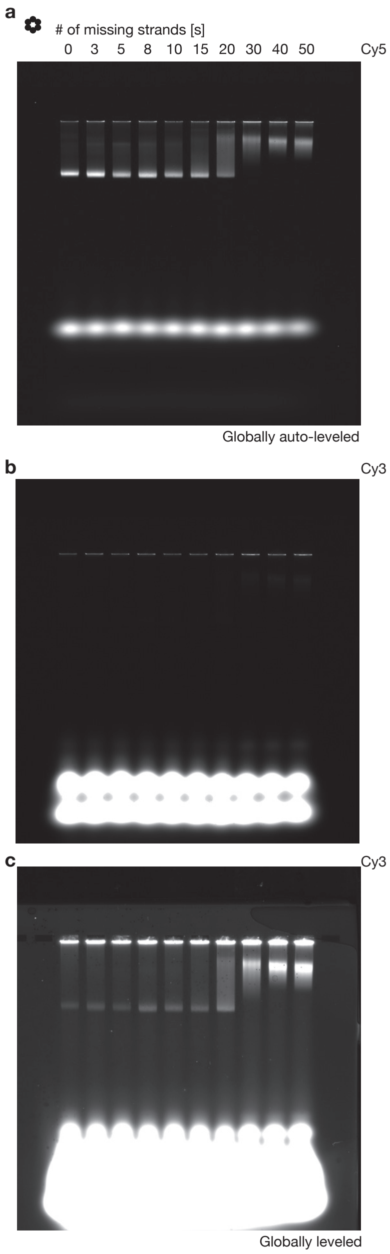

Supplementary Figure 24: Agarose-gel of six-helix bundle object. Cy5 and Cy3-channel (two different brightness-adjustments) are shown.

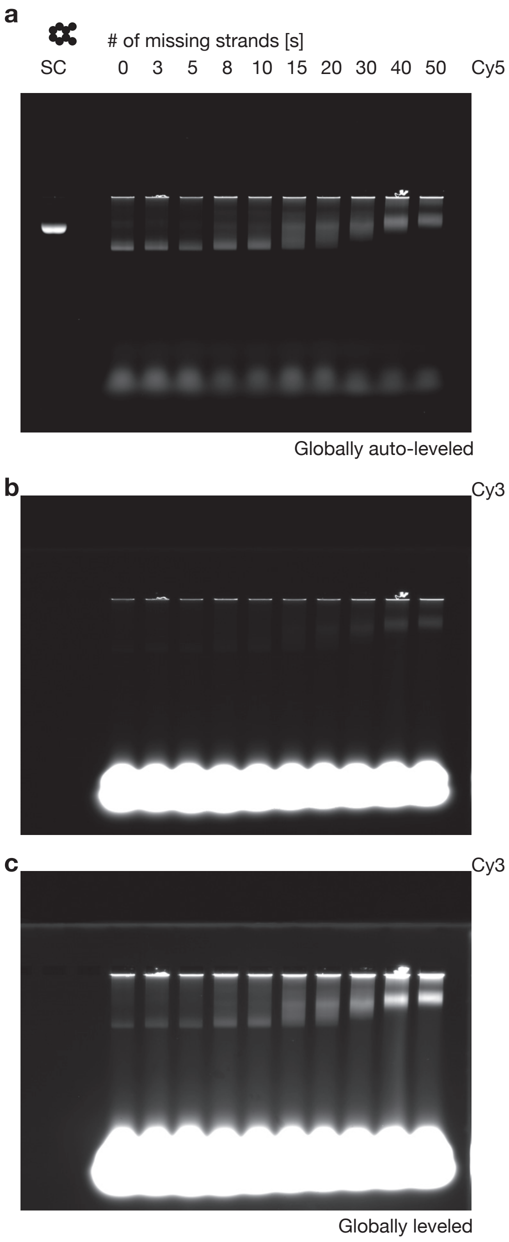

Supplementary Figure 25: Agarose-gel of eight-helix bundle object. Cy5 and Cy3-channel (two different brightness-adjustments) are shown.

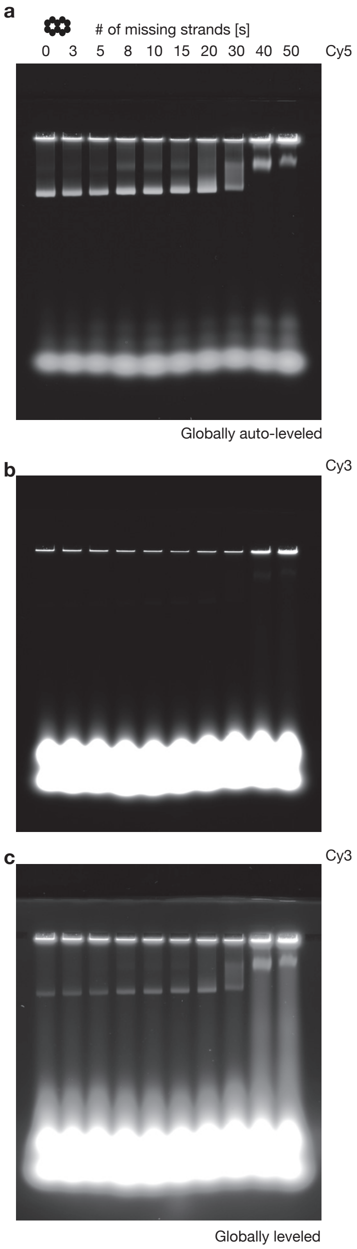

Supplementary Figure 26: Agarose-gel of ten-helix bundle object. Cy5 and Cy3-channel (two different brightness-adjustments) are shown.

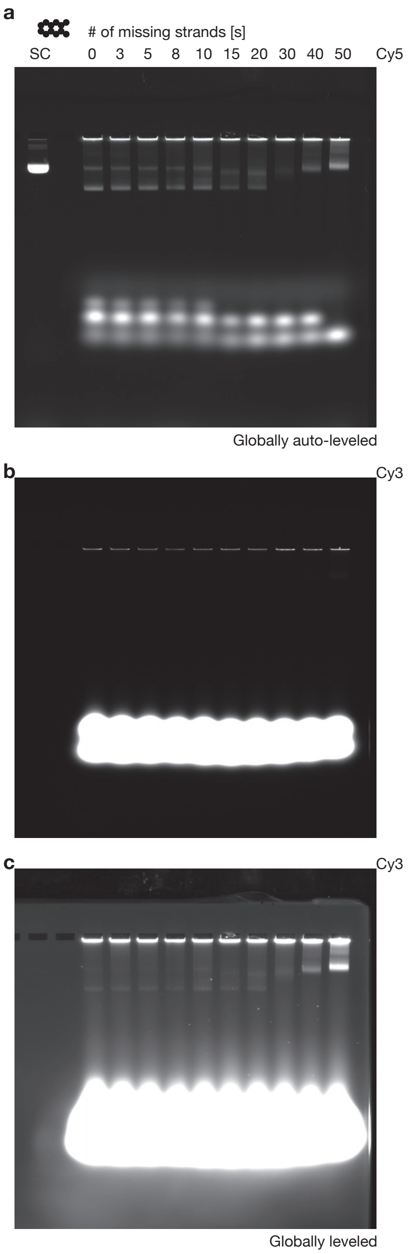

Supplementary Figure 27: Agarose-gel of twelve-helix bundle object. Cy5 and Cy3-channel (two different brightness-adjustments) are shown.

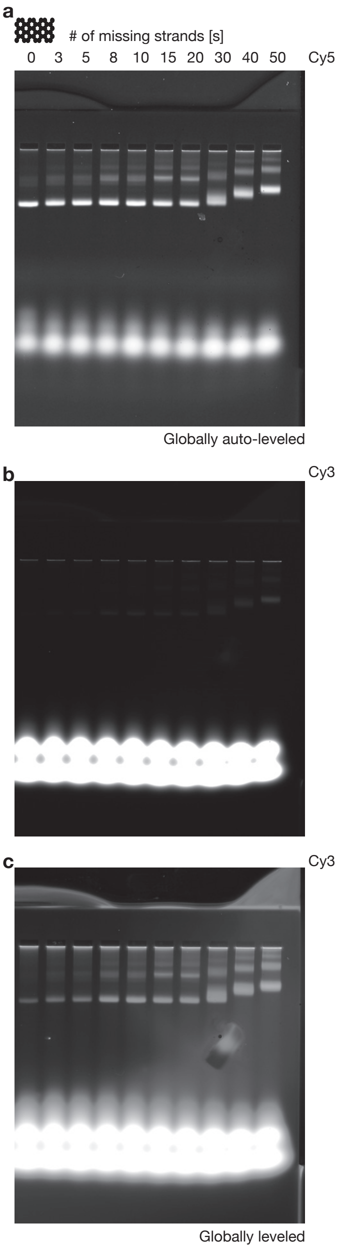

Supplementary Figure 28: Agarose-gel of 42-helix bundle object. Cy5 and Cy3-channel (two different brightness-adjustments) are shown.

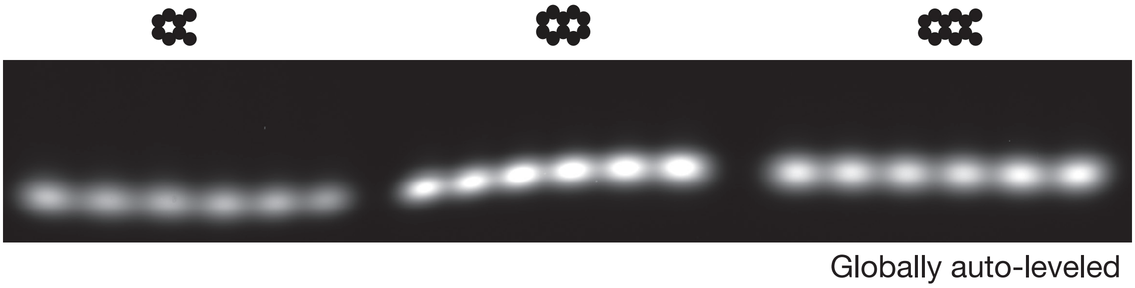

Supplementary Figure 29: Exemplary comparison of the fluorescence-intensities of different object-labels. 1 pmol of object-label DNA oligonucleotides for the 8,10 and 12 helix-bundle objects were electrophoresed in sextuplicates. Varying age and sequences might account for the intensity-differences.

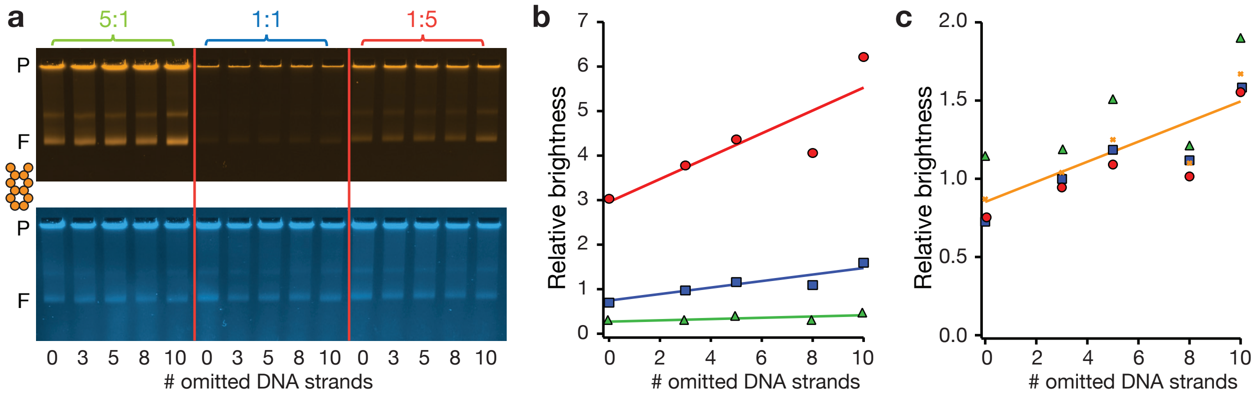

Supplementary Figure 30: Effect of different object label incorporation efficiencies on the relative brightness of bands in the defect channel vs object label channel. Three groups of pseudo-defective variants of a 10-helix-bundle were self-assembled. In each group, the fluorescently object-label strand was added together with a non-labelled copy of the same strand in different stoichiometries. (a) Upper gel image: object label channel. Lower gel image: same gel scanned in the defect label emission channel. (b) Relative brigthnesses. (c) Rescaled relative band brightness by using the stoichiometry information.

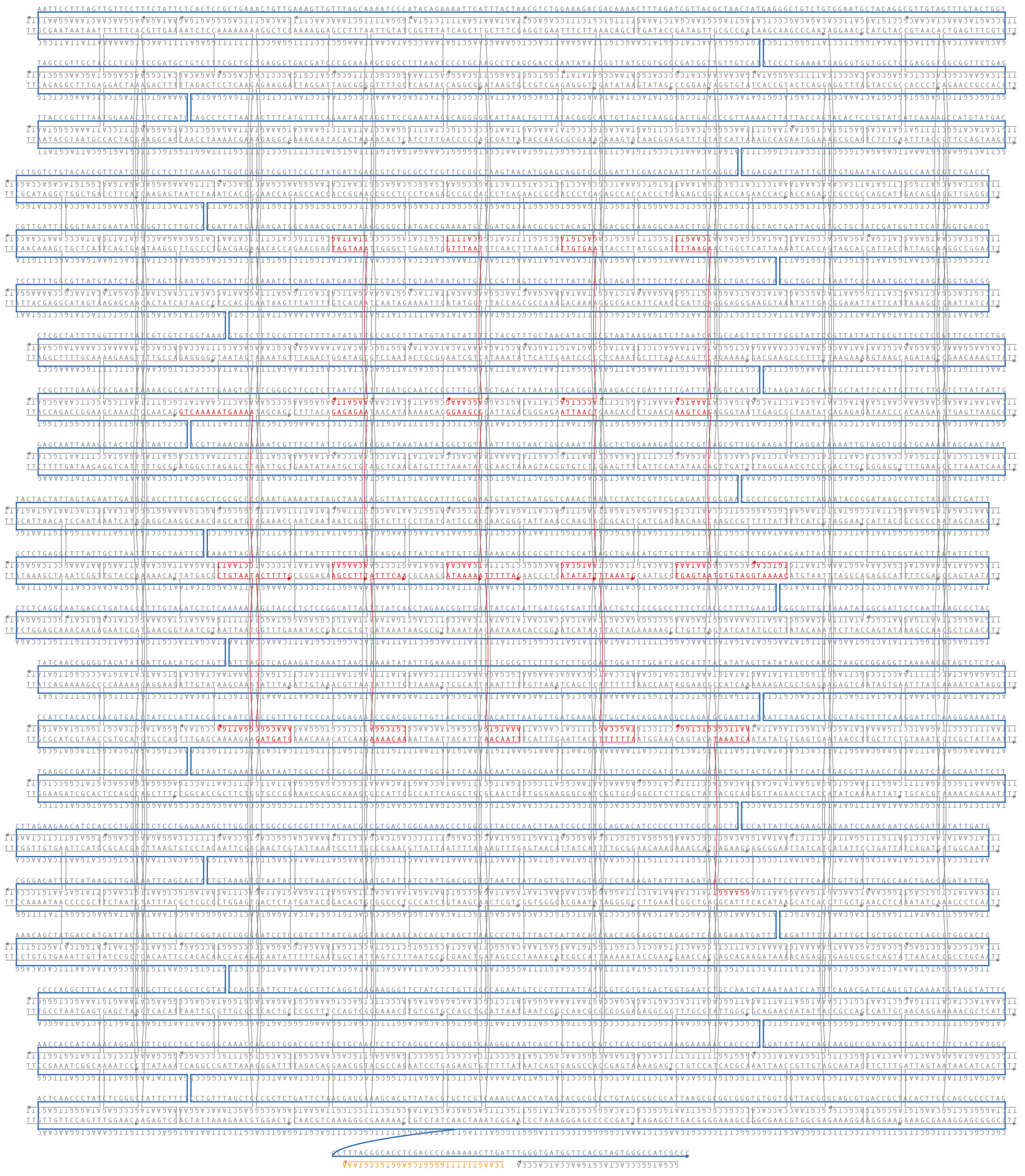

Supplementary Figure 31: Scaffold / staple layout of the 42-helix bundle (“internal defects”-variant). Generated with caDNAno v0.2. Object-label is colored orange. Omitted oligonucleotides are depicted in red.

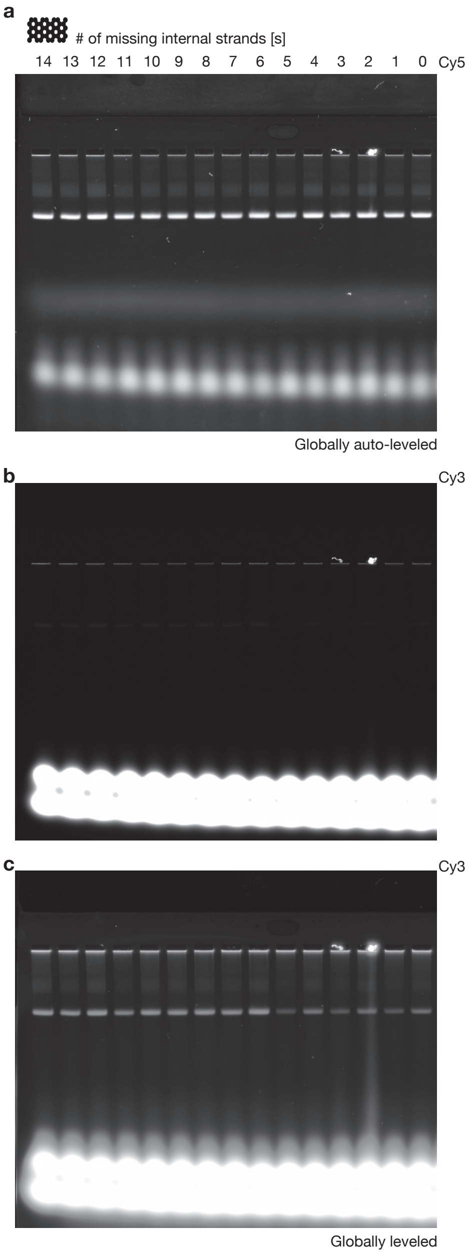

Supplementary Figure 32: Agarose-gel of 42-helix bundle (“internal-defects”-variants). Cy5 and Cy3-channel (two different brightness-adjustments) are shown.

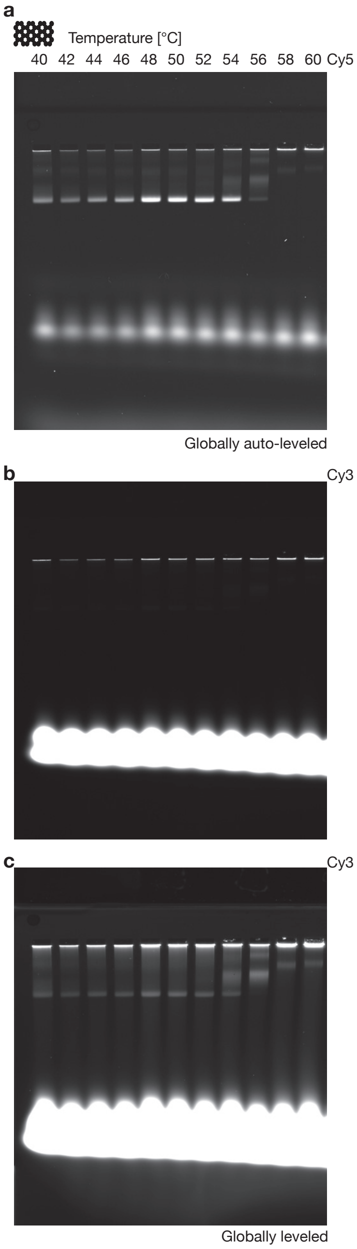

Supplementary Figure 33: Agarose-gel of 42-helix-bundle. Samples were incubated for 2h at the indicated temperatures and then electrophoresed. Cy5 and Cy3-channel (two different brightness-adjustments) are shown.

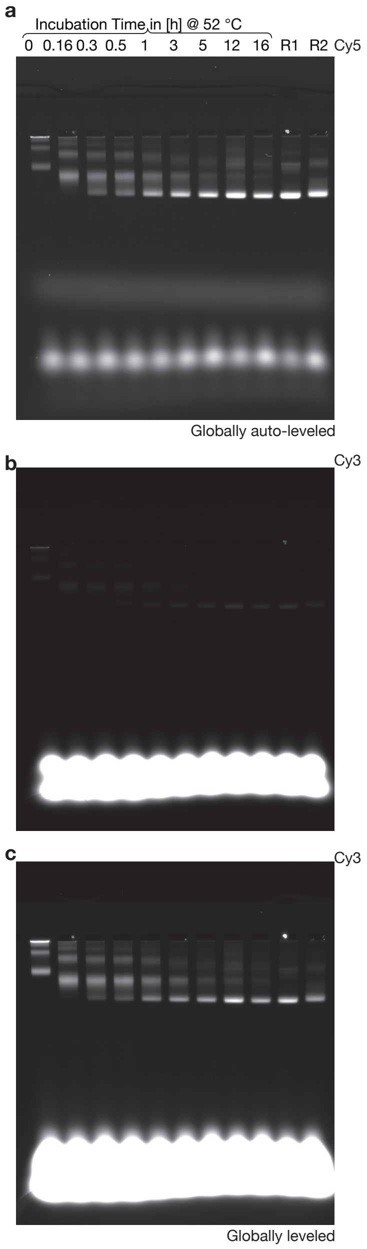

Supplementary Figure 34: Agarose-gel of 42-helix-bundle. Samples were folded at 52°C for different amounts of time. Cy5 and Cy3-channel (two different brightness-adjustments) are shown.

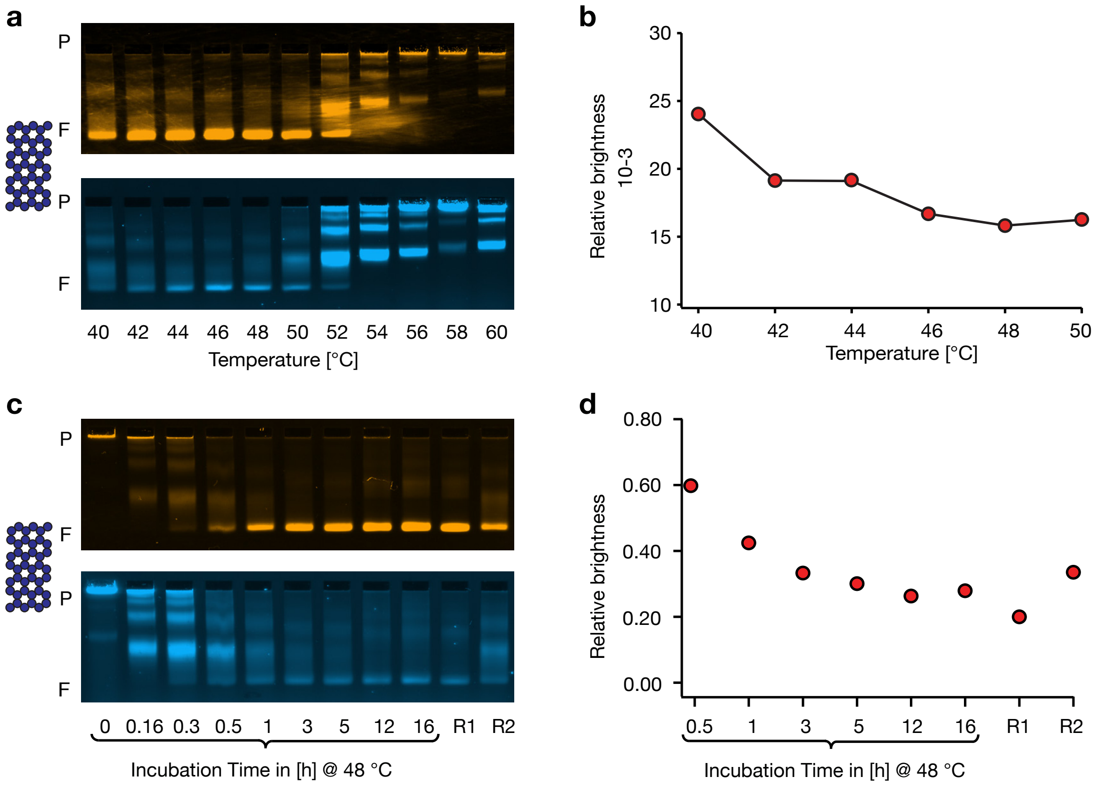

Supplementary Figure 35: As in Supplementary Figure 3 in the main manuscript, but experiments were carried out with a another version of the 42-helix-bundle object that differed in its topology of chain connectivity. In contrast to the version examined in Supplementary Figure 3, this version folds best at 48°C (b). Also, the difference in folding quality as judged by the relative defect labeling intensity for constant temperature folding versus thermal ramping (R1) is minor.

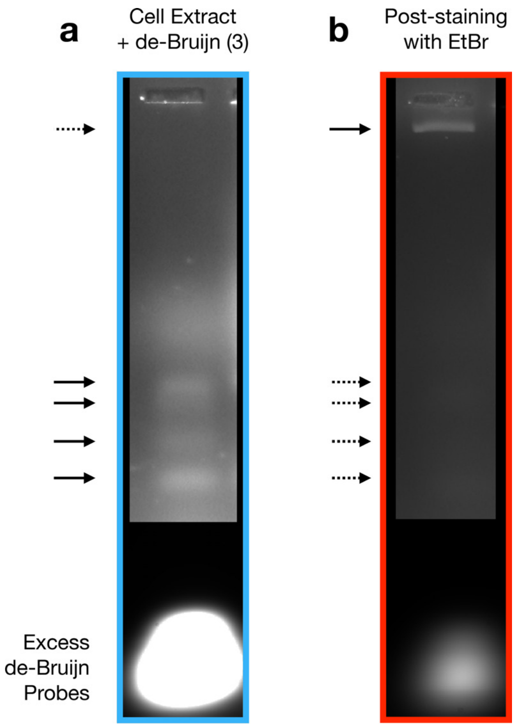

Supplementary Figure 36: Labeling E.Coli cell extracts with the de-Bruijn probe. Cells were lysed mechanically, the extract was incubated with the de-Bruijn probe of order three, and then electrophoresed on an agarose gel. The gel was first laser-scanned in the de-Bruijn defect label channel, then soaked in an Ethidium Bromide solution, and scanned again.

Sequence	Length
AGGAACAACTAAAGGAATTTT	21
AGTTAGCGTAACGATCTAAAGTGATACCCCCACGCGTATAGC	42
CATTCCACAGACAGCCCTCATCAAGCAAGCCCAAT	35
CCTGTAGCATGTACGAGCCACGTACCGCGATACAGTCTCTGA	42
TTACCAGTACAAACTACAACG	21
TTGCGAATAATAATTAGCAACGGCTATT	28
AATCTCCAAGACAGTTAGACTCCTCAAGTCATAATGACGAGA	42
AAAAAAAGGAATACTCCACGGAATAAGTTTATTTTTCAGTTG	42
AAAAGGAGATCGTCTTAGGATTGAAACAAAAGAATACACTAA	42
GCCTTTAAGATTCAGTCACAAAACATATAAAAGAA	35
CGGTTTAAGTTAAATCAGTACCTATTTCATCTTTGACCCCCA	42
TCAGCTTACAACATATATGGTAAATACATACATAA	35
AGGTGAACGGTCGCCCGTCGATAAACAGTACCAAGCGCGAAA	42
TTTCTTATTAATAAGACAAAACAGTATGTTAGCAA	35
GATAGTTCGTTGGGCGATTGATGATTAAGACTCCTGCTTTAA	42
ACGAGGGTTTTTCAGCAGATA	21
TTCTCAGAACCGCCACCCTCACGTAACAACTTGAGTAAAGGTCGAGGAA	49
TTCAGAGGCTTTGATTAAACGGGTAATT	28
TCACCGTTAAGTTTAAGCCAGAATGGAAACAAATAACCAGAG	42
ACTCAGGCCGTAATATCACCATAGCTATCTTACGA	35
GTTTCCAGGACTAAGATATTCTCAGTGAAATTCGAAAGAAGTGCTATCA	49
TGAAAGTATTAAGAGGCTGCACAACCTAAAACGAATGACCAA	42
GGAACCTATTATTCTAGCGGGTTATTAGTAGTAAAAAGATTA	42
TTAATGCCCCCTGCCAGGCGGGCATTTTGTTTAATAGCAAAG	42
TAACAGTGCCCGTAGAGGGTTGACTGTATTGTGAAACCCTGA	42
CCTTGAGCAACGGAGATTTGTATCATTATAACGGGGTCAGTGCCGGAAT	49
TTGTCATACATGGCTTTTGATCACCCTC	28
TTAATACGTAATGCCACTACGAGATGAACCTTCAT	35
ATTTACCGATTGGCGACAGGATTTGCACACAAGAAAAAGTACTTTCGAG	49
TCAATCAACCGGAAAATCCAAGAGAGAAACAAGAAAGCCTTT	42
GCCGGAAGCCGCCAAACAGCCGGAAGCGTCAACAAATAAAAA	42
CGACCTGACCCTCATAATTTGATTAACTAATGCAGATATATT	42
AATCCTCATCGCCTGATAAATAGAGCCGAACGAGC	35
TTAGGTCAGACGTTCCAGTAAGCTT	25
TTCGCATAGGCAAATCAACGTT	22
ATCACCGTTTGTTTAACGTCAATGGCTTAGAGCTTGGGCGCG	42
CAGCATTCTTGATATTCACAAAGCGCAGGAGTGTACTGGTAA	42
TCAAGTTTTTAAGATAAATCAACAGTTCTGAGAAACTGTTTA	42
ATGAAACATTAGCACAAGAAAAGATAGC	28
TTACGTCACCAAGAACCGCCACCTT	25
TTTAACAAAGCCCAAAAGGATT	22

TGCTCATATTACCCTGGCTGACGGTGTACAGACCAGGTT	39
GAACGAGCGTTTGCAGCCACCTAAGGGAACCGAACAGAGGCA	42
TTGGGCTGGTAGAAATTGTATGGGATTTTGCTAAACAACTTT	42
TGAGATGCGGTCATCCTCAGACGAGGCGCAGACGGAACACTC	42
TTCAACTTAACGGAGCTTTCGTAGTAAATGAATTTTCTGTAT	42
TTAATCAGCGCGTTAACCGCCCTCCATGTTACTTAGCGATTA	42
TTACCTTATCTACGAACAGCTTTTTGTCGTCTTTCCAGACGT	42
ATGCGATTGCCTTTCACCCTCTGTGTCGAAATCCGCAAAGTA	42
CACATTCTTGCCCTCATGACAAGAACCGAGACTTTCATCGGA	42
GAGCCAGATATTGAGAATACCCAAAAGAGACGAAGCCCTTTT	42
TTCCGTCACCGCTGAGTTTCGTCTT	25
TTATTACGAGGCATAGTACATAACG	25
AGAGCAATAAAAACCAAAATAGCGAGTT	28
ACTGGCAGGGAGGGAAGGTAACATACCAGTCAGGAGCGCCGA	42
AATAACGCGGAAATTATTCATCCATTTGCATTACCCATCGAT	42
ACGCAATAGTAAGCCAATGAAATAACCCCCAGCTAGGGAGGT	42
TTCCAGAAGGAAACGAATTATCATT	25
TTAGGCTTTTGCAAGCTTCAAAGCGATT	28
TTAGACTAGAGGAACTTTACAATAAGAAGAATATAATGCTGTGTTTAGC	49
GGATAGCTTAATGCACCGTGTAGTTAATTTCATCTATTGTAA	42
ACTGCGGCGGATTGAAAACAGATATTATCATGTTTTAAATATTTCGCAA	49
AATCGTCTCAACCGTAAATAAAAACTTTTTCAAATTTAAAAT	42
TTCATTGCTATTATCGGGAGACCAGTTAAAGTACGGTGTCTGTAGTTTG	49
AATCCCCAAATCACATCATAACGCAAGACAAAGAATGTTAAA	42
GCGTTTTATAAGGCAACTAATCGTTGAAAATAGAA	35
AAATATCGTCAAAAATGAAAACCTAATTCTGTAATGGATTGA	42
AGACTTCTAATAGTATTTTTGAATTAATGGTTTGAAATACCG	42
GCCCGAAAACACCAAGATTTAAGGCTCCCAACAGTTTCAGCGGAGTGAG	49
AAAATCACTGAACAGTCTTTCCATTCCATATAACAGTTGATT	42
TTCAATAATAAGAGAGGCCGGAATT	25
TTACCAGACCGGAACTTTAATTGCTCTT	28
AAGTCAGCAATAAATGAGTAAGTCTGGCAGGAACGCGAACTCGAACCAC	49
AGGGTAAATGACCAACTGGCTCATTAAACAGTAGCAACCACC	42
TTGAGCGATCTTACTTAGCGAACCTCCCCTAAGAATTCATCG	42
AGAGTACGCAAACTATTAAGCTGTACCA	28
GGATTAGCAAGAGTAATCTAACTTTGAAAGAGGACAAGGCACTGAGGAA	49
CAACGCTCCACCAGGACAGAAAGGTGTAGACAACAACCATCG	42
ATCCTGACTAATATGTAATTCATGTAATATAGCTTAGATTAT	42
CAATTTTCCGCCGCAGCAGCAAGGTTTACACCCTCATTTTCAGGGATAT	49
TTATTAGTTGCTATGGTTGAGGCTT	25
TTCTTTTGATAAGAGGTCATTCATCAATAATCATA	35
AATTGCTACGATTTGAACCAGCATCTTTAGAAGGAACCCTCAGCAGCGA	49

AGCTCAATTATCCCCCGCCTCAGCCCCCGTTTTGCGGCCGCTTTTGCGG	49
GCAACTACAAAATACCCTCAGTTCATCGATAAGTGTGAGGCTTGCAGGG	49
GAAGTTTCAGAGCCGAGCCACAGCGTCAGATATAAATAACCGATATATT	49
TTTGAAGATAGAAGGCGCCCAAGATTTTGTAAATCGGTCAGTAATATCA	49
TTAAATCAGATCCTTAAATCAAGTT	25
TTGCATTAACACCTCAGAGCTT	22
AGCATGTATTTCAATTACCTGCGGCACCGCTTCTGTGCCAAG	42
AATCGGCTCGCGCACATCAAGTTGAGGAGACAGTG	35
ATTCCAATTTGAATTACATTTAGCCGTCGTAAGCA	35
CCAAGTAATAACGGAATTACCCACTAACACGAATA	35
TTTTATTCGCGAGGCGTTCCCAATTCTGAACAAGCCCTTTTA	42
AGGTAAACAGAGAGATAGCAAGTAGCACGGAATTAAGGAACC	42
TTAGAGAATATTTGAGTTAAGCCTT	25
TTATAAAGCTAGCCTGAGAGTT	22
AATCGGTAATAAAGTCCAATATCTACTAATAGTAGTATT	39
GAGGCATCGACAAACATTACCGCTTATCCGGTATTGACTTGC	42
TACAAAGTTTGCCAACGACGACACTATCATAACCC	35
AGAGATCTATGACCTAAGAATTAGCAAACCAACAG	35
GGTAGCTAAAATGTACGCAAAGACACGTTTACCAGGAGGGGG	42
CGGAGAGCGGGAGAAAATAATAATCAATTATATTTTCATTTG	42
TGATAAAGTCCAATAGGTGGCTCAATAGAAAATTCTATTACA	42
TTCTAGCCGCAAGGTAGATAACCTTATCATGGTCAATAACCT	42
ATGATATATAAATAACGTAGATTACCAGCGCCAAAAACGAAC	42
CATCAATAACCCTCAACGCGCTATTAAAACCATTAGATACAT	42
GACAGTCCTCAAATTATTACGGGGCGACATTCAACAAGAAAA	42
GGCCGGACAATGCCCAACATGCATCGAGCGAACGAGTAGATT	42
ACAACGCATACAAATTGGGTTATATAACAAAAGACGCTGAGA	42
AATTGAGAAGCCAACTTTTTA	21
TTGTAGGGCTTCGAACAAAGTTATT	25
TTTCTGGAGCAAACAAGAGGTCATT	25
GAATCGAATATGTACCCCGGTTGATATT	28
TCTGACCTAAATAACTAGCATATTGTATCCGTAAT	35
AAATGCTGATGCAAAACCAATCTTCCTGATGGAAACATCGGG	42
TATATGTGTATCATATGCGTTCAGATTCAAAAGGGAGAAAAC	42
ACCTCCGATAGTGATTGAAAATGCTTCTCAGGTTTAATTATT	42
TTCTGAGAGACTACCGCTCAACATT	25
TTATCAGAAAAGCCTGGTGTAGATGGTT	28
AAGCAAACCCGGGTACCACAGACAATATTTTTGAAAAAGACG	42
GTCACGTCCAAAAACGTAATC	21
TGGGAACAAACAAAGAGGCGAAAACCAGGCAAAGCACGTTGT	42
TTCTCCGACGTTAACCTCGATTGGCTATTGACCTGAAAGCGTCCGCTTT	49
AACAACCAATTAATACCAAGTGCCATTCAGGCTGCGCCAGGG	42

AGCGAGTTCGCATTTAAGCTACGAACTGCCAACAGAGATAGATGTCGTG	49
CAACATTTCATTTGATTCGCCTTGGGAAGGGCGATAAGGCGA	42
CTTTCATTCAGCTCGTGTAATTCGCCATGTAATAAAAGGGACGAATCGG	49
AGAATCCATTTATCAAAATCATAGGTTT	28
TTTAATTTTCCCTTCCAGTAATATT	25
TTGCGCATCGTAACGTATCGGCCTCATT	28
TTTGAGCAAAAGAAACTTTACCTGGAGTGGTTTGCGTTGCGCTCACTGC	49
CAGTACATATCTAACGGCTGATTTTCTTTTTGCGTATTGGGC	42
ATATATGATACAGTGGTTAGAACCTACCACTTCTG	35
GACGACACGTGCATGTCAACCTCTAATCATCCTGTGAGCTAA	42
AGGGGACCAGGCAAGGCAACGAGCTGAAAAGGTGGTTTGCGG	42
GATGAATTGAGTGATTGAAAGGCATCACCTGGTAATATCCCA	42
TTAAGAAATTGCGTATAGCAAGCTT	25
TTGGAAGATCGCACTCCAGCCGCCAGGG	28
GTGCCGGATTATTCAGAAACCATCCCATTAGCAGC	35
GCCATTCTACAAAATGTCTTTGTCCTGATAACATACATCAAA	42
GCAACTGTGATTGCGAACGGGCTGTTTACATTAGAAGTCAGA	42
CGGTGCGAGAAACACCGCACTTTCAGCTGAACACCGGTCTTT	42
GGCCTCTTCGCTATTACGCAGAACAGTAAAGCCGTACGACGA	42
TGCACGTAATCCTGTCAGATG	21
TTCATCAATATAAAACAGAAATATT	25
TTTGGTTGTGAAATGTCCCGTT	22
CGCACGAGCCCGAGAAATCCC	21
AATTCGAGAGCTAAACAGGAAACTATTAAAGAACGTGGACTC	42
TCCTTTGTCCTCGTGAACGGTACGCCAGGAGAGAGCGGCCCT	42
ATTTTAAACGAGCAAGAAGTGTTTTTATTGCCCTTACTCGTC	42
TTATCATGCGCGTAGAGGCCACCGAGTAGTGAGACTAGGGGC	42
TTATCATGCGTAACCGTTGTAGCAATACGGCCTTGCTTGCTG	42
CATATTCTCAACAGATAACCTCATAGCGTTAGGCAATATTTATAAGAAA	49
CTGATTAATTGTTTGGATTATATATCAAAACGTCATAGGAAT	42
GGAAGGTTAAATCAAATTCGCTGTGTAGGTAAAACTGTCCAG	42
GAATTGAAGCGGAAAATAATGGACAGCTGGCGAAAAAACCAC	42
TTTCAATATCTGTCGCTATTAATTT	25
TTCCAAAATAATAGCTGTTTTT	22
CCCCGCTTTATGACATTCATGTGGATGTTCTTCTAAGTT	39
GCTCGCCAATAGTGCTGAATTCTGCCAGGGGATAGAAAACAT	42
TGATACCTTTAGAATATTAAAAAAACGACGGCCAG	35
CTTGTTATATTTTGATATTTTGATAAATAAGGCGT	35
CGTGGTGGCCATCTAATAGATGTTATTATTTTCCCAGTCACG	42
CAGGGCTAAATTTTCGCGAGAGAATAAACACCGGA	35
GAGTAAAGGTGGGCAACTAATAGTAACATTAAGTTGGGTAAC	42
CTGGTTGATTTTTTATCCAATTTACTAGAAAAAGC	35

TGACCTCCTTGAATAATATCTAACAAAGGGGGGATGTGCTGC	42
AAAAATCACAGAGGAATGGATTATTTACGCAGAACAATATTACCGCCAG	49
GCAAATGAGAGTCAGCTTAGGTTCTTACCAGTATAAATCGCC	42
TTCAGTGCCACGCTGAGA	18
TTCCTGTGTGAAATTGTTATGGTCA	25
ATCCGCTTAAAGTGTAAAGCCTGGGGTT	28
TTAACACCGCCTGCAATT	18
AAGAATACGTGGATACGAGCCTAATTGCCCCAGCA	35
GATTCACCAGTCACGAGGCGGTTCACCAAAAGAGT	35
ATTGGCACAGCAGAAGATAAATAAAATCATTTCTCCCATCAA	42
CGTCTGATGAGGCGGTCAGTAGCCAGCAAACCTCATGGCAAA	42
GCTCAATCCATTGCAACTATCTTCTTTGAGCGGTCAGGGAAG	42
TTGAAATACCTACATTTTGAC	21
TTTGCCTAATGAGTTTGATGGTGGTTTT	28
CTCACATGGAAGCACACAATTCCACACAACCGAGC	35
AAGCGGTTAGACAGTAGAATCCAACGTCAAAGGGCGAAAAAC	42
TTGCAGCCCAGTCGGGAAACCACCCTTCTAGTCTTTAATGCG	42
TGGCCCTAATCCTGCGTATAACGTCTATCAACTAAATCGGAA	42
CACCGCCCCAGCTGCATTAATATTCTGGATAGCCCTAAAACA	42
AGCTGATAATCAGTCTATGGTCCCTAAAGGGAGCCCCCGATT	42
GGGCAACCCAACGCGCGGGGAACGACCATAAAAATACCGAAC	42
GAACTCAAACAGGAAAAACGCTCATGTT	28
TTGCCTGAGTAGAAAACCCTCAATT	25
TTCCGAAATCGGCAATAGGGTTGAGTTT	28
TTATAAAGGCGAAATATTTACTCGAATTCAGGAAGGTCAATCTGAACGGTAATCGT	56
TCAGGCCGATTAAAGGGATTTCCACGCTGACTCTAGAGGATCTATTTAA	49
AATTAACCACCACAGGCGAACGTGGCGAGAAAGGAACGCTGC	42
AGAGCGGCAACTCGGTATTAGGATGATGAAACGGCACTTTTG	42
CGTGCTTCCCGAACAATACATAAAACAACGTCGGAATTTCAA	42
TGCTTTGAAGTTTGAGATTAGAACAATTAAATGTGTTTTTAG	42
CTACAGGTTTGCGGTTAGGAGTTTTTTATAGCCAGTTAAATG	42
TGCGCCGTAGAGCTTGACGGGGAAAGCCCCCGCCGCAGAAGG	42
CGCTTAACTGTCCATCACGCAGGGTGGTCGCATTTCACATAA	42
CAAGTGTATTAGTAATAACATCACTTTT	28
TTCTAGGGCGCTGGATGGCAATTTT	25
TTGTTGTTCCAGTTTGGAACA	21
AGAGTCCAAGAATACTTAAGTGTCCTACCTTTCTCAGGAGAAAGCTTTC	49
AAAGCGAAAGGAGCGGGCGTT	21
GCGATGGCCCACTACGTGAACCATCACCCA	30
object-label	
Cy5-TCAAGTTTTTTGGGGTCGAGGTGCCGTAAA	30

Supplementary Table 1: list of oligonucleotide-sequences of the 42 helix-bundle

sequence	length	defect variant:
TGATAAAGTCCAATAGGTGGCTCAATAGAAAATTCTATTACA	42	3
TTCTAGCCGCAAGGTAGATAACCTTATCATGGTCAATAACCT	42	3
ATGATATATAAATAACGTAGATTACCAGCGCCAAAAACGAAC	42	3
GGTAGCTAAAATGTACGCAAAGACACGTTTACCAGGAGGGGG	42	5
CCTGTAGCATGTACGAGCCACGTACCGCGATACAGTCTCTGA	42	5
GAGGCATCGACAAACATTACCGCTTATCCGGTATTGACTTGC	42	8
TTATCATGCGTAACCGTTGTAGCAATACGGCCTTGCTTGCTG	42	8
TTATCATGCGCGTAGAGGCCACCGAGTAGTGAGACTAGGGGC	42	8
TTTTATTCGCGAGGCGTTCCCAATTCTGAACAAGCCCTTTTA	42	10
AGGTAAACAGAGAGATAGCAAGTAGCACGGAATTAAGGAACC	42	10
ACTGGCAGGGAGGGAAGGTAACATACCAGTCAGGAGCGCCGA	42	15
AGCATGTATTTCAATTACCTGCGGCACCGCTTCTGTGCCAAG	42	15
ATGCGATTGCCTTTCACCCTCTGTGTCGAAATCCGCAAAGTA	42	15
TTACCTTATCTACGAACAGCTTTTTGTCGTCTTTCCAGACGT	42	15
CGGAGAGCGGGAGAAAATAATAATCAATTATATTTTCATTTG	42	15
ATCCTGACTAATATGTAATTCATGTAATATAGCTTAGATTAT	42	20
CGGTTTAAGTTAAATCAGTACCTATTTCATCTTTGACCCCCA	42	20
AAAAGGAGATCGTCTTAGGATTGAAACAAAAGAATACACTAA	42	20
AAAAAAAGGAATACTCCACGGAATAAGTTTATTTTTCAGTTG	42	20
AATCTCCAAGACAGTTAGACTCCTCAAGTCATAATGACGAGA	42	20
TGAGATGCGGTCATCCTCAGACGAGGCGCAGACGGAACACTC	42	30
AAATATCGTCAAAAATGAAAACCTAATTCTGTAATGGATTGA	42	30
AGACTTCTAATAGTATTTTTGAATTAATGGTTTGAAATACCG	42	30
TTCAACTTAACGGAGCTTTCGTAGTAAATGAATTTTCTGTAT	42	30
AAAATCACTGAACAGTCTTTCCATTCCATATAACAGTTGATT	42	30
TTAATCAGCGCGTTAACCGCCCTCCATGTTACTTAGCGATTA	42	30
AGGGTAAATGACCAACTGGCTCATTAAACAGTAGCAACCACC	42	30
TTGAGCGATCTTACTTAGCGAACCTCCCCTAAGAATTCATCG	42	30
AGGTGAACGGTCGCCCGTCGATAAACAGTACCAAGCGCGAAA	42	30
CAACGCTCCACCAGGACAGAAAGGTGTAGACAACAACCATCG	42	30
GAGCCAGATATTGAGAATACCCAAAAGAGACGAAGCCCTTTT	42	40
GATAGTTCGTTGGGCGATTGATGATTAAGACTCCTGCTTTAA	42	40
AATAACGCGGAAATTATTCATCCATTTGCATTACCCATCGAT	42	40
ACGCAATAGTAAGCCAATGAAATAACCCCCAGCTAGGGAGGT	42	40
CAGCATTCTTGATATTCACAAAGCGCAGGAGTGTACTGGTAA	42	40
GGATAGCTTAATGCACCGTGTAGTTAATTTCATCTATTGTAA	42	40
TCAAGTTTTTAAGATAAATCAACAGTTCTGAGAAACTGTTTA	42	40
AATCGTCTCAACCGTAAATAAAAACTTTTTCAAATTTAAAAT	42	40
GAACGAGCGTTTGCAGCCACCTAAGGGAACCGAACAGAGGCA	42	40
AATCCCCAAATCACATCATAACGCAAGACAAAGAATGTTAAA	42	40
TAACAGTGCCCGTAGAGGGTTGACTGTATTGTGAAACCCTGA	42	50

TTAATGCCCCCTGCCAGGCGGGCATTTTGTTTAATAGCAAAG	42	50
TGAAAGTATTAAGAGGCTGCACAACCTAAAACGAATGACCAA	42	50
TTGGGCTGGTAGAAATTGTATGGGATTTTGCTAAACAACTTT	42	50
AGTTAGCGTAACGATCTAAAGTGATACCCCCACGCGTATAGC	42	50
TCACCGTTAAGTTTAAGCCAGAATGGAAACAAATAACCAGAG	42	50
CGACCTGACCCTCATAATTTGATTAACTAATGCAGATATATT	42	50
GCCGGAAGCCGCCAAACAGCCGGAAGCGTCAACAAATAAAAA	42	50
ATCACCGTTTGTTTAACGTCAATGGCTTAGAGCTTGGGCGCG	42	50
CACATTCTTGCCCTCATGACAAGAACCGAGACTTTCATCGGA	42	50

Supplementary Table 2: list of omitted oligonucleotides of the pseudo-defect-variants of the 42-helix bundle.

Sequence	Length
GGCGAAAAACCGTCTATCAGG	21
AAGAGTCGTAACAATTAAATTTTTGTTAGCCAAAAGAACTAA	42
GAATAGCGACCGTAAATATTTAAATTGTAACCCTCACCAGTC	42
GATGGTGGTAACCGGATAATCAGAAAAGAGGCTTTATTGTGA	42
GCGGTCCGGCCTCAGGTAATCGTAAAACAATGTTTGTAGTAA	42
CTGATTGTTCTGGTTCATTGCCTGAGAGAAATATTTTCAGTG	42
CGCCAGGGCTGCGCGAGAGGGTAGCTATAAAACGAACCGGAT	42
TGAATCGGCTATTATCAATATGATATTCGACTATTGCATAGG	42
CCCGCTTATTAAGTGATTCAAAAGGGTGTAAGAGGCCAACTT	42
TAATGAGTAAAACGTCATATATTTTAAAGAGCTTCGGAACGA	42
AACATACGTGGATGGAAGCCTTTATTTCCAGGATTAAATTGT	42
CTGTTTCTAAGTGTGTTGTACCAAAAACTCATTTTGCGCGAA	42
GATCCCCCCGCCAAGCAAAATTAAGCAAGCTGTAGTACACTA	42
GGCTTAAGGAGTGAGCATTAACATCCAAGTCTGGACCACTAC	42
TCTCCGAAAGCAACGCGCGAGCTGAAAAGCGAACGTTTCATG	42
TTAAATAAGAATAAAAAAGGGACATTCTTCGGAACTAGAGCC	42
AATTTAAGCGTTATATTATTCATTAAAGTTAGCAATGCGGGA	42
ACTTTTTGTAGGGCACATTCAACCGATTGCACCGTATATTCG	42
ATGCTGATGTAATTAGAAAATTCATATGTCAGACTTGCGCCG	42
TTTTAACTATAAAGCGCAAAGACACCACCCCTTATTGCTTTC	42
CAATAGTCGACAATCGTAGAAAATACATGAACCAGAAGGCTC	42
TGAAAACTTATCAACTGGCATGATTAAGCCCTCAGATTGCGA	42
TCTGTAACATCCTAGGAAACCGAGGAAACCACCAGACAGTTT	42
AAACAGTTGTCTTTTTTTAAGAAAAGTAGGTTGAGGTAAATG	42
AATTACACCGCACTCAAGAAACAATGAAAATAAATTTAGCGT	42
AGCAAAATAGGAATAGAGATAACCCACAAATTTACCAAACTA	42
TACAAAAGAAGGCTCACCCTGAACAAAGACAGGAGATAGGAA	42
AGAAACAAACCTCCCATAAAAACAGGGATGAGTAACCCTCAG	42
AACGTCATTAGTTGTAACGTCAAAAATGTTTCGGATTAGTAC	42
ACGTAAATTACCAACGCTAACGAGC	25
GCGATGGCCCACTAC	15
GTGAACCATCACCCAAATCAAGTTTACATTATTACAG	37
ACCCTAATGGGAAGAGCAACACTATCATAAACGTTACAAACG	42
CGGCGAATATGCGAACCAAAATAGCGAGCCCCAAATAGATGG	42
CGGGCGCTTGAGATGGGGGTAATAGTAATAGCATGACGACGA	42
TAACCACCTTGCCCTGCGGAATCGTCATTCTGGAGCTTTCCG	42
CGTACTAACCCAAATTTAAACAGTTCAGTTTTGAGCATTCGC	42
TCGTTAGACCTTCACAGGTCTTTACCCTAACCGTTGTGCGGG	42
GGGATTTGGACAGATGCATCAAAAAGATAGAAAGGTGTGCTG	42
TTTTTATACGGTCATCGCGTTTTAATTCTGCAATGCAGTCAC	42
TCACGCATCCGCGAAAACTCCAACAGGTAACGCAACAGGAGA	42

ATAACATACAACGGCTTTTGATAAGAGGATTATGATCATGCG	42
TTGCTGGCATCTTTTTGCTGAATATAATTAAAGCCCTTATGA	42
ACAGGAACCAACCTAACTAAAGTACGGTTAAATCATTTACGC	42
ATCGTCTTTCCATTTGATTCCCAATTCTGGTGGCAGCCCTGC	42
ACACGACCTACAGAAGATACATTTCGCAAATGGATGAGGGTA	42
GAACCCTCCTCAGCTAGCACCATTACCAGTGAATTGTTTAGT	42
AATATTTAGGCTTGAACCATCGATAGCAGAGGGAGGCCAACG	42
AGCCCTACAACAACGTTTGCCTTTAGCGGTTTACCTAACAAC	42
GCAGAAGATTTCTTTTTCGGTCATAGCCGGAATAACAGTAAT	42
CCTGCAAAGCCTTTAATCAAAATCACCGACATAAATTCTGTC	42
TCTAAAGTTTTTTCCCTCAGAGCCGCCAACTCCTTCAGAACG	42
ATCAATAGTGAGAACACCCTCAGAGCCGCGCAATAGAAAAAT	42
TGAGGAATGTATGGCAGCATTGACAGGAAGCAGATAATCAAT	42
AATAGATTAAAGTTGATATTCACAAACAATAGCAATATTAAA	42
GAAGTATTGTAGCAAAGCGCAGTCTCTGAGAATTGTTTTATT	42
TCCTTTGACCGTAATGGCTTTTGATGATTCAGAGGGCAAATC	42
TTATCATACCCTCACGGGGTCAGTGCCTAGCGCATGAGGCGT	42
TTATCATCTCAGAAATGCCCCCTGCCTAAAAATAGGCCTTAA	42
ATATAATAATAGGTAAGTATTAAGAGGCCAATCCATTTTATC	42
ATGGAAGGGTTAGAA	15
TTAACCAATAGGAACGCCATCAAAAATAAT	30
TGTTAAATAGTAAGAAAAATCTACGTTAAGCACTATTGGAAC	42
AGATTGTGATAAAATTTTAAGAACTGGCTTGACGGATCAAAA	42
TATGTACTGCCAGAGGTTTAATTTCAACGAAAGCGCCTGTTT	42
GAGAATCCCAATACTGACGAGAAACACCGGTCACGGCAGCAA	42
CAAAGGCCAAATGCTCAACGTAACAAAGGCCGCTAGCAACAG	42
ATAAATTCAAAAATTCAAGAGTAATCTTATAACGTTATTGGG	42
CAGTCAAAGCGGATTGAACGGTGTACAGGGAGGCCGCATTAA	42
AATGTGTTCAAATAATCATAAGGGAACCAATCCTGCTCACTG	42
AATTTTTCGGAAGCCCTGCTCCATGTTAAAAGAGTGGGTGCC	42
ATACTTTATTGCTCAGATTTGTATCATCTTCTTTGTCCACAC	42
ATAAAGCAGCTTAAGACCCCCAGCGATTTCAAACTGTCATAG	42
AAGGCAAAATATGCAAAACGAAAGAGGCCGCCAGCGACGGAG	42
TACTAATTAACAGTAAACGGGTAAAATACATTTTGTAAACAG	42
TATTTTCGACCATTGGCTTTGAGGACTACAGATTCAATCATT	42
TCACCGATCACCAGAGCGAAAGACAGCAGGCCAACTAAGGCG	42
AAATATTCCAATGACAGGGAGTTAAAGGTACGTGGTGACCTA	42
AAGACAAGAATCAACATCGCCCACGCATATGCGCGCGAGAAA	42
TTGTCACTCGGCATAAACAGCTTGATACCGAACGATATGTAA	42
ACATATATTTTCATAATTGTATCGGTTTCAGTATTACTACCT	42
AGTATGTCCGCCTCACGTTGAAAATCTCCAGCAAAGAAGAGT	42
AATACCCGAGCCACTAGAAAGGAACAACATATCAAGAATCCT	42

CAAAGTTCCGCCGCGATTTTGCTAAACACAGTTGACCTTGCT	42
CTTACCGTGGCCTTTTGTCGTCTTTCCAGAGCACTTTAATGG	42
CCCAATAGAATGGATTCCACAGACAGCCCATTTGACAAAATT	42
AGCGCTATCATACACACTGAGTTTCGTCCAACTCGTTACCTG	42
GAGAATTGTTTTAATTTTCAGGGATAGCAAGTTTGACCAAGT	42
TACAGAGACAGTTACCGCCACCCTCAGACAGAAGGCATCGGG	42
AACGATTAACATGAGTATCACCGTACTCATGGCAACAGGTTT	42
CATATTATTTATCCTGAGACTTAAGTAT	28
AATTTGCCAGTTACAAAATAAACAGC	26
TCGCGTCTGGCCTTC	15
CTGTAGCCAGCTTTCCCAACGTCAAAG	27
TGAGCGACACTATTTCGAGGTGCCGTAAATAAAACGGAATTA	42
GCGGATTCCGAGATCCCCGATTTAGAGCTCATTATGTTTACC	42
GCGCATCGTTCCGAAGAAAGGAAGGGAATTTAATCTGCAAAA	42
CAGTATCACGCTGGCTGGCAAGTGTAGCAGAACGAAGACTGG	42
GCACCGCCCCTTCAGCCGCGCTTAATGCCTGCTCACATTGAA	42
CATTCAGGTGGTTTTTTGACGAGCACGTGACAAGAGAATGAC	42
CCTCTTCGCCAACGGCGGGAGCTAAACAACCAGGCATAGTCA	42
CAAGGCGTCCAGTCGAACGGTACGCCAGGAACTGAAAGCCCG	42
GACGTTGTGAGCTAGAGGCCACCGAGTACTTAGCCAAAGCGA	42
AGCCAGGGAGCCGGCGTTGTAGCAATACGCCTGATAGAGAGT	42
CACGACTCTGTGTGCTGAGTAGAAGAACATACCAATGCGGAT	42
CAATGTCGGGTACCCAGAACAATATTACAAAAGAACTCAACA	42
TCGCCCTGCTACGTCATGGAAATACCTACGTAATGAGTTTCA	42
CATCTGTACTCTGAATTATTTACATTGGAAGACTTAGTAGAT	42
CCGGAATCATTTGGGATCAATAACCTGTGGGGCCTTGAAACA	42
ATCATATTGGTTTGTGAAAGCGTAAGAACCGCTTTGGCCGGA	42
CTCAACACAAATATGCTATTAGTCTTTAAACCGATAATCAGT	42
GCCAACATGCAAATGCCATTAAAAATACCGATAGTGTAGCGC	42
AAGAGAACTCCGGCAGAGGTGAGGCGGTATCAGCTTAGCGTT	42
CAGACGAGAATTTAACGCTGAGAGCCAGCAAAAAAAGCCACC	42
CGCCTGTATAGCGATTGCTGAACCTCAATAAAGGAAACCGCC	42
AATATCCATCGTCGAGTTGGCAAATCAAACTTTCAAACCACC	42
AATCGGCACATAAATAAAATATCTTTAGGACGTTAGCAGGTC	42
CCAAGTATTTAACAGTCAATAGATAATACTCATAGCCTCATT	42
TTCATCGGAAGATGTACAAACAATTCGAACCAGTACGTTCCA	42
AGATATATCGCGCAGTTATTAATTTTAAAAGCCCATGTACTG	42
TTTAGCGATAACGGAACAAAGAAACCACACCGCCACAGTGCC	42
ATCAAGAGATGAATCTGATTATCAGATGAGGAGGTACCTATT	42
CTGAATCACAGAAAGTTTGGATTATACTTCTGAATA	36
GTCTTTCCAGAGCCT	15
GTAGAAAGATTCATCAGTTGA	21

CGGAACATTTGGGGAAAGAACGTGGACTATCAACATTAAATG	42
AGGACGTAGGGAGCAGGGTTGAGTGTTGCGTGGGAAATATTT	42
ATTACCTCGTGGCGAATCGGCAAAATCCGTTGGTGAACAGGA	42
ATTGGGCTAGGGCGTTTGCCCCAGCAGGTGAGGGGTCAATCA	42
AATAAGGCACACCCCCGCCTGGCCCTGACAGCCAGCAAACAA	42
ATTCATTTGGTTGCTTCTTTTCACCAGTAAAGCGCAGATCTA	42
CTGGCTGAATCAGACGCGGGGAGAGGCGGCGATCGCTAGCTG	42
TGAAAGATAGACAGGGGAAACCTGTCGTAGGGGGACCGGAGA	42
GGCGCAGAATCAGTACTCACATTAATTGGTTTTCCCCTGAGT	42
GTCGAAAAATTAACAAGCATAAAGTGTAGCTTTCTGGATAAA	42
ACAAAGTCACTTGCAAATTGTTATCCGCTGTGAATCCCTGTA	42
AAACACTTAATATCGAGCTCGAATTCGTTGTCAACTCAGAGC	42
GAAGGCAAAACGCTGGTGCTTGTTACCTTAATCTATACAGGC	42
AGGAAGTGAAATGGCCTCCTGGTTGGTGCAGTGCGTCAATTC	42
GCAACGGCAGTAATTCGGCTGACGCATTGAATATATTAGCTA	42
TCGTCACTCTGACCAAATACCGACCGTGAAAGCCTATCACCG	42
GTCGCTGTTGAATGATTTTAGTTAATTTGTATAAAGGAAGGT	42
ACAATGAAAACATCCCAATCGCAAGACACCATATTAGCGCCA	42
GAGGTGAATAAAACTTAGGTTGGGTTATTTCGAGCGTTTATT	42
CAAAAGGCAGTGCCTCAAAATCATAGGTAAAGTAAGGTGGCA	42
ATAATAACATCACCTAGCTTAGATTAAGGCTAATGATTACGC	42
CAGCGGATCTGGTCCTATTAATTAATTTTGAACAAATAACGG	42
AATTTTCGGTTATCTCAATATATGTGAGAGAAACCAGCCGAA	42
AACGATCTAGAGCCATTTCATTTGAATTGAACGGGTAGCTAT	42
CAACGCCTAGACTTATGAAACAAACATCAAGCCGTAGTTAAG	42
CCCATGTCCCGAACGAGGCGAATTATTCATAGCAAGTAATTG	42
AGCCACCTTTGCGGATTCGCCTGATTGCAGAACGCTAGACGG	42
CGCCACCCATATTCATACAGTAACAGTATTTTGAACAGCCTT	42
AGCCCGGCCTGATTTAAAGAAATTGCGTGCTACAAAATAAGA	42
CGAGAGGGTTGATACCTCAAGAGAAGGATTAGGATT	36
AGTACCAGGCGGATAAGTGCCGT	23
GATTTAGGAATACCA	15
CATTCAACTAATGCAGATACATAACAATCAGCTCATTTT	39
CGAGGCAATTCGCACCCGTCGGATTCTCTTCCAGTAATCGGA	42
AGACGACATAAGCAATGGGATAGGTCACCTTATAAGGAAAGC	42
GAAGTTTCCCGGTTTGCATCTGCCAGTTCGAAAATAAAGGAG	42
ATAGCGTGATGAACGGAAGATCGCACTCGAGAGTTCTGCGCG	42
TCCCCCTTATCAGGGCCGGAAACCAGGCGAGACGGCAGGGCG	42
CATAAATAATGCCGAACTGTTGGGAAGGGTTTGCGGCTTTCC	42
GAAGCAAATCACCACGCCAGCTGGCGAAGCCAGCTGATTAAA	42
AAAGACTAGGTAAATGGGTAACGCCAGGCGTTGCGAGAAGTG	42
ACCAGACAGAACCCACGGCCAGTGCCAAAAGCCTGCTGTCCA	42

ACCTTTATGCGGGATTCTTCTAAGTGGTTCACAATATTAGTA	42
GGCTTAGTAAATCGCCTTAGTGCTGAATAATCATGATCGGCC	42
TGTTTTAAGAATTAAATAACCCCGCTTCCGATAAACATTGCA	42
TTCCATAAGTAGTACTCTATGATACCGATAATGAGACGCTCA	42
TTAGTTTATTTGGGTCGTCGGTGGGCACTCACATAACCAGTC	42
AGCAAAACTTGAGCCATAATTACTAGAATGATAAAAGAGATA	42
AACGTCAGACGGAAACAAATTCTTACCACATCTTCCACAGAC	42
AGCGACAAAGGGCGTTAATTGAGAATCGAAGAACGAACTGAT	42
GTTTTCAAATCAATTAGGCAGAGGCATTATAACTAACCACCA	42
TGCCATCAAAGAAATACCGACAAAAGGTCTGAGAGAACACCG	42
ACCGGAATAGCAAAAAACAACATGTTCAACGCTGATGAAAAA	42
ACCCTCAAAAAGAACAATAGATAAGTCCTCCCTTAACCCTCA	42
ACCAGAGACCAGAAATTTACGAGCATGTTGAATAAAAGGAAT	42
AGACGATAAGCCCTCCTTATCATTCCAAACCTTTTAACAACT	42
AAAGCCAATAAGAGCATCGAGAACAAGCAAGAAAAGGATTTA	42
GTAAGCGATATCAGCATTACCGCGCCCAATTTCAATATTAAA	42
GTAATAAAACTGAATATCCGGTATTCTATTTGAATAGTAACA	42
CGTATAAAGAATAACGACTTGCGGGAGGCCTTTTAAGCGGAA	42
ATTCTGATTTTGTTCTATTTTGCACCCAAGATTTTTTCATCA	42
AGCGGGGTTTTGCTC	15
object-label:	
Cy5-TTCCTACCATATCAAAATTATTTGC	25

Supplementary Table 3: list of oligonucleotide-sequences of the 6-helix-bundle.

sequence	length	defect-variant
ATCATATTGGTTTGTGAAAGCGTAAGAACCGCTTTGGCCGGA	42	3
CTCAACACAAATATGCTATTAGTCTTTAAACCGATAATCAGT	42	3
GCCAACATGCAAATGCCATTAAAAATACCGATAGTGTAGCGC	42	3
TCGCCCTGCTACGTCATGGAAATACCTACGTAATGAGTTTCA	42	5
CATCTGTACTCTGAATTATTTACATTGGAAGACTTAGTAGAT	42	5
AGCCAGGGAGCCGGCGTTGTAGCAATACGCCTGATAGAGAGT	42	8
CACGACTCTGTGTGCTGAGTAGAAGAACATACCAATGCGGAT	42	8
CAATGTCGGGTACCCAGAACAATATTACAAAAGAACTCAACA	42	8
CAAGGCGTCCAGTCGAACGGTACGCCAGGAACTGAAAGCCCG	42	10
GACGTTGTGAGCTAGAGGCCACCGAGTACTTAGCCAAAGCGA	42	10
AAGACAAGAATCAACATCGCCCACGCATATGCGCGCGAGAAA	42	15
TTGTCACTCGGCATAAACAGCTTGATACCGAACGATATGTAA	42	15
ACATATATTTTCATAATTGTATCGGTTTCAGTATTACTACCT	42	15
AGTATGTCCGCCTCACGTTGAAAATCTCCAGCAAAGAAGAGT	42	15
AATACCCGAGCCACTAGAAAGGAACAACATATCAAGAATCCT	42	15
ATAAAGCAGCTTAAGACCCCCAGCGATTTCAAACTGTCATAG	42	20
AAGGCAAAATATGCAAAACGAAAGAGGCCGCCAGCGACGGAG	42	20
TACTAATTAACAGTAAACGGGTAAAATACATTTTGTAAACAG	42	20
TATTTTCGACCATTGGCTTTGAGGACTACAGATTCAATCATT	42	20
AAATATTCCAATGACAGGGAGTTAAAGGTACGTGGTGACCTA	42	20
GAACCCTCCTCAGCTAGCACCATTACCAGTGAATTGTTTAGT	42	30
AATATTTAGGCTTGAACCATCGATAGCAGAGGGAGGCCAACG	42	30
AGCCCTACAACAACGTTTGCCTTTAGCGGTTTACCTAACAAC	42	30
GCAGAAGATTTCTTTTTCGGTCATAGCCGGAATAACAGTAAT	42	30
CCTGCAAAGCCTTTAATCAAAATCACCGACATAAATTCTGTC	42	30
TCTAAAGTTTTTTCCCTCAGAGCCGCCAACTCCTTCAGAACG	42	30
ATCAATAGTGAGAACACCCTCAGAGCCGCGCAATAGAAAAAT	42	30
AATGTGTTCAAATAATCATAAGGGAACCAATCCTGCTCACTG	42	30
AATTTTTCGGAAGCCCTGCTCCATGTTAAAAGAGTGGGTGCC	42	30
ATACTTTATTGCTCAGATTTGTATCATCTTCTTTGTCCACAC	42	30
TTTTAACTATAAAGCGCAAAGACACCACCCCTTATTGCTTTC	42	40
CAATAGTCGACAATCGTAGAAAATACATGAACCAGAAGGCTC	42	40
TGAAAACTTATCAACTGGCATGATTAAGCCCTCAGATTGCGA	42	40
TCTGTAACATCCTAGGAAACCGAGGAAACCACCAGACAGTTT	42	40
TTTTTATACGGTCATCGCGTTTTAATTCTGCAATGCAGTCAC	42	40
TCACGCATCCGCGAAAACTCCAACAGGTAACGCAACAGGAGA	42	40
ATAACATACAACGGCTTTTGATAAGAGGATTATGATCATGCG	42	40
TTGCTGGCATCTTTTTGCTGAATATAATTAAAGCCCTTATGA	42	40
ACAGGAACCAACCTAACTAAAGTACGGTTAAATCATTTACGC	42	40
ATCGTCTTTCCATTTGATTCCCAATTCTGGTGGCAGCCCTGC	42	40
CCCGCTTATTAAGTGATTCAAAAGGGTGTAAGAGGCCAACTT	42	50

TAATGAGTAAAACGTCATATATTTTAAAGAGCTTCGGAACGA	42	50
AACATACGTGGATGGAAGCCTTTATTTCCAGGATTAAATTGT	42	50
CTGTTTCTAAGTGTGTTGTACCAAAAACTCATTTTGCGCGAA	42	50
GATCCCCCCGCCAAGCAAAATTAAGCAAGCTGTAGTACACTA	42	50
GGCTTAAGGAGTGAGCATTAACATCCAAGTCTGGACCACTAC	42	50
TCTCCGAAAGCAACGCGCGAGCTGAAAAGCGAACGTTTCATG	42	50
AATTTAAGCGTTATATTATTCATTAAAGTTAGCAATGCGGGA	42	50
ACTTTTTGTAGGGCACATTCAACCGATTGCACCGTATATTCG	42	50
ATGCTGATGTAATTAGAAAATTCATATGTCAGACTTGCGCCG	42	50

Supplementary Table 4: list of omitted oligonucleotides for the pseudo-defect-variants of the 6-helix-bundle.

Sequence	Length
AGGGCGAAAAACCGTCTATCAGGGCG	26
ACGTCAAAAATCAAACCCTCATATCTAAAATATCTTTTAATGGCCTGTT	49
TATTAAAAGGTGCCTTGCTGACACTAACAACTAATAATTTCATTATACA	49
CAGTTTGCGGAACCTGAAAAAAGCCGTCAATAGATTTTTTCATAAAGCC	49
AGATAGGCGATTTAACGCTGATTGAGGATTTAGAAAGACAAAGGGCTTA	49
ATAAATCAAGCCGGAACACCGACTTTACAAACAATGCTGATGTATTTAA	49
CCGAAATAAGGAAGAGAGGTGCTCGTATTAAATCCGTTATATAATTTAG	49
AAATCCTGGAGCGGACCACCAGAACGTTATTAATTTTAACCTGAGCCAG	49
CTGGTTTGCAAGTGGCCATTATTTGAGTAACATTATAGGTCTAAAGTAC	49
AGTTGCACGCGTAAAACTGATGCGGAACAAAGAAAATAGTGAGTAATTC	49
TTCACCGGCGCTTAGCTATTAAAGGAGCGGAATTATTAAGACCAATAAA	49
ACGGGCAGGCGCGTCACAGACATTCCTGATTATCAAAAACATAATGCAG	49
GTTTTTCGACGAGCTGAAAGCGCAATTCATCAATAAATTTTCTCAACAA	49
TGCGTATTTCCTCGAGAGATAGATTGTTTGGATTATGTAAATACAAGAA	49
AACGCGCGGAGCTAAAAAGGGGAATAATGGAAGGGGTGAGTGCCTAATT	49
AGCTGCATAAAGGGACCAGTCCTACCATATCAAAAACAGTACAACCAAT	49
AGTCGGGCGGTACGATTATTTCACGTAAAACAGAAGAATTACCTTTCCT	49
TGCGCTCAGTGTTTACGCTCAAATTGCGTAGATTTTTACATTCGGGTAT	49
GCTAACTGCCACCGCATGGAATAACGTCAGATGAAACATCAACACTCAT	49
CCTGGGGTCCATCACATTGCATAACAGTACCTTTTCAAAAGACCGTTTT	49
CCGGAAGTGTAGCACAGAACAGAGAAACAATAACGTATTCATGAATCAT	49
CAATTCCAGTAATAATCGGCCCTGATTGCTTTGAATACCAATGAAGAAC	49
TGTGAAAAGCGGTTGATAATCTTCTTAACGTTTGCCATCTTT	42
CATGGTCCCCCAAAAACTAGCATACTGCGGAATCGTTGCAAAGACAATG	49
TACCGAGATAAGCAATCGATGTATTCATTGAATCCAACCAAACCACGCA	49
TAAAGACAAACGTTGAGTCTGATGCTTTAAACAGTCGTTTACGGTCGCT	49
ACGTGGTATTCGCAGGCTATCACGAGAATGACCATGAGCAACGTTAAAG	49
TGAGTAAAATCAGCTATTTTTAAATCAGGTCTTTAAGGAATTATCGTCA	49
CTGACCTATAGGAAATTAATGTATTATAGTCAGAAATGCAGAAGACAGC	49
CATAAATATTCGCGTTCAACCGGATTGCATCAAAATAGGAATAGCAACG	49
TGAATCGAGCCAGCCAAATCAGAGGAAGCCCGAAAAAAGATTGAGGACT	49
TGGGCACAATGTGAGTGAGAAAATATCGCGTTTTAGAACAACGAGGAAG	49
CATCTGTGTCGGATTGTAGGTCTTCAAAGCGAACCCGTTAATGTAAAAT	49
ATACCGAAACGGCGAAATGCAAAGCAAACTCCAACGACGTTGCGAAGGC	49
TCGCCCTGGATAGGTTTAGAAGATTAGAGAGTACCCTGGCTCAAAGAGG	49
CCGCTTCATGGGCGTTCAACGGCTCCTTTTGATAATACCTTAAAAACAC	49
CAATGTCATCTGCCTTTTGCGTTTTTGCGGATGGCTCAACTTCAGCGAT	49
GCTGAATACGACAGAACATTATTAATTGCTGAATATGGGCTTAACAAAG	49
CACGACTAGATCGCAGCTAAAGTAGCTCAACATGTACACCAGGTATCAT	49
AAGTGGTTCCGGCACAATAAAATGCAACTAAAGTATAAGGCTTGTCGAA	49
AGCCAGGGGAAACCCAAAGAATGGAAGTTTCATTCCAAAGCTCCATGTT	49

GTGCCAATCGCCATCAATAAACAGTTGATTCCCAATCATTACAGGCGCA	49
GACGTTGTGTTGGGAATAGTAAACGAGTAGATTTAATCTTGAAGGGAAC	49
GATGTGCTGCAAGGCGATTAAGTTGGGTAACGCCAGG	37
ATGGCCCACTACGTGAACCATCACCC	26
CGGGCCTCTTCGCTATTACGCCAGCTGGCGAAAGGGG	37
ATCAATATCTGGTCAGTTGGCAAATC	26
ATATCAAGTTTTTTCCCTTTTAAAAGAACTGGCAT	35
CATCACCGTAAAGCGCAATAGACTCCTTATTACGC	35
CAGCAAACTAAAGGAGAGCAATAGCAAACGTAGAA	35
CAGTGCCGAGCTTGATTGAGTACATAAAGGTGGCA	35
CAGTATTCGAACGTTCAGAGAAAAGAAACGCAAAG	35
ATAAAACGGAAGAAGAGGGTAGGAATAAGTTTATT	35
CGAACGAGCGCTAGTGAACACAATCAATAGAAAAT	35
AAACATCTAGCGGTGCATTAGGTTTACCAGCGCCA	35
ATGCGCGCCACCACATAACATAAGGGCGACATTCA	35
TTGAATGATGCGCCATAGCAGGAGGGAGGGAAGGT	35
TACGTGGACTATGGTGTTTAAGACGGAAATTATTC	35
TCTGACCACGTATATCCAAATGTGAATTATCACCG	35
GGCCAACTTAGAATCAGCCATCTTGAGCCATTTGG	35
CAGTAATAACAGGAATTTGCCAGCCAGCAAAATCA	35
CAGATTCATTTTAGCGAGCGTCACCATTACCATTA	35
GAAATGGCCAGAATCCTGAATCGGAAACGTCACCA	35
CATTTTGTTATAATTGCACCCCATCGATAGCAGCA	35
AAACGCTAGTAAAAAATCAAGCAGTAGCGACAGAA	35
CGCCAGCCGCAAATGCGGGAGTGCCTTTAGCGTCA	35
TAATATCATACTTCTTTTAGCGCGCGTTTTCATCG	35
TCAAACTACATCACGTATTCTCGGTCATAGCCCCC	35
TCATATGTACCCTAGTTTAGACTGGATAAGGGGGTCTTGATA	42
ATCGTAAAACAGGACAGCTTGCAAAATCACCGGAA	35
CAAGAGAAATATTTCCTTTAACACCACCGGAACCG	35
TGCCTGAAATATTTAAAAAAACAGAGCCGCCACCC	35
CTACAAATTAAATTTTTTCACCGCCACCCTCAGAG	35
GGGTAGCTCATTTTAAGGAATCCTCAGAGCCGCCA	35
CTGATAACGCCATCGAGAATACACCACCAGAGCCG	35
TATGATATCTGGCCTTTCAACCATTGACAGGAGGT	35
AGACAGTTTTCATCTATGGGAGGTCAGACGATTGG	35
CAAAAGGGCGAGTACGTTAGTATTCACAAACAAAT	35
AGTAATGTCTCCGTAAGTTTTCATTAAAGCCAGAA	35
ATATTTTGATTGACCATAGTTCGCAGTCTCTGAAT	35
AAAAATTTCACGTTTAGCATTTCCAGTAAGCGTCA	35
CCTTTATCATCGTACAGTACACTTTTGATGATACA	35
GTAATACAGTTTGACGTAACAACTGGTAATAAGTT	35

TACCAAATATCGGCGCCCAATGGTCAGTGCCTTGA	35
AGCATAAACTCCAGCCTCATTTGCCCGTATAAACA	35
AATTAAGCCGCTTCCGCCACCCCCCCTGCCTATTT	35
GGCAAGGAGGCAAACAGAACCTATTATTCTGAAAC	35
TAACATCTCAGGCTGAGGTTTTATTAAGAGGCTGA	35
TTCTACTAAGGGCGTAGGTGTCAAGAGA	28
AGCTATATTTTCATTTGGGGCGCGAGCTGAAAAGGTG	37
CATTAGATACATTTCGCAAATGGTCAATAACCTGTTT	37
GTTTGAAATACCGACCGTGTGATAAATAAGGCGTTAA	37
CTTCATCAAGAGTAGTTTGACGCATCAA	28
CAGACCAGGCGCATAGGCTGGCTGAC	26
ATAAGAATAAACACCGGAATCATAATTACTAGAAAAA	37
TAGTATCACCGAAGGGGGTCGGAACGTGGACTCCA	35
AATTCTTTGAAATAACTAAATGAACAAGAGTCCAC	35
AACGCTCAATAATAGAGCCCCGTTGAGTGTTGTTC	35
ATTGAGACACAAGAACGGGGAAAAAGAATAGCCCG	35
CAACGCCGCTAATAGGCGAGACGGCAAAATCCCTT	35
GCAGAGGAAAGTCAAGCGAAAGTTTGATGGTGGTT	35
TAATAAGAATTAACGGCGCTGGCCCCAGCAGGCGA	35
CGACAAAGGGAAGCCACGCTGGCAAGCGGTCCACG	35
TGTCCAGAGAGAGAACCCGCCCCTGGCCCTGAGAG	35
CAACATGAATGAAAGCTACAGACAGCTGATTGCCC	35
AACGCGCGATTTTTTTGCTTTTTTTCACCAGTGAG	35
TAGATAAATCCCAAACGTGCTTGGGCGCCAGGGTG	35
AAATAATAAATAAACAGAGCGGGGGAGAGGCGGTT	35
TACGAGCGAGCCTAGGCCGATTTAATGAATCGGCC	35
CAATAATACGCTAAACAGGAAAAACCTGTCGTGCC	35
TATCATTATTTTATCCTGAGAACTGCCCGCTTTCC	35
TAAACCAGCTATTTCAGTGAGCACATTAATTGCGT	35
CGAGAACAGCCTTAGAGTCTGTGCCTAATGAGTGA	35
TATTTTCCCGACTTTAACCGTCATAAAGTGTAAAG	35
TACCGCGCGAGGCGTTTGATTACACAACATACGAG	35
AATCAGATATAGAGAATAGTAAAAGTTACAAAATCGCAAGCA	42
CCGATAGGGTGAATAGAAAAGATAGCTGTTTCCTG	35
ACAACAAGGTTTATAGATTGTCTCGAATTCGTAAT	35
TAACCGAAAAGGAGAAATTGTGGAGGATCCCCGGG	35
GAGGCTTATCTCCATGTTAAAGCTTGTTACCTCGA	35
GCCGCTTAATAATTTTTGTTAACAGGGCTTAAGCT	35
CCCTCAGACAACTATTAACCACCTGGTTGGTGTAA	35
ATCGGAAGCGGAGTAAAAATACATTTCTCCGAACT	35
GCTACAGAAACAACTTCCTGTGCTGACGCATTTCA	35
AAAGACTTTTTCTGAACATTAGAATATAGGGGCCT	35

TTTCCATTTCCAGAACAACCCAAGCAACTCGTCGG	35
ACGTAATCGATCTAGGGAACACAGTGCGGCCCTGC	35
ACCAACCCAGCCCTCGTAATGGGAGTGACTCTATG	35
CAAAAGAACGCCTGGGTGTAGTAATCTATTTACGC	35
TCATCTTTCGTCACACCGTGCCCGCCAAAATAACC	35
TATACCACATGTACGGGGACGTGTCAACCTTATGA	35
TACAACGATAGCAACTCAGGATAAGTGTCCTTAGT	35
CGCCTGACCACCACCCAGCTTTGTGAATTCATGCG	35
ATCCGCGTCAGAACTGGTGCCGTGGATGTTCTTCT	35
ACTTAGCCCACCCTGCGCCATGCTTTCTCAGGAGA	35
GACGGTCTACTCAGGCGCAACTAAAACGACGGCCA	35
CGAACTGCCCGGAAATCGGTGGTTTTCCCAGTCAC	35
ACCAACTTTGAAAGAGGACAGATGAACGGTGTA	33
TAAGAAAAGTAAGCAGATAGCCGAACAAAGTTACCAG	37
TAAGTGCCGTCGAGAGGGTTGATATAAGTATAG	33
AAGGAAACCGAGGAAACGCAATAATAACGGAATACCC	37
GATTAAGCTATCTTATATGCGTCTTCTGACCTAAATTAGGAGACCTCAA	49
AGTATGTGAAACAAACCAGTAAATATATTTTAGTTAGATTAGTCTAAAG	49
AATACATTAAGCCCAACAGTAGAACGCGAGAAAACAATACATGAGCCAG	49
ACATATAGATAACCATCGCCACAAATCCAATCGCAGTATTAGCCTGCAA	49
ACACCACATTGAGCAACATGTAACTATATGTAAATTCGACAAAGGCGGT	49
TTGTCACCCTGAACCATTTTCCCGGCTTAGGTTGGTTTGCCCGCAGAAG	49
TCATATGACGGGAGAGAATATGAGAGACTACCTTTTTAAAAGAAAATAC	49
AAGACAAAAAAACAAGGTAAAATTTATCAAAATCATCATTTTAGCCCTA	49
ACCGATTCCTTTACACGACGAGCTGAGAAGAGTCACCACCAGGTCTTTA	49
AAATATTCGTCAAATTCAGCTAGCGATAGCTTAGATCATCATAATATTT	49
ATTAAAGAAGAAACCTGTTTACCTTAGAATCCTTGGATGATGGTAAGAA	49
TCACCGAATTATTTGTCCTGACGTCGCTATTAATTTAATCCTGAACCCT	49
GAATTAGAGTTACAATCCCATAATAACCTTGCTTCTACTTCTACATTCT	49
CCAGTAGCTTTCCAATGTAGAATAAATCAATATATTTAGAACACACGAC	49
GCAAGGCCTTACCACGGCTGTCTTTTTTAATGGAATTATTTGACATTGG	49
ATGAAACAGCTACACCAAGAATAACAATTTCATTTATAAAGAATCGTCT	49
CCGTAATATTAGTTAGTACCGGAAAACAAAATTAATCAGGTTATACCTA	49
TCAAGTTGTTTTGAAAGCAAGAGATGATGAAACAATATACAGACAGGAA	49
GACTGTAGAACCTCATCGTAGTTCAATTACCTGAGACATCGGATATTAC	49
GCATTTTAAGAACGCCCAATAGCGCAGAGGCGAATGATTCGCTTGCTGG	49
TTATTAGACAAGGCTTATCCGTTGCCTGTTGTTATCCGCTCA	42
TCATAATCTTTCGATTGCGCCAGAAGTTTTGCCAGGCGTCCAATGTCAA	49
CCAGAGCTTGTATCCCATCGCATAGCGAGAGGCTTTCATAAAAACGGTA	49
CCTCCCTGGCTCCATATATTCCAGACGACGATAAACCCTCAAGAGCAAA	49
TCAGAACGTTGAAAGCAGGGAACTATCATAACCCTTCAGAAAAGGTCAT	49
CCACCACTGCGAATTTGCGGGACGAGGCATAGTAAAATCAAGAGAGAT	49

CCAGAACGAAAGGACAGCGAATACATAACGCCAAACCCTGACCCGGAGA	49
CCGCCAGAGTTTCACGAGGGTACCACATTCAACTAGCAAAGCGTTCTAG	49
TGAGGCATTTTGCTAGGCTTTCATCAGTTGAGATTAGATTAACCATCAA	49
CCTTGATAAATGAATTTTCATATTATTACAGGTAGGACTTCAAGGCCGG	49
AAATCCTGTCGTCTTAAACGGAAAACGAACTAACGATTCGAGAAAGATT	49
TGGAAAGAGCGTAAGCCACTAGGAAGAAAAATCTAAGACCGGATGCCTG	49
TTACCGTCCACAGATAAAACGATTATACCAGTCAGAGGTCAGCCCTCAT	49
TACATGGAACTACAATACACTTGCGATTTTAAGAATTTAATTCAAGGAT	49
GGAGTGTCTGAGTTTGACCCCTAATCATTGTGAATGAGGTCAGGAGAAG	49
TTAACGGAGGAACCAGCGCGAGAGATGGTTTAATTTTAGAGCTGACCCT	49
GTAACAGTTCAGGGGAGATTTAACGAGTAGTAAATTAATGCTTCGGTTG	49
GTTAATGCTCAGAGTAAATTGTGCCCTGACGAGAATTTAAATGCCTCAG	49
CGGAACCGCCACCCACCTGCTGCTCATTCAGTGAACGGTGTCTTAGCAA	49
ATGAAAGAGTACCGCGGAACGCCAAATCAACGTAACATATAATCATACA	49
GACTCCTATCACCGAATCATACAAGAACCGGATATTTCTGCGGTAGCAT	49
AGGATTAGGATTAGCGGGGTTTTGCTCAGTACCAGGCGGA	40
object-label:	
Cy5-TTAACAGTTGAAAGGAATTGAGGAAGGT	28

Supplementary Table 5: list of oligonucleotide-sequences of the 8-helix bundle.

sequence	length	defect variant
AGATAGGCGATTTAACGCTGATTGAGGATTTAGAAAGACAAAGGGCTTA	49	3
CCGAAATAAGGAAGAGAGGTGCTCGTATTAAATCCGTTATATAATTTAG	49	3
CTGGTTTGCAAGTGGCCATTATTTGAGTAACATTATAGGTCTAAAGTAC	49	3
TACCGAGATAAGCAATCGATGTATTCATTGAATCCAACCAAACCACGCA	49	5
ACGTGGTATTCGCAGGCTATCACGAGAATGACCATGAGCAACGTTAAAG	49	5
GACTCCTATCACCGAATCATACAAGAACCGGATATTTCTGCGGTAGCAT	49	8
TATTAAAAGGTGCCTTGCTGACACTAACAACTAATAATTTCATTATACA	49	8
CCGGAAGTGTAGCACAGAACAGAGAAACAATAACGTATTCATGAATCAT	49	8
GTAACAGTTCAGGGGAGATTTAACGAGTAGTAAATTAATGCTTCGGTTG	49	10
CGGAACCGCCACCCACCTGCTGCTCATTCAGTGAACGGTGTCTTAGCAA	49	10
GACTGTAGAACCTCATCGTAGTTCAATTACCTGAGACATCGGATATTAC	49	15
CCAGAGCTTGTATCCCATCGCATAGCGAGAGGCTTTCATAAAAACGGTA	49	15
TCAGAACGTTGAAAGCAGGGAACTATCATAACCCTTCAGAAAAGGTCAT	49	15
CCAGAACGAAAGGACAGCGAATACATAACGCCAAACCCTGACCCGGAGA	49	15
GGAGTGTCTGAGTTTGACCCCTAATCATTGTGAATGAGGTCAGGAGAAG	49	15
TTACCGTCCACAGATAAAACGATTATACCAGTCAGAGGTCAGCCCTCAT	49	20
ATTAAAGAAGAAACCTGTTTACCTTAGAATCCTTGGATGATGGTAAGAA	49	20
GAATTAGAGTTACAATCCCATAATAACCTTGCTTCTACTTCTACATTCT	49	20
GCAAGGCCTTACCACGGCTGTCTTTTTTAATGGAATTATTTGACATTGG	49	20
CCGTAATATTAGTTAGTACCGGAAAACAAAATTAATCAGGTTATACCTA	49	20
CCGCTTCATGGGCGTTCAACGGCTCCTTTTGATAATACCTTAAAAACAC	49	30
GCTGAATACGACAGAACATTATTAATTGCTGAATATGGGCTTAACAAAG	49	30
AAGTGGTTCCGGCACAATAAAATGCAACTAAAGTATAAGGCTTGTCGAA	49	30
GTGCCAATCGCCATCAATAAACAGTTGATTCCCAATCATTACAGGCGCA	49	30
AATACATTAAGCCCAACAGTAGAACGCGAGAAAACAATACATGAGCCAG	49	30
ACACCACATTGAGCAACATGTAACTATATGTAAATTCGACAAAGGCGGT	49	30
TCATATGACGGGAGAGAATATGAGAGACTACCTTTTTAAAAGAAAATAC	49	30
ACCGATTCCTTTACACGACGAGCTGAGAAGAGTCACCACCAGGTCTTTA	49	30
TGAGGCATTTTGCTAGGCTTTCATCAGTTGAGATTAGATTAACCATCAA	49	30
AAATCCTGTCGTCTTAAACGGAAAACGAACTAACGATTCGAGAAAGATT	49	30
AAATCCTGGAGCGGACCACCAGAACGTTATTAATTTTAACCTGAGCCAG	49	40
AGTTGCACGCGTAAAACTGATGCGGAACAAAGAAAATAGTGAGTAATTC	49	40
ACGGGCAGGCGCGTCACAGACATTCCTGATTATCAAAAACATAATGCAG	49	40
TGCGTATTTCCTCGAGAGATAGATTGTTTGGATTATGTAAATACAAGAA	49	40
AGCTGCATAAAGGGACCAGTCCTACCATATCAAAAACAGTACAACCAAT	49	40
TGCGCTCAGTGTTTACGCTCAAATTGCGTAGATTTTTACATTCGGGTAT	49	40
CCTGGGGTCCATCACATTGCATAACAGTACCTTTTCAAAAGACCGTTTT	49	40
CATAAATATTCGCGTTCAACCGGATTGCATCAAAATAGGAATAGCAACG	49	40
TGGGCACAATGTGAGTGAGAAAATATCGCGTTTTAGAACAACGAGGAAG	49	40
ATACCGAAACGGCGAAATGCAAAGCAAACTCCAACGACGTTGCGAAGGC	49	40
ACGTCAAAAATCAAACCCTCATATCTAAAATATCTTTTAATGGCCTGTT	49	50

CAATTCCAGTAATAATCGGCCCTGATTGCTTTGAATACCAATGAAGAAC	49	50
GACGTTGTGTTGGGAATAGTAAACGAGTAGATTTAATCTTGAAGGGAAC	49	50
TCATAATCTTTCGATTGCGCCAGAAGTTTTGCCAGGCGTCCAATGTCAA	49	50
GATTAAGCTATCTTATATGCGTCTTCTGACCTAAATTAGGAGACCTCAA	49	50
CAGTTTGCGGAACCTGAAAAAAGCCGTCAATAGATTTTTTCATAAAGCC	49	50
CATGGTCCCCCAAAAACTAGCATACTGCGGAATCGTTGCAAAGACAATG	49	50
TAAAGACAAACGTTGAGTCTGATGCTTTAAACAGTCGTTTACGGTCGCT	49	50
TGAGTAAAATCAGCTATTTTTAAATCAGGTCTTTAAGGAATTATCGTCA	49	50
ATAAATCAAGCCGGAACACCGACTTTACAAACAATGCTGATGTATTTAA	49	50

Supplementary Table 6: list of omitted oligonucleotides for the pseudo-defect-variants of the 8-helix-bundle.

Sequence	Length
AGAAGTGCGGTTTAGTCAGGACGCACCACCAGAGCGCCTTTA	42
TTGAATGGCTATTAGTCTTTAATGCGCGAACTGATAG	37
AAAGAACGTGGACTCCAACGTCAAAGGGCGAAAAACCGTC	40
AAGAGTCTTTGGGGTCGAGGTAACACTCCTCCATG	35
AGTGTTGAGCACTAAATCGGAAGAGGCACGAGGCG	35
GAATAGCAGGGAGCCCCCGATAAGGCACTAAGGGA	35
AAAATCCTTGACGGGGAAAGCAAAATACCTTTGAA	35
GATGGTGCGTGGCGAGAAAGGGGAAGTTCGGTGTA	35
CAGCAGGGAAAGCGAAAGGAGGGACTAAAGGCTGG	35
GCGGTCCTAGGGCGCTGGCAACAACGGCGAGTAAT	35
GCCCTGAGGTCACGCTGCGCGACAGCATGATATTC	35
CTGATTGCACACCCGCCGCGCCGTCACCCGTAACA	35
CACCAGTGCCGCTACAGGGCGTAAAGGCGTGAATA	35
CGCCAGGTGGTTGCTTTGACGTCGCTGAGAGAAAC	35
AGAGGCGATAACGTGCTTTCCACGCATATAAATTG	35
TGAATCGAATCAGAGCGGGAGCAATGACTAATTTC	35
CTGTCGTGGAGGCCGATTAAATGATACCTGAATTA	35
CCCGCTTTAGACAGGAACGGTAGGTGAAAAGAACT	35
TTGAGTAAATCAGTGAGGCCAAAAAGGACGCCGCC	35
AACGTTAAAAGAGTCTGTCCAAATCTCCGTTGAGG	35
TCGTATTAATTAACCGTTGTATAATAATGGCCTTG	35
CTTTACATTCTTTGATTAGTAAACAACTATAAATC	35
TGAGGATCACTTGCCTGAGTAAGCGGAGAATGGAA	35
GCCGTCATCAAACTATCGGCCTAAACAAATTTACC	35
ACTAACATAATATCCAGAACAATTTTCTCATACAT	35
ATCTAAACGCCAGCCATTGCATTTCCAGCAGGAGT	35
TGAAAGGAAACGCTCATGGAAACGATCTTTTTAAC	35
GTCAGTTCATTTTGACGCTCAACAGCCCGAGTAAC	35
CAAACCCGAAATGGATTATTTAACGCCTCAGTTAA	35
ACCTTGCCAGATTCACCAGTCTTCGTCATTCGGAA	35
AAATGAACAGTAATAAAAGGGCCATGTAACATGAA	35
GCCACGCGGCCAACAGAGATAGATAGCAGAGACTCC	36
ATTAACATCTGACCTGAAAGCGCCACCATAGGATT	35
AACAGAGTACGTGGCACAGACCTCAGAACAGTACC	35
CCCTAAAACATCGCCATTAAAAATACCGAACGAACCA	37
CCAAAATAACCCCGCTTCTAATCTATTTACGCTCGCCCTG	40
AAACAAAATTAATTACATTTAACAATTTCA	30
GTCCTTAGTGCTGAATTGTCAACCTTATGACAATGTCCCG	40
ATTGCATCTGCCAGGAGTGAGCTAACTTGGGAAGATCATTTG	42
AACAGTAACGGGTACGTAGGA	21
TTTGAATTACCTTTTTTAATGGA	23

AGGTCATTGCCTGAGAGTCTGGAGCAAACAAGAGAAT	37
CGGTAATCGCACGATCTATGA	21
TTAAACCAAGTACCGCACTCATCGAG	26
GAGGGTAGCTATTTTTGAGAGATCTACAAAGGCTATC	37
AAATTAAAAGAGGAAGAAAACCGACCTGATCTTTG	35
TATTCAACAAATATCTCAAATGCCGGAAAAAGAAT	35
GTCAAATAGCTTCAATAAATATCAATCACAACCTA	35
GGGTGAGGGAAGCAGTCCAATTGACCAAGTAATGC	35
TGTGTAGAGGATTAAAAATGTAGATGAATCCATTA	35
TTAAATGTTGCTCCTTTGCCAGGCGCATAGACTTT	35
TTTTTAGCATTTTTAGAGGCTTCATCAATACAGAG	35
ATTTCAAGCTTAATACGATAAAGAACCGCGGAACG	35
ACTTTTGCTGTAGCATAACCCAAATCAACTCAGCA	35
AAAACATATATGCACATAGTATCATTCACGCTTTT	35
AAAGCTATCTGGAAACGCCAACCCTGACGGCTTGC	35
AGCAATAAACAGTTTTCAACTCGAGTAGACCGATA	35
GGCAAAGCGAACGATTGAGATGATGGTTAACAACC	35
TCCAATAACCATTAACAGGTAATCATTGGATAGTT	35
CTAATAGATGGTCAAACTAACCGATTTTTTTCTTA	35
GAAAAGGTATATTTAAAATCTTATACCATCAGCTT	35
GAATTAACTGAAGCTAATTACTAGAAAAGAATAAAACAAAGA	42
AGGGAAGTGAGCGCCAGAGCCACAGGAGAAAAAAA	35
CAGAGAGTAACCCACACCCTCGACGATTTTTTTCA	35
AAATGAAAGCCCAAAACCGCCCAAACAAAAAGGAA	35
CGATTTTAACAATGCGGAACCAAGCCAGTGAGAAT	35
TATCCCAATCTTACTCTTTTCTCTCTGACTTTCAA	35
AAAATAAAGAAAAGCCCCCTTTAAGCGTGTATGGG	35
AGAGCCTAACAAAGCATCGGCGATGATAACGTTAG	35
AACGCTACCGAGGACGTCAGATAATAAGAAAGTTT	35
AATTTTAGGAATACCAGAATCGTGCCTTTCATAGT	35
TGCTATTATGATTACAGCACCGTATAAAGTAGCAT	35
AAGCCTTGCAGTATCACCAATTGCCTATCCAGTAC	35
CCCGACTAAAATACCATTAGCTTCTGAACCGTAAC	35
GCGAGGCCAACATAAATCACCAGAGGCTAGCCCAA	35
CTTATCCAGACACCTTTGGGAGAAGGATCCCTCAT	35
AAGCAAATTTTGTCCACCGTCTTTTGCTCCGCCAC	35
ATCATTAATTCATATATTCATTAAGTGCTCAGAAC	35
AACAAGCAAGCCGTTTTTATTTTCAT	26
TATTATAGTCAGAAGCAAAGC	21
GGATTGCATCAAAAAGATTTGCCGGACGATGAA	33
AAGACTTCCGTTCTCTAGCATGTCAATCCTAAGTGCCCTGCC	42
TAATTCGCACCATCCCCCGGTTGATAATGAAGCCACGTCGGT	42

CCAGACCAAAGGCCGCCCCAAAAACAGGCAGTGCCGGGCCTT	42
ACAGGTCGTAAAGATATAAGCAAATATTACGACGTATTTCAC	42
CCTTTAACAATGCCTAAACGTTAATATTAACGCCACGAACTC	42
AAGAGGTAACCCTCAATTCGCATTAAATTGCAAGGGTGTAAT	42
GCTTAGACGCAAGGAAATCAGCTCATTTGCTGGCGTAAGCTA	42
TATAATGCGGGAGAAATAGGAACGCCATGGCCTCTCCTCGAT	42
GTTTTAATATGACCAATTCGCGTCTGGCTTGGGAACCCGGGT	42
TACGGTGAATCGGTTAGCCAGCTTTCATGCCATTCCGTAATC	42
TCCATATAAGCCTCAAATGTGAGCGAGTAAACCAGTTCCTGT	42
AATTCTGAATTAGCCGTCGGATTCTCCGCGGCACCCGCTCAC	42
TAGTTTGAATCATAAAACGGCGGATTGAATCGCACTACGAGC	42
TTCGCAATAGTAGCGGGATAGGTCACGTGACAGTAGTAAAGC	42
GTTTAGCTGGCATCGATGGGCGCATCGTAACCGCAGCGAGCT	42
GGCACCCTGAACAACGCCACCAGAACTTTTGCGGACACCGGA	42
GGGTAATCGCATTATTAGTATCATATGCGATAAATGCGGAAT	42
AGAGAGAAATAACAAAATTCTTACCAGTGGTTTGATGATTAT	42
TGAGTTAAATAGCACAACGCTCAACAGTATCTTCTTCATCAA	42
AGCAAGATTGTTTAAATTGAGAATCGCCAAATATATTTGGAT	42
AATAGCTATCCAAAACAACGCCAACATGAGAACGCATGGAAG	42
CTTTTTAACAGCCAGGCAGAGGCATTTTGCAAATCATATCAA	42
ATAGCCGAATTTGCGTAATAAGAGAATATAACTATAAAACAG	42
AAGGAAAACGAGCGCCGACAAAAGGTAATCCGGCTCGTAGAT	42
TAATAACTCCTGAACTGTCCAGACGACGTGAGAGATCAGATG	42
AACTGGCTTGCACCACAACATGTTCAGCAATTTATGTACCTT	42
TTATTACAAATCAAGAACGCGCCTGTTTCGCTGAGACAATAA	42
AACGTAGTGCGGGAATAGATAAGTCCTGTAGCGATTGCTTTG	42
AAGGTGGGTTTTAGAAAATAATATCCCACCCTTAGAAATCGC	42
AACGCAAGGTATTCTTACGAGCATGTAGTCGTCGCTTCATTT	42
AAGTTTATCAGATATCAATAATCGGCTGGAATAACAAAGAAG	42
ATAGAAACCGCGCCTTATCATTCCAAGACATAAATATCAAGA	42
TGGTTTACCAGCGCCAAAGACAAAAGGGCGACATTCA	37
ACCATAAATCAAAAATCAGGTCTTTACCCTGAC	33
ACCGATTGAGGGAGGGAAGGTAAATATTGACGGAAAT	37
GAGATTTGTATCATCGCCTGA	21
TAAATTGTGTCGAAATCCGGAGAATGGAGTGACCTTAAGT	40
TTACTTAGCTTTAAAGTGCGGGTTGTGAATTCATGCGTAAAAAGCTGAT	49
CAGACGGTTCATTGAGCAACTGGGTGGATGTTCTTATATGTAAATATGA	49
ACCGAACACTGCGGAATATAGAAGCTTTCTCAGGACAGAAAAGGAGACA	49
AGAGGACTTAGACTCTGACGCTGTAAAACGACGGCAAGATTGTTCAAAA	49
CAGACCAGAGGGGGATTTCTCGGGTTTTCCCAGTCTAAATTGTGAGTAA	49
CTGACCTTTTGCAACTGGTTGCGATTAAGTTGGGTTTGTTAAATATATT	49
CTTGACAAAACCAACAGGGCTAAAGGGGGATGTGCTTTTGTTATAAAAA	49

ATTACCCTCGTTTACTTGTTATCGCTATTACGCCATTTAACCAGCCTTT	49
AAGCTGCAGAGCAAGAGGATCGGGCGATCGGTGCGCAAAAATCTGTAAT	49
AGGCTTGAAGGAATTCGAATTAGGCTGCGCAACTGCTTCCTGTGTACCA	49
ACCAGAAAATGCAGTAGCTGTGCAAAGCGCCATTCCAACATTAGAGCAT	49
GGCTTGATTAGGAATGTTATCGCTTCTGGTGCCGGAACAACCAAAATTA	49
AACTTTAGAAAGATCACAACATCCAGCCAGCTTTCTGGGAACCAGGCAA	49
CCTTATGGGAACAAATAAAGTTCGGCCTCAGGAAGCCGTAATATTAACA	49
GGCTCATACGTTAAGCCTAATTTTGAGGGGACGACTGGTGTAAATTCTA	49
AGCATTGACCACCCAGAAGGAAAGGCGTTAAATAAAGCCTGTGACGGGA	49
CAGGTCAAGAACCGATATTCCAATACCGACCGTGTGTTATACTAAAAAC	49
ATATTCATCCCTCATGGCAATGACCTAAATTTAATATAAAGCGCCTTTA	49
CTCATTAAGAGCCACTGATTGTTTTAGTTAATTTCAGGGCTTACGTCAA	49
AGCGCAGATAATCACTGAATAGAGAAAACTTTTTCATATTTATAAGAAA	49
GTTCCAGATTAGCGACCTACCCAATCGCAAGACAATAATTTATATTATT	49
GGCTTTTATTTTCGTGCACGTATGTAAATGCTGATCGAGCCACAGTTAC	49
GTACTGGCTGTAGCGAAATTGTAGGTTGGGTTATATAAAGTATCTTTCC	49
GGGGTCAAAGTTTGTTTAACGCTACCTTTTTAACCAGTAATTTCTTACC	49
AGTGCCCGTAATCAAGTAACACAAAATCATAGGTCACAATAACAGCTAC	49
TGCCCCCGAAACCAGGGAGAAAAGAGTCAATAGTGTAATGCAGATTAGT	49
CCTATTAAAGGCCGGCCTGATAGCTTAGATTAAGAATCAACAGGTTTTG	49
AGTATTAAGTAGCAAGTTACAAATCCTTGAAAACAAACAAGACGAACCT	49
TCAAGAATTAGAGCGAATTATATTAATTAATTTTTCCTAATTAAGAAC	48
AGCGGGGACCGACTCTGAGCACTTGCTTCTGTAAAAAACCAATAGAAGG	49
AGGCGGATAAAGGTAACAAACCAATATATGTGAGTTCTTTCCCAATAGC	49
CGTCGAGAGGGTTGATATAAGTATAGCCCGGAATAGG	37
GCGATTATACCAAGCGCGAAACAAAGTACAACG	33
ACCCCCACAAGTTTCACTATT	21
ACACTAAGCCGTAATTCCAGTTTGGAACTACCGACACAGTTCAGCCCGA	49
AAACGAAACCCTAACCGAGATAGGGTTGATCTGTAAATCCCCCGCGTTT	49
CACTACGTTAGAGCCTTATAAATCAAAAGGGCACGAATCGTCAAGCGAA	49
AACGGGTCGGCGAAGTTCCGAAATCGGCGAATCGGGGATAGCAACTCCA	49
TTCATGAAAGGGAACGAAAATCCTGTTTATAAATCTAATAGTGAGAGTA	49
GCTTTGACGGGCGCACGCTGGTTTGCCCTGACCTCAAGAAGTTTTTGAT	49
AGGGTAGGTGTAGCGAGAGTTGCAGCAAGAGTAAAAATAGCGGCGGATG	49
GCGAAAGTAACCACCCCTTCACCGCCTGCGTGGTGCCAGACGTGCTGAA	49
GCGGGATTTAATGCGAGACGGGCAACAGAAAGACGCACTATCTCAACAT	49
AGGGAGTCGTACTAGTGGTTTTTCTTTTACCGAGCTACGAGGACTAAAG	49
TATTCGGAGCACGTGTTTGCGTATTGGGATGGTCAATACATAGTTTCAT	49
ATCGCCCTCGTTAGGCCAACGCGCGGGGGTGAAATTACCACAGATTCCC	49
GCGCCGACTAAACAGCCAGCTGCATTAAAATTCCATCATCAGGTAGATT	49
AACAGCTGGGATTTTCCAGTCGGGAAACCGGAAGCCATTATTGATACAT	49
GCTTTCGACGCCAGCGTTGCGCTCACTGCTGGGGTTAAAACGATAACCT	49

ATTGTATTTTTTATACATTATCATCACATTAATTGAATCCTG	42
AGGCTCCCCGAGTATTAATTTTAAAAGTAACCACCTCAGAGCAGTCAGA	49
CGTTGAATCACGCAAAATCCTTTGCCCGTATCATCCCACCCTTAATATC	49
TTGCGAAGCAATACAACAATTCGACAACCAGATGAGAGCCGCCAAGAAT	49
AGAAAGGATAACATTTAGAAGTATTAGATATAATCCCACCGGTAATAAG	49
CAGTTTCGAAGAACATAGATAATACATTTATACTTAAATCACAAATAGC	49
ATTTTGCTTGCTGGACTAATAGATTAGAGGTTAGATTTGCCACGAAGCC	49
TAAATGAATATTACATATCTTTAGGAGCAATTATTGTCATAGTAAGCAG	49
TGTCGTCACAGGAAAATTGAGGAAGGTTAAATAAAGCGTTTTTTACCAG	49
TAGCGTAATACCTAGGCAAATCAACAGTTTTCAGGCCTTTAGAACGCAA	49
TCCACAGATCGTCTTCAATCAATATCTGAATATACGTAGCGACCAAAAG	49
AAACTACACATTGGTGAACCTCAAATATTTACATCTCGATAGAGACTCC	49
ACTGAGTACACGACAAATCTAAAGCATCCGGATTCGAAACGTGTTAGCA	49
TAGGAACACATTCTTGAGAGCCAGCAGCAATACCACCATTACATACATA	49
TTTCAGGGAACCCTCCGCCTGCAACAGTGCAGAGGCCAGCAATAAAAGA	49
CCTCAGAGTAAGAAGTGAGGCGGTCAGTCAATTACTGAGCCAACGGAAT	49
CGCCACCAATATTTCCAGCAGAAGATAAATGATGAGAATTATACAATCA	49
TGTATCACCGTACTCAGGAGGTTTAGTACCGCCACCC	37
object-label:	
Cy5-TTTATCAGGGCGATGGCCCACTACGTGAACCATCACCCAAAT	42

Supplementary Table 7: list of oligonucleotide-sequences of the 10-helix bundle.

sequence	length	defect variant
TCCACAGATCGTCTTCAATCAATATCTGAATATACGTAGCGACCAAAAG	49	3
ACTGAGTACACGACAAATCTAAAGCATCCGGATTCGAAACGTGTTAGCA	49	3
TTTCAGGGAACCCTCCGCCTGCAACAGTGCAGAGGCCAGCAATAAAAGA	49	3
ATTTTGCTTGCTGGACTAATAGATTAGAGGTTAGATTTGCCACGAAGCC	49	5
TGTCGTCACAGGAAAATTGAGGAAGGTTAAATAAAGCGTTTTTTACCAG	49	5
GCGCCGACTAAACAGCCAGCTGCATTAAAATTCCATCATCAGGTAGATT	49	8
CGTTGAATCACGCAAAATCCTTTGCCCGTATCATCCCACCCTTAATATC	49	8
AGAAAGGATAACATTTAGAAGTATTAGATATAATCCCACCGGTAATAAG	49	8
GCGGGATTTAATGCGAGACGGGCAACAGAAAGACGCACTATCTCAACAT	49	10
TATTCGGAGCACGTGTTTGCGTATTGGGATGGTCAATACATAGTTTCAT	49	10
AGTGCCCGTAATCAAGTAACACAAAATCATAGGTCACAATAACAGCTAC	49	15
CCTATTAAAGGCCGGCCTGATAGCTTAGATTAAGAATCAACAGGTTTTG	49	15
CACTACGTTAGAGCCTTATAAATCAAAAGGGCACGAATCGTCAAGCGAA	49	15
TTCATGAAAGGGAACGAAAATCCTGTTTATAAATCTAATAGTGAGAGTA	49	15
AGGGTAGGTGTAGCGAGAGTTGCAGCAAGAGTAAAAATAGCGGCGGATG	49	15
AACTTTAGAAAGATCACAACATCCAGCCAGCTTTCTGGGAACCAGGCAA	49	20
CAGGTCAAGAACCGATATTCCAATACCGACCGTGTGTTATACTAAAAAC	49	20
CTCATTAAGAGCCACTGATTGTTTTAGTTAATTTCAGGGCTTACGTCAA	49	20
GTTCCAGATTAGCGACCTACCCAATCGCAAGACAATAATTTATATTATT	49	20
GTACTGGCTGTAGCGAAATTGTAGGTTGGGTTATATAAAGTATCTTTCC	49	20
CAGTTTCGAAGAACATAGATAATACATTTATACTTAAATCACAAATAGC	49	30
TAAATGAATATTACATATCTTTAGGAGCAATTATTGTCATAGTAAGCAG	49	30
TAGCGTAATACCTAGGCAAATCAACAGTTTTCAGGCCTTTAGAACGCAA	49	30
AAACTACACATTGGTGAACCTCAAATATTTACATCTCGATAGAGACTCC	49	30
TAGGAACACATTCTTGAGAGCCAGCAGCAATACCACCATTACATACATA	49	30
ACCGAACACTGCGGAATATAGAAGCTTTCTCAGGACAGAAAAGGAGACA	49	30
CAGACCAGAGGGGGATTTCTCGGGTTTTCCCAGTCTAAATTGTGAGTAA	49	30
CTTGACAAAACCAACAGGGCTAAAGGGGGATGTGCTTTTGTTATAAAAA	49	30
AAGCTGCAGAGCAAGAGGATCGGGCGATCGGTGCGCAAAAATCTGTAAT	49	30
ACCAGAAAATGCAGTAGCTGTGCAAAGCGCCATTCCAACATTAGAGCAT	49	30
TGCCCCCGAAACCAGGGAGAAAAGAGTCAATAGTGTAATGCAGATTAGT	49	40
AGTATTAAGTAGCAAGTTACAAATCCTTGAAAACAAACAAGACGAACCT	49	40
AAACGAAACCCTAACCGAGATAGGGTTGATCTGTAAATCCCCCGCGTTT	49	40
AACGGGTCGGCGAAGTTCCGAAATCGGCGAATCGGGGATAGCAACTCCA	49	40
GCTTTGACGGGCGCACGCTGGTTTGCCCTGACCTCAAGAAGTTTTTGAT	49	40
GCGAAAGTAACCACCCCTTCACCGCCTGCGTGGTGCCAGACGTGCTGAA	49	40
AGGGAGTCGTACTAGTGGTTTTTCTTTTACCGAGCTACGAGGACTAAAG	49	40
ATCGCCCTCGTTAGGCCAACGCGCGGGGGTGAAATTACCACAGATTCCC	49	40
AACAGCTGGGATTTTCCAGTCGGGAAACCGGAAGCCATTATTGATACAT	49	40
TTGCGAAGCAATACAACAATTCGACAACCAGATGAGAGCCGCCAAGAAT	49	40
AGAGGACTTAGACTCTGACGCTGTAAAACGACGGCAAGATTGTTCAAAA	49	50

CTGACCTTTTGCAACTGGTTGCGATTAAGTTGGGTTTGTTAAATATATT	49	50
ATTACCCTCGTTTACTTGTTATCGCTATTACGCCATTTAACCAGCCTTT	49	50
AGGCTTGAAGGAATTCGAATTAGGCTGCGCAACTGCTTCCTGTGTACCA	49	50
GGCTTGATTAGGAATGTTATCGCTTCTGGTGCCGGAACAACCAAAATTA	49	50
CCTTATGGGAACAAATAAAGTTCGGCCTCAGGAAGCCGTAATATTAACA	49	50
ATATTCATCCCTCATGGCAATGACCTAAATTTAATATAAAGCGCCTTTA	49	50
AGCGCAGATAATCACTGAATAGAGAAAACTTTTTCATATTTATAAGAAA	49	50
GGCTTTTATTTTCGTGCACGTATGTAAATGCTGATCGAGCCACAGTTAC	49	50
GGGGTCAAAGTTTGTTTAACGCTACCTTTTTAACCAGTAATTTCTTACC	49	50

Supplementary Table 8: list of omitted oligonucleotides for the pseudo-defect-variants of the 10-helix-bundle.

Sequence	Length
GGGCGAAAAACCGTCTATCAGGGCGATGGCCCACTAC	37
CGTCAAAGTGCCGTTATTTACCTGAAAGGATTGTTAGAAATT	42
ATTAAAGGAACCCTGCTCAATGCACAGAGCAATTCGTTTAAC	42
AGTTTGGATTTAGATGGAAATGGCTATTATTCCTGCAGTAAC	42
GATAGGGGCCGGCGTTGCAACGAACTGAAAGGAGCCGGGAGA	42
TAAATCAGGAAGGGGAACAATCGCCATTGCGGAACCGCCTGA	42
CGAAATCAGCGGGCCGGCCTTAACCACCTTTGAGTAAGTTAC	42
AATCCTGAAGTGTAGAGTAGACAGAGGTGAACGTTGCGAATT	42
TGGTTTGCGTAACCTAGTAATTAACACCCTCGTATCCTGAGC	42
GTTGCAGGCTTAATTTGTAGCCACGCTGACTTTACAAACAAA	42
TCACCGCCGCGTACGTCCATCATGAAAATTGAGGAAATTAAT	42
CGGGCAACGAGCACGGCCACCCTTGCTGAGCCGTCTCATTTG	42
TTTTTCTCCTCGTTAAGTGTTAACCCTCCACTAACATGGAAA	42
GCGTATTAGCTAAAACGGTACCAGTTGGTATCTAAATATATG	42
ACGCGCGAACAAGGCGATTAAAGTAAATCACCAGAGCCGCCG	42
GCTGCATAACGCCACCAGCTGAATTAATCAGTCAATCAAATA	42
GTCGGGAACGACGTGCGGGCCCTATTTTAAGGGTGGAGCTTC	42
GCGCTCACAGTGCCCTGTTGGAGGCTATAATGTGTCGGAAGC	42
CTAACTCGAAGCCATTCGCCAAGAGTCTTTTTAAACAGGATT	42
CTGGGGTCTAAGTGCGGAAACAATCGATAATTTTTATTGCTC	42
CGGAAGCCGCACGATTCCGGCAAACTAGTTATTTCTCATTTT	42
AATTCCAGTGCTGAAAGATCGGTACCCCATACTTTAGCTTAA	42
GTGAAATGACAATGGACGACAAAAGCCCCAAAAACGCTGTAG	42
ATGGTCACCCCGCTCATCTGCTTGTATAATAAAGCAATATGC	42
ACCGAGCGCTCGCCGATGGGCTTGTAAATAAGCAAGTCTGGA	42
AAAGACGTGATACCGGGATAGTAAAATTAAGGCAATAACAGT	42
CGTGGTGGCCATCTAAACGGCGTTAAATCATCCAAGCGAACG	42
GTGTAATGAGTAAAGGTGGGCCGTCGGAACCAATATACTAATGACCATT	49
AAATCATTTCTCCGAACTCTGACCTCCTGGTTG	33
GTGAACCATCACCCAAATCAAGTTTTTTGGGGTCGAG	37
TAGGGGCCTTGAATCGGCTGACGCATTTCACAT	33
CAGTCACACGACCAGTAATAAAA	23
AATGGATAAAGCACGGAAGGTCATTACCATTAGCA	35
TTTTGACAAAGGGAACATTCAAAACGTCACCAATG	35
ACGCTCAGCTTGACAGCGCCACGATAGCAGCACCG	35
CCAGCCAAACGTGGAGAAAATTAGCGACAGAATCA	35
ATATCCAAAGAAAGGTTTATTCTTTAGCGTCAGAC	35
AAACTATGCTAGGGCGCAAAGCGTTTTCATCGGCA	35
CTTGCCTGCGGTCAGGTGGCATCATAGCCCCCTTA	35
CTTTGATACCACACCGTAGAATTGCCATCTTTTCA	35
TTAACCGGCGCCGCATTACGCAATCACCGGAACCA	35

AGAGTCTTATGGTTCTGGCATCACCGGAACCGCCT	35
TCAGTGAGTATAACATAACGGAGCCGCCACCCTCA	35
TCCTGAGAGAATCAGGAAACCCACCCTCAGAGCCA	35
GACAGGACAGGAGGAGCCGAACAGAGCCGCCACCA	35
GGGGATGTGCTGGGAATCAACCGTTCTAAGGACAGAAACAAC	42
TATTACGGGGTTTTTTTGTCGTGACAGGAGGTTGA	35
GATCGGTTGTAAAAGTTAGCGCAGACGATTGGCCT	35
TGCGCAAAAGCTTTATTCCACCACAAACAAATAAA	35
AGCGCCAGGGTGGAACAAACTTAAAGCCAGAATGG	35
CTGGTGCGTTGTGAACACTGAAGTCTCTGAATTTA	35
GCCAGCTCTTAAGTAATAGGAAGTAAGCGTCATAC	35
CCTCAGGATTGTCAATTTTCATTGATGATACAGGA	35
AGGGGACTCCCGCCACCCTCAGGTAATAAGTTTTA	35
AACCGTGTCTAATCACCGCCACAGTGCCTTGAGTA	35
TGGTGTACTGGAGTTTTAGTACCGTATAAACAGTT	35
CCGTAATGACAGTGTGTATCACCTGCCTATTTCGG	35
TGGGAACGTAAGCAATAAGTATATTCTGAAACATG	35
AGCGAGTAACAACCACGAATAAGTGCCGTAAGAGG	35
GCCAGCTTTCATCAACATTAAATGTG	26
GGGACATTCTGGCCAACAGAGATAGAACCC	30
CATCAAAAATAATTCGCGTCTGGCCTTCCTGTA	33
TACTTCTGAATAATGGAAGGGTTAGAACCT	30
GGTGGCATCAATTCGGAACGCAAAGACTGTCACCCTCAGCAG	42
TTTTCATTTGGGGCGCGAGCTGAAAA	26
GCGTAGAAAATTTATGCGTTATACAAATGTTGCTAATACGTGCGTCTGA	49
GTCAGATGTTAATTAGTATAAAGCCAACTGAAGCCTTTGAATACCTACA	49
AGTACCTAACTTTTAGTAGGGCTTAATTCTCCCGAAATGCGCAGGAAAA	49
AACAATAGCAAGACGCCATATTTAACAAACGCGAGAAAACATATTACCG	49
TTGCTTTAATGCTGATGTAATTTAGGCAGGCTTATCCGAACGGCTGGTA	49
AAAATCGTGGGTTATTTCGAGCCAGTAAGCAAGCAGATAAAAAGAACTC	49
ATTCATTTTTTTAAATATAAAGTACCGAGAATCATTCAGTATAACATCA	49
AAAAGAATCATAGGTAAAGTAATTCTGTCCGTTTTACAGTGCAATACTT	49
CATCAAGTCAATAGACGACAATAAACAACACTCATGCAGCAAACGCAAA	49
TACATTTAGATTAAAGCTAATGCAGAACCGGGTATGCATCACGAGTAAA	49
AATTACCTTGAAAATTTATCAACAATAGCTTTCCTAATATCATTTATAA	49
CAGTACAATTAATTCTGAACAAGAAAAAAACCAATATCTGGTGCCAGAA	49
TGAGTGAATAACATGAATTGAGGAAGGTCAAATCATACGAGCAGTAAGC	49
AAGCCCGGGATTGCATAGGCTGGCTGACTTGAAAGGCTGATAGCGAAAG	49
TCGCGTTTATTATAAAGAGTAATCTTGAAGGGAACAGGGTAGTCTTCGC	49
AAAGCGAAAATCAGCGGATATTCATTACAGGCGCATCTACAAGAAGGGC	49
AAACTCCACGAGAAAACGTAACAAAGCTCCATGTTTTGCCTGTTCAGGC	49
AGAGAGTATGCTTTCAGTGAATAAGGCTTGTCGAAACAAGAGCAGGCAA	49

CTTTTGATATTCATACGAGAAACACCAGGTATCATAATCGTAACCGCTT	49
TGCGGATATACTGCAGTAAATTGGGCTTAACAAAGATCATATCACTCCA	49
TTGCTGAGTTTAGATTTAATTTCAACTTCAGCGATAATCAGAGTATCGG	49
CTCAACACAGAGGGTGTGAATTACCTTAAAAACACAGGAAGACAGTTTG	49
AACTAAACTTTTGCTTAAGAACTGGCTCAAAGAGGATTTAAAGCATCGT	49
AGTTTCAAAAAACCCAGTCAGGACGTTGCGAAGGCATTTTGTGTCACGT	49
TGATTCCCCTCGTTAAATCTACGTTAATGTAAAATAATTTTTGGATTGA	49
AGTAGATTAAGAGCACTAACGGAACAACGAGGAAGTTTTTTATTCTCCG	49
AGATACAAAAAGGACAGGTAGAAAGATT	28
TTTCGCAAATGGTCAATAACCTGTTTAGCTATA	33
CGACCGTGTGATAAATAAGGCGTTAAATAAGAA	33
CTGACCTTTTTCAGATCAATATAATCCTCGTAAGATTTTGCAATCCCAA	49
TATTTTAGAATATAATTATCAGATGATGCAATATTTTAAATCGATTTTT	49
GCGAGAATTTACATGGAATTATCATCATAGTCTTTCTTGCGGAATGAAA	49
TCCAATCACGGATTAAAGAAACCACCAGTAGCCCTGCGTTTTAGAGAGA	49
ATATGTAGAATACCAACATTATCATTTTAAAAATACCGGTATGGGAAGC	49
CTTAGGTCGCAGAGATTAATTTTAAAAGAGCAGAAAATCAGAAATTAAC	49
GACTACCTCAATTATAAATCCTTTGCCCGAGGCGGTACCGCGAAAGTCA	49
ATCAAAAGATGATGAAACAATTCGACAAGCCTGCATATTTTCGCTAATA	49
AGAAGAGAAAACAATTTAGAAGTATTAGAGAGCCACGAGAACCACAAGA	49
ATAGCTTAACAATTAATAGATAATACATATCTAAATAAACCAAATAATA	49
AGAATCCTTTTTTAAACTAATAGATTAGAACCTCATATCATTTGAAATA	49
GCTATTATAAATCAAATATCTTTAGGAGAATCAATCAATAATACCGAAG	49
TTCTGTACCATCCTAACAGACCAGGCGCATCAAAAAGCTTGC	42
GCAAAGCAAAGACTATCACCATCAATATGATATAGTAAGAGG	42
CCCTGACTTAATTCAGAAAGGCCGGAGAGCCGGAGCGAACTGGCGGAGT	49
AAATCAAACCAGACAGGTAAAGATTCAATGAGAGAGACGGTCACAACTA	49
TCAGAAAAACAGGTTGCAATGCCTGAGTCAGGTCAACTTAGCAATAATT	49
CCCTCAAACCTTTAAGAACCCTCATATAGGAGCAAATCCGCGATCTCCA	49
TCATAAATAAGAGGAACGCAAGGATAAAGAACGGTCGCCTGAAAAGGAG	49
GCGTCCAGGCTTAGTGCGGGAGAAGCCTCATGTCATACAACGGGTTTAT	49
GTAAAATATATAATATTATGACCCTGTAGGTTGATTATACCAGGTGAAT	49
GTTTTGCTGTTTTATAAATCGGTTGTACCAAAAACTCATCTTGATACCG	49
CGAGAGGGTACGGTTAAAGCCTCAGAGCAGCAAATCAAAAGAAATGACA	49
CGACGATTTCCATAAGAATTAGCAAAATCGTTAATACCAACCCGCATAA	49
TCATAACCAATTCTTAAATCATACAGGCCGCATTAACGTAATCGCTGAG	49
GGCATAGTTAGTTTAGTAGTAGCATTAACAGCTCATTTCCATAAAGGCC	49
CACATTCAACTAATGCAGATACATAACGCC	30
TAAACACCGGAATCATAATTACTAGA	26
AAAAGCCTGTTTAGACAATTTTTCTGACATTGGCAGATTCAC	42
TATCATAATGGTTTGAAATACGAAATAATGGATTA	35
CATCAGTTGAGATTTAGGAATAC	23

TATCCTGAATCTTACCAACGCTAACGAGCGTCT	33
GTAGCAACGGCTACAGAGGCTTTGAGGACT	30
TTCCAGAGCCTAATTTGCCAGTTACAAAATAAACAGCCAT	40
TCCAAATGAGGGAGTAAATCGAACGTGGACTCCAA	35
TGTTTAAAAGGGCGGCCCCCGAACAAGAGTCCACT	35
ATAGCAGGTTTACCGGGGAAATTGAGTGTTGTTCC	35
ATAACATAATCAATCGAGAAAAAAGAATAGCCCGA	35
GCATTAGGGAATAACGAAAGGGGCAAAATCCCTTA	35
TGAACACAAAGAAACGCTGGCTTTGATGGTGGTTC	35
GAGGGTAACATAAACGCTGCGCCCCAGCAGGCGAA	35
TCAGAGATAGCAAACCGCCGCCAAGCGGTCCACGC	35
ATTGAGTACTCCTTTACAGGGCTGGCCCTGAGAGA	35
AGAGCAAAAAAGAAGCTTTGACAGCTGATTGCCCT	35
GCAATAGCGCAATAGTGCTTTTTTCACCAGTGAGA	35
CCCTTTTACCAGAAGAGCGGGGGGCGCCAGGGTGG	35
AGTGGGATTTTGCTATGAACGGTGTATTACAGTTGGATTTTA	42
TTTCAACAGACGTTGTTGGGTTAATGAATCGGCCA	35
GAGAATACTAAAGTCCCAGTCAACCTGTCGTGCCA	35
AAGGAATCCTCATACGACGGCCTGCCCGCTTTCCA	35
TTTTCACCTGTAGCCTCAGGAACATTAATTGCGTT	35
AAAAAAACACCAGTTGTTCTTGCCTAATGAGTGAG	35
CCTTTAATACCGTAATTCATGATAAAGTGTAAAGC	35
CAGCTTGCAAGCCCGTCCTTACACAACATACGAGC	35
TTCTTAACACCCTCACCTTATTGTTATCCGCTCAC	35
ATAGTTGAACCGCCAAAATAATAGCTGTTTCCTGT	35
ACAACCACCTCAGATATTTACTCGAATTCGTAATC	35
CCGATATCAGGAGGGACTCTAGAGGATCCCCGGGT	35
GCTTGCAGAATAGGCGGCCCTCTTGTTACCTCGAT	35
GCTTTTGGGTTGATACTCGTCCAGGGCTTAAGCTA	35
CGAAAGACAGCATCGGAACGAGG	23
GACGGAAATTATTCATTAAAGGTGAATTATCACCGTCACC	40
AGCGGGGTTTTGCTCAGTACCAGGCGGATA	30
GACTTGAGCCATTTGGGAATTAGAGCCAGCAAAATCACCA	40
GTAGCACAAATATTATTATTTCCCAGCT	28
AGGCCGGACCGATTAAGAAACAAGATTATCTTACCTCATCTT	42
AAACCATAAGACAACGTCAAAGAGGTTTGCTCAACTCAAATA	42
TAATCAGTCATATGCCTTTACAGCGAACGAGAATCAAAGAAC	42
AGTTTGCTTGTCACAAAAACATCTAAGACGCCAACATGCAAA	42
TGTAGCGACACCACACGGGAGTATAGAAGAGGCATTATAACT	42
TTTTCGGACATATACCTGAACCCCAATATAAGAGACCTCCGG	42
TTAGCGTAATACATATTGAGCATCGTAGCAAAAGGTCTGAGA	42
TAATCAAAGTATGTGATAACCAAGCAAGCCAGACGTGAATTT	42

GAGCCACGATTAAGTAAGCCCAGTACCGCATGTTCGACGCTG	42
CCCTCAGAATACCCGAAACAACCAAGAAGCGCCTGCATAGCG	42
GAACCGCGAGGAAACTATCTTCGGCTGTATAAGTCTTCCCTT	42
CCACCCTCAAAGTTTAAGAAAATGTAGATAATATCAATCGTC	42
GAACCACGAATTTTCTGTAATCCGATTAGGGAGAGGCGGTTT	42
CCAGCATTCTTTCCAGTTTCAACCAACTCTTCATCGTCAGAA	42
GGCAGGTTAACGATGAAAGGAAATCATACAAGAACGTCTTTA	42
TGATATTAGACAGCTGCGAATCGGAACGCCAAATCTGACCAT	42
TCCTCATACAACGCGTTGAAAACCTGCTGCTCATTAAACAGT	42
AAAGCGCGTTTCGTGGCTCCATAAATTGTGCCCTGTGAATCC	42
CCGTTCCACCCATGTTGTATCGAGATTTAACGAGTGGAATCG	42
ATGGCTTGGGATAGCTTTCGAAGCGCGAGAGATGGCTGGATA	42
GTGTACTGAGCCACACAGCTTTGACCCCTAATCATGGTAATA	42
ACGGGGTCCCTCAGCGCCGACATACACTTGCGATTAAAAGAA	42
ACAGTGCCCGCCACTCGCCCATAAAACGATTATACAAAATAG	42
AATGCCCCCGTACTATTCGGTGCCACTAGGAAGAATACCAGA	42
AACCTATTAGCCCGGGGAGTTTAAACGGAAAACGAAACACTA	42
AAAGTATTCGAGAGCGGGATCTTTTCATATTATTAATTACGA	42
CTGAGACTCCTCAAGAGAAGGATTAGGATT	30
object-label:	
Cy5-TTACCATATCAAAATTATTTGCACGTAAAACA	

Supplementary Table 9: list of oligonucleotide-sequences of the 12-helix bundle.

sequence	length	defect variant
GTAAAATATATAATATTATGACCCTGTAGGTTGATTATACCAGGTGAAT	49	3
CGAGAGGGTACGGTTAAAGCCTCAGAGCAGCAAATCAAAAGAAATGACA	49	3
TCATAACCAATTCTTAAATCATACAGGCCGCATTAACGTAATCGCTGAG	49	3
TCAGAAAAACAGGTTGCAATGCCTGAGTCAGGTCAACTTAGCAATAATT	49	5
TCATAAATAAGAGGAACGCAAGGATAAAGAACGGTCGCCTGAAAAGGAG	49	5
AGAAGAGAAAACAATTTAGAAGTATTAGAGAGCCACGAGAACCACAAGA	49	8
AGAATCCTTTTTTAAACTAATAGATTAGAACCTCATATCATTTGAAATA	49	8
CCCTGACTTAATTCAGAAAGGCCGGAGAGCCGGAGCGAACTGGCGGAGT	49	8
AACTAAACTTTTGCTTAAGAACTGGCTCAAAGAGGATTTAAAGCATCGT	49	10
TGATTCCCCTCGTTAAATCTACGTTAATGTAAAATAATTTTTGGATTGA	49	10
AATTACCTTGAAAATTTATCAACAATAGCTTTCCTAATATCATTTATAA	49	15
TCGCGTTTATTATAAAGAGTAATCTTGAAGGGAACAGGGTAGTCTTCGC	49	15
AAACTCCACGAGAAAACGTAACAAAGCTCCATGTTTTGCCTGTTCAGGC	49	15
CTTTTGATATTCATACGAGAAACACCAGGTATCATAATCGTAACCGCTT	49	15
TTGCTGAGTTTAGATTTAATTTCAACTTCAGCGATAATCAGAGTATCGG	49	15
ATTCATTTTTTTAAATATAAAGTACCGAGAATCATTCAGTATAACATCA	49	20
GCGAGAATTTACATGGAATTATCATCATAGTCTTTCTTGCGGAATGAAA	49	20
ATATGTAGAATACCAACATTATCATTTTAAAAATACCGGTATGGGAAGC	49	20
GACTACCTCAATTATAAATCCTTTGCCCGAGGCGGTACCGCGAAAGTCA	49	20
CATCAAGTCAATAGACGACAATAAACAACACTCATGCAGCAAACGCAAA	49	20
ATCAAAAGATGATGAAACAATTCGACAAGCCTGCATATTTTCGCTAATA	49	30
ATAGCTTAACAATTAATAGATAATACATATCTAAATAAACCAAATAATA	49	30
GCTATTATAAATCAAATATCTTTAGGAGAATCAATCAATAATACCGAAG	49	30
AAATCAAACCAGACAGGTAAAGATTCAATGAGAGAGACGGTCACAACTA	49	30
CCCTCAAACCTTTAAGAACCCTCATATAGGAGCAAATCCGCGATCTCCA	49	30
GCGTCCAGGCTTAGTGCGGGAGAAGCCTCATGTCATACAACGGGTTTAT	49	30
GTTTTGCTGTTTTATAAATCGGTTGTACCAAAAACTCATCTTGATACCG	49	30
CGACGATTTCCATAAGAATTAGCAAAATCGTTAATACCAACCCGCATAA	49	30
AGTACCTAACTTTTAGTAGGGCTTAATTCTCCCGAAATGCGCAGGAAAA	49	30
TTGCTTTAATGCTGATGTAATTTAGGCAGGCTTATCCGAACGGCTGGTA	49	30
TCCAATCACGGATTAAAGAAACCACCAGTAGCCCTGCGTTTTAGAGAGA	49	40
CTTAGGTCGCAGAGATTAATTTTAAAAGAGCAGAAAATCAGAAATTAAC	49	40
AAAAGAATCATAGGTAAAGTAATTCTGTCCGTTTTACAGTGCAATACTT	49	40
TACATTTAGATTAAAGCTAATGCAGAACCGGGTATGCATCACGAGTAAA	49	40
CAGTACAATTAATTCTGAACAAGAAAAAAACCAATATCTGGTGCCAGAA	49	40
AAAGCGAAAATCAGCGGATATTCATTACAGGCGCATCTACAAGAAGGGC	49	40
AGAGAGTATGCTTTCAGTGAATAAGGCTTGTCGAAACAAGAGCAGGCAA	49	40
TGCGGATATACTGCAGTAAATTGGGCTTAACAAAGATCATATCACTCCA	49	40
CTCAACACAGAGGGTGTGAATTACCTTAAAAACACAGGAAGACAGTTTG	49	40
AGTTTCAAAAAACCCAGTCAGGACGTTGCGAAGGCATTTTGTGTCACGT	49	40
GCGTAGAAAATTTATGCGTTATACAAATGTTGCTAATACGTGCGTCTGA	49	50

TGAGTGAATAACATGAATTGAGGAAGGTCAAATCATACGAGCAGTAAGC	49	50
AAGCCCGGGATTGCATAGGCTGGCTGACTTGAAAGGCTGATAGCGAAAG	49	50
AGTAGATTAAGAGCACTAACGGAACAACGAGGAAGTTTTTTATTCTCCG	49	50
CTGACCTTTTTCAGATCAATATAATCCTCGTAAGATTTTGCAATCCCAA	49	50
AAATAAACAGCCATGACGGAAGGTCGAGGGGCGAAAAACCGTCTATCAG	49	50
GTCAGATGTTAATTAGTATAAAGCCAACTGAAGCCTTTGAATACCTACA	49	50
AACAATAGCAAGACGCCATATTTAACAAACGCGAGAAAACATATTACCG	49	50
AAAATCGTGGGTTATTTCGAGCCAGTAAGCAAGCAGATAAAAAGAACTC	49	50
TATTTTAGAATATAATTATCAGATGATGCAATATTTTAAATCGATTTTT	49	50

Supplementary Table 10: list of omitted oligonucleotides for the pseudo-defect-variants of the 12-helix-bundle.

Sequence	Length
GGGCGAAAAACCGTCTATCAGGGCGATGGCCCACTAC	37
CGTCAAAGTGCCGTTATTTACCTGAAAGGATTGTTAGAAATT	42
ATTAAAGGAACCCTGCTCAATGCACAGAGCAATTCGTTTAAC	42
AGTTTGGATTTAGATGGAAATGGCTATTATTCCTGCAGTAAC	42
GATAGGGGCCGGCGTTGCAACGAACTGAAAGGAGCCGGGAGA	42
TAAATCAGGAAGGGGAACAATCGCCATTGCGGAACCGCCTGA	42
CGAAATCAGCGGGCCGGCCTTAACCACCTTTGAGTAAGTTAC	42
AATCCTGAAGTGTAGAGTAGACAGAGGTGAACGTTGCGAATT	42
TGGTTTGCGTAACCTAGTAATTAACACCCTCGTATCCTGAGC	42
GTTGCAGGCTTAATTTGTAGCCACGCTGACTTTACAAACAAA	42
TCACCGCCGCGTACGTCCATCATGAAAATTGAGGAAATTAAT	42
CGGGCAACGAGCACGGCCACCCTTGCTGAGCCGTCTCATTTG	42
TTTTTCTCCTCGTTAAGTGTTAACCCTCCACTAACATGGAAA	42
GCGTATTAGCTAAAACGGTACCAGTTGGTATCTAAATATATG	42
ACGCGCGAACAAGGCGATTAAAGTAAATCACCAGAGCCGCCG	42
GCTGCATAACGCCACCAGCTGAATTAATCAGTCAATCAAATA	42
GTCGGGAACGACGTGCGGGCCCTATTTTAAGGGTGGAGCTTC	42
GCGCTCACAGTGCCCTGTTGGAGGCTATAATGTGTCGGAAGC	42
CTAACTCGAAGCCATTCGCCAAGAGTCTTTTTAAACAGGATT	42
CTGGGGTCTAAGTGCGGAAACAATCGATAATTTTTATTGCTC	42
CGGAAGCCGCACGATTCCGGCAAACTAGTTATTTCTCATTTT	42
AATTCCAGTGCTGAAAGATCGGTACCCCATACTTTAGCTTAA	42
GTGAAATGACAATGGACGACAAAAGCCCCAAAAACGCTGTAG	42
ATGGTCACCCCGCTCATCTGCTTGTATAATAAAGCAATATGC	42
ACCGAGCGCTCGCCGATGGGCTTGTAAATAAGCAAGTCTGGA	42
AAAGACGTGATACCGGGATAGTAAAATTAAGGCAATAACAGT	42
CGTGGTGGCCATCTAAACGGCGTTAAATCATCCAAGCGAACG	42
GTGTAATGAGTAAAGGTGGGCCGTCGGAACCAATATACTAATGACCATT	49
AAATCATTTCTCCGAACTCTGACCTCCTGGTTG	33
GTGAACCATCACCCAAATCAAGTTTTTTGGGGTCGAG	37
TAGGGGCCTTGAATCGGCTGACGCATTTCACAT	33
CAGTCACACGACCAGTAATAAAA	23
AATGGATAAAGCACGGAAGGTCATTACCATTAGCA	35
TTTTGACAAAGGGAACATTCAAAACGTCACCAATG	35
ACGCTCAGCTTGACAGCGCCACGATAGCAGCACCG	35
CCAGCCAAACGTGGAGAAAATTAGCGACAGAATCA	35
ATATCCAAAGAAAGGTTTATTCTTTAGCGTCAGAC	35
AAACTATGCTAGGGCGCAAAGCGTTTTCATCGGCA	35
CTTGCCTGCGGTCAGGTGGCATCATAGCCCCCTTA	35
CTTTGATACCACACCGTAGAATTGCCATCTTTTCA	35
TTAACCGGCGCCGCATTACGCAATCACCGGAACCA	35

AGAGTCTTATGGTTCTGGCATCACCGGAACCGCCT	35
TCAGTGAGTATAACATAACGGAGCCGCCACCCTCA	35
TCCTGAGAGAATCAGGAAACCCACCCTCAGAGCCA	35
GACAGGACAGGAGGAGCCGAACAGAGCCGCCACCA	35
GGGGATGTGCTGGGAATCAACCGTTCTAAGGACAGAAACAAC	42
TATTACGGGGTTTTTTTGTCGTGACAGGAGGTTGA	35
GATCGGTTGTAAAAGTTAGCGCAGACGATTGGCCT	35
TGCGCAAAAGCTTTATTCCACCACAAACAAATAAA	35
AGCGCCAGGGTGGAACAAACTTAAAGCCAGAATGG	35
CTGGTGCGTTGTGAACACTGAAGTCTCTGAATTTA	35
GCCAGCTCTTAAGTAATAGGAAGTAAGCGTCATAC	35
CCTCAGGATTGTCAATTTTCATTGATGATACAGGA	35
AGGGGACTCCCGCCACCCTCAGGTAATAAGTTTTA	35
AACCGTGTCTAATCACCGCCACAGTGCCTTGAGTA	35
TGGTGTACTGGAGTTTTAGTACCGTATAAACAGTT	35
CCGTAATGACAGTGTGTATCACCTGCCTATTTCGG	35
TGGGAACGTAAGCAATAAGTATATTCTGAAACATG	35
AGCGAGTAACAACCACGAATAAGTGCCGTAAGAGG	35
GCCAGCTTTCATCAACATTAAATGTG	26
GGGACATTCTGGCCAACAGAGATAGAACCC	30
CATCAAAAATAATTCGCGTCTGGCCTTCCTGTA	33
TACTTCTGAATAATGGAAGGGTTAGAACCT	30
GGTGGCATCAATTCGGAACGCAAAGACTGTCACCCTCAGCAG	42
TTTTCATTTGGGGCGCGAGCTGAAAA	26
GCGTAGAAAATTTATGCGTTATACAAATGTTGCTAATACGTGCGTCTGA	49
GTCAGATGTTAATTAGTATAAAGCCAACTGAAGCCTTTGAATACCTACA	49
AGTACCTAACTTTTAGTAGGGCTTAATTCTCCCGAAATGCGCAGGAAAA	49
AACAATAGCAAGACGCCATATTTAACAAACGCGAGAAAACATATTACCG	49
TTGCTTTAATGCTGATGTAATTTAGGCAGGCTTATCCGAACGGCTGGTA	49
AAAATCGTGGGTTATTTCGAGCCAGTAAGCAAGCAGATAAAAAGAACTC	49
ATTCATTTTTTTAAATATAAAGTACCGAGAATCATTCAGTATAACATCA	49
AAAAGAATCATAGGTAAAGTAATTCTGTCCGTTTTACAGTGCAATACTT	49
CATCAAGTCAATAGACGACAATAAACAACACTCATGCAGCAAACGCAAA	49
TACATTTAGATTAAAGCTAATGCAGAACCGGGTATGCATCACGAGTAAA	49
AATTACCTTGAAAATTTATCAACAATAGCTTTCCTAATATCATTTATAA	49
CAGTACAATTAATTCTGAACAAGAAAAAAACCAATATCTGGTGCCAGAA	49
TGAGTGAATAACATGAATTGAGGAAGGTCAAATCATACGAGCAGTAAGC	49
AAGCCCGGGATTGCATAGGCTGGCTGACTTGAAAGGCTGATAGCGAAAG	49
TCGCGTTTATTATAAAGAGTAATCTTGAAGGGAACAGGGTAGTCTTCGC	49
AAAGCGAAAATCAGCGGATATTCATTACAGGCGCATCTACAAGAAGGGC	49
AAACTCCACGAGAAAACGTAACAAAGCTCCATGTTTTGCCTGTTCAGGC	49
AGAGAGTATGCTTTCAGTGAATAAGGCTTGTCGAAACAAGAGCAGGCAA	49

CTTTTGATATTCATACGAGAAACACCAGGTATCATAATCGTAACCGCTT	49
TGCGGATATACTGCAGTAAATTGGGCTTAACAAAGATCATATCACTCCA	49
TTGCTGAGTTTAGATTTAATTTCAACTTCAGCGATAATCAGAGTATCGG	49
CTCAACACAGAGGGTGTGAATTACCTTAAAAACACAGGAAGACAGTTTG	49
AACTAAACTTTTGCTTAAGAACTGGCTCAAAGAGGATTTAAAGCATCGT	49
AGTTTCAAAAAACCCAGTCAGGACGTTGCGAAGGCATTTTGTGTCACGT	49
TGATTCCCCTCGTTAAATCTACGTTAATGTAAAATAATTTTTGGATTGA	49
AGTAGATTAAGAGCACTAACGGAACAACGAGGAAGTTTTTTATTCTCCG	49
AGATACAAAAAGGACAGGTAGAAAGATT	28
TTTCGCAAATGGTCAATAACCTGTTTAGCTATA	33
CGACCGTGTGATAAATAAGGCGTTAAATAAGAA	33
CTGACCTTTTTCAGATCAATATAATCCTCGTAAGATTTTGCAATCCCAA	49
TATTTTAGAATATAATTATCAGATGATGCAATATTTTAAATCGATTTTT	49
GCGAGAATTTACATGGAATTATCATCATAGTCTTTCTTGCGGAATGAAA	49
TCCAATCACGGATTAAAGAAACCACCAGTAGCCCTGCGTTTTAGAGAGA	49
ATATGTAGAATACCAACATTATCATTTTAAAAATACCGGTATGGGAAGC	49
CTTAGGTCGCAGAGATTAATTTTAAAAGAGCAGAAAATCAGAAATTAAC	49
GACTACCTCAATTATAAATCCTTTGCCCGAGGCGGTACCGCGAAAGTCA	49
ATCAAAAGATGATGAAACAATTCGACAAGCCTGCATATTTTCGCTAATA	49
AGAAGAGAAAACAATTTAGAAGTATTAGAGAGCCACGAGAACCACAAGA	49
ATAGCTTAACAATTAATAGATAATACATATCTAAATAAACCAAATAATA	49
AGAATCCTTTTTTAAACTAATAGATTAGAACCTCATATCATTTGAAATA	49
GCTATTATAAATCAAATATCTTTAGGAGAATCAATCAATAATACCGAAG	49
TTCTGTACCATCCTAACAGACCAGGCGCATCAAAAAGCTTGC	42
GCAAAGCAAAGACTATCACCATCAATATGATATAGTAAGAGG	42
CCCTGACTTAATTCAGAAAGGCCGGAGAGCCGGAGCGAACTGGCGGAGT	49
AAATCAAACCAGACAGGTAAAGATTCAATGAGAGAGACGGTCACAACTA	49
TCAGAAAAACAGGTTGCAATGCCTGAGTCAGGTCAACTTAGCAATAATT	49
CCCTCAAACCTTTAAGAACCCTCATATAGGAGCAAATCCGCGATCTCCA	49
TCATAAATAAGAGGAACGCAAGGATAAAGAACGGTCGCCTGAAAAGGAG	49
GCGTCCAGGCTTAGTGCGGGAGAAGCCTCATGTCATACAACGGGTTTAT	49
GTAAAATATATAATATTATGACCCTGTAGGTTGATTATACCAGGTGAAT	49
GTTTTGCTGTTTTATAAATCGGTTGTACCAAAAACTCATCTTGATACCG	49
CGAGAGGGTACGGTTAAAGCCTCAGAGCAGCAAATCAAAAGAAATGACA	49
CGACGATTTCCATAAGAATTAGCAAAATCGTTAATACCAACCCGCATAA	49
TCATAACCAATTCTTAAATCATACAGGCCGCATTAACGTAATCGCTGAG	49
GGCATAGTTAGTTTAGTAGTAGCATTAACAGCTCATTTCCATAAAGGCC	49
CACATTCAACTAATGCAGATACATAACGCC	30
TAAACACCGGAATCATAATTACTAGA	26
AAAAGCCTGTTTAGACAATTTTTCTGACATTGGCAGATTCAC	42
TATCATAATGGTTTGAAATACGAAATAATGGATTA	35
CATCAGTTGAGATTTAGGAATAC	23

TATCCTGAATCTTACCAACGCTAACGAGCGTCT	33
GTAGCAACGGCTACAGAGGCTTTGAGGACT	30
TTCCAGAGCCTAATTTGCCAGTTACAAAATAAACAGCCAT	40
TCCAAATGAGGGAGTAAATCGAACGTGGACTCCAA	35
TGTTTAAAAGGGCGGCCCCCGAACAAGAGTCCACT	35
ATAGCAGGTTTACCGGGGAAATTGAGTGTTGTTCC	35
ATAACATAATCAATCGAGAAAAAAGAATAGCCCGA	35
GCATTAGGGAATAACGAAAGGGGCAAAATCCCTTA	35
TGAACACAAAGAAACGCTGGCTTTGATGGTGGTTC	35
GAGGGTAACATAAACGCTGCGCCCCAGCAGGCGAA	35
TCAGAGATAGCAAACCGCCGCCAAGCGGTCCACGC	35
ATTGAGTACTCCTTTACAGGGCTGGCCCTGAGAGA	35
AGAGCAAAAAAGAAGCTTTGACAGCTGATTGCCCT	35
GCAATAGCGCAATAGTGCTTTTTTCACCAGTGAGA	35
CCCTTTTACCAGAAGAGCGGGGGGCGCCAGGGTGG	35
AGTGGGATTTTGCTATGAACGGTGTATTACAGTTGGATTTTA	42
TTTCAACAGACGTTGTTGGGTTAATGAATCGGCCA	35
GAGAATACTAAAGTCCCAGTCAACCTGTCGTGCCA	35
AAGGAATCCTCATACGACGGCCTGCCCGCTTTCCA	35
TTTTCACCTGTAGCCTCAGGAACATTAATTGCGTT	35
AAAAAAACACCAGTTGTTCTTGCCTAATGAGTGAG	35
CCTTTAATACCGTAATTCATGATAAAGTGTAAAGC	35
CAGCTTGCAAGCCCGTCCTTACACAACATACGAGC	35
TTCTTAACACCCTCACCTTATTGTTATCCGCTCAC	35
ATAGTTGAACCGCCAAAATAATAGCTGTTTCCTGT	35
ACAACCACCTCAGATATTTACTCGAATTCGTAATC	35
CCGATATCAGGAGGGACTCTAGAGGATCCCCGGGT	35
GCTTGCAGAATAGGCGGCCCTCTTGTTACCTCGAT	35
GCTTTTGGGTTGATACTCGTCCAGGGCTTAAGCTA	35
CGAAAGACAGCATCGGAACGAGG	23
GACGGAAATTATTCATTAAAGGTGAATTATCACCGTCACC	40
AGCGGGGTTTTGCTCAGTACCAGGCGGATA	30
GACTTGAGCCATTTGGGAATTAGAGCCAGCAAAATCACCA	40
GTAGCACAAATATTATTATTTCCCAGCT	28
AGGCCGGACCGATTAAGAAACAAGATTATCTTACCTCATCTT	42
AAACCATAAGACAACGTCAAAGAGGTTTGCTCAACTCAAATA	42
TAATCAGTCATATGCCTTTACAGCGAACGAGAATCAAAGAAC	42
AGTTTGCTTGTCACAAAAACATCTAAGACGCCAACATGCAAA	42
TGTAGCGACACCACACGGGAGTATAGAAGAGGCATTATAACT	42
TTTTCGGACATATACCTGAACCCCAATATAAGAGACCTCCGG	42
TTAGCGTAATACATATTGAGCATCGTAGCAAAAGGTCTGAGA	42
TAATCAAAGTATGTGATAACCAAGCAAGCCAGACGTGAATTT	42

GAGCCACGATTAAGTAAGCCCAGTACCGCATGTTCGACGCTG	42
CCCTCAGAATACCCGAAACAACCAAGAAGCGCCTGCATAGCG	42
GAACCGCGAGGAAACTATCTTCGGCTGTATAAGTCTTCCCTT	42
CCACCCTCAAAGTTTAAGAAAATGTAGATAATATCAATCGTC	42
GAACCACGAATTTTCTGTAATCCGATTAGGGAGAGGCGGTTT	42
CCAGCATTCTTTCCAGTTTCAACCAACTCTTCATCGTCAGAA	42
GGCAGGTTAACGATGAAAGGAAATCATACAAGAACGTCTTTA	42
TGATATTAGACAGCTGCGAATCGGAACGCCAAATCTGACCAT	42
TCCTCATACAACGCGTTGAAAACCTGCTGCTCATTAAACAGT	42
AAAGCGCGTTTCGTGGCTCCATAAATTGTGCCCTGTGAATCC	42
CCGTTCCACCCATGTTGTATCGAGATTTAACGAGTGGAATCG	42
ATGGCTTGGGATAGCTTTCGAAGCGCGAGAGATGGCTGGATA	42
GTGTACTGAGCCACACAGCTTTGACCCCTAATCATGGTAATA	42
ACGGGGTCCCTCAGCGCCGACATACACTTGCGATTAAAAGAA	42
ACAGTGCCCGCCACTCGCCCATAAAACGATTATACAAAATAG	42
AATGCCCCCGTACTATTCGGTGCCACTAGGAAGAATACCAGA	42
AACCTATTAGCCCGGGGAGTTTAAACGGAAAACGAAACACTA	42
AAAGTATTCGAGAGCGGGATCTTTTCATATTATTAATTACGA	42
CTGAGACTCCTCAAGAGAAGGATTAGGATT	30
object-label:	
Cy5-TTACCATATCAAAATTATTTGCACGTAAAACA	

Supplementary Table 11: list of sequences for the 42-helix bundle (“internal defects”-variants)

sequence	length	defect variant
TTATTAGTAGTAAAAAGATTA	21	1
GCATTTTGTTTAATAGCAAAG	21	2
GACTGTATTGTGAAACCCTGA	21	3
TCAAGTTTTTAAGATAAATCA	21	4
GTCAAAAATGAAAACCTAATTCTGTAATGGATTGA	35	5
GAGAGAAACAAGAAAGCCTTT	21	6
GGAAGCGTCAACAAATAAAAA	21	7
ATTAACTAATGCAGATATATT	21	8
AAGTCAGCAATAAATGAGTAATGTGTAGGTAAAACTGTCCAG	42	9
GATGATGAAACGGCACTTTTG	21	10
AAAACAACGTCGGAATTTCAA	21	11
AACAATTAAATGTGTTTTTAG	21	12
TTTTTTATAGCCAGTTAAATG	21	13
GGAAGGTTAAATCAAATTCGCGTCTGGC	28	14

Supplementary Table 12: list of omitted oligonucleotides for the 42-helix bundle structure - “internal-defects”-variant

sequence:	# bases	#As	#Ts	#Cs	#Gs		relative AT	relative GC
AGTATGTGAAACAAACCAGTAAATATATTTTAGTTAGATTAGTCTAAAG	49	21	16	4	8		0.755	0.245
AAGACAAAAAAACAAGGTAAAATTTATCAAAATCATCATTTTAGCCCTA	49	25	12	8	4		0.755	0.245
TGAGTAAAATCAGCTATTTTTAAATCAGGTCTTTAAGGAATTATCGTCA	49	17	18	6	8		0.714	0.286
TATTAAAAGGTGCCTTGCTGACACTAACAACTAATAATTTCATTATACA	49	19	16	9	5		0.714	0.286
AAATATTCGTCAAATTCAGCTAGCGATAGCTTAGATCATCATAATATTT	49	18	17	8	6		0.714	0.286
ATGAAACAGCTACACCAAGAATAACAATTTCATTTATAAAGAATCGTCT	49	22	13	9	5		0.714	0.286
TACATGGAACTACAATACACTTGCGATTTTAAGAATTTAATTCAAGGAT	49	19	16	7	7		0.714	0.286
ACGTCAAAAATCAAACCCTCATATCTAAAATATCTTTTAATGGCCTGTT	49	18	16	11	4		0.694	0.306
CCGTAATATTAGTTAGTACCGGAAAACAAAATTAATCAGGTTATACCTA	49	20	14	8	7		0.694	0.306
TGCGTATTTCCTCGAGAGATAGATTGTTTGGATTATGTAAATACAAGAA	49	16	17	5	11		0.673	0.327
TCATATGACGGGAGAGAATATGAGAGACTACCTTTTTAAAAGAAAATAC	49	21	12	6	10		0.673	0.327
ATTAAAGAAGAAACCTGTTTACCTTAGAATCCTTGGATGATGGTAAGAA	49	19	14	6	10		0.673	0.327
GAATTAGAGTTACAATCCCATAATAACCTTGCTTCTACTTCTACATTCT	49	15	18	12	4		0.673	0.327
CCAGTAGCTTTCCAATGTAGAATAAATCAATATATTTAGAACACACGAC	49	20	13	10	6		0.673	0.327
TCAAGTTGTTTTGAAAGCAAGAGATGATGAAACAATATACAGACAGGAA	49	22	11	5	11		0.673	0.327

Supplementary Table 13: list of oligonucleotides (49-bases length) of the 8-helix bundle structure with highest relative AT-content

sequence:	# bases	#As	#Ts	#Cs	#Gs		relative AT	relative GC
CGGAACCGCCACCCACCTGCTGCTCATTCAGTGAACGGTGTCTTAGCAA	49	11	10	17	11		0.429	0.571
CCAGAACGAAAGGACAGCGAATACATAACGCCAAACCCTGACCCGGAGA	49	20	3	15	11		0.469	0.531
TTGTCACCCTGAACCATTTTCCCGGCTTAGGTTGGTTTGCCCGCAGAAG	49	8	15	14	12		0.469	0.531
ATACCGAAACGGCGAAATGCAAAGCAAACTCCAACGACGTTGCGAAGGC	49	19	5	13	12		0.490	0.510
GCATTTTAAGAACGCCCAATAGCGCAGAGGCGAATGATTCGCTTGCTGG	49	13	11	11	14		0.490	0.510
CCGCCAGAGTTTCACGAGGGTACCACATTCAACTAGCAAAGCGTTCTAG	49	14	10	14	11		0.490	0.510
ACCGATTCCTTTACACGACGAGCTGAGAAGAGTCACCACCAGGTCTTTA	49	14	11	14	10		0.510	0.490
AACGCGCGGAGCTAAAAAGGGGAATAATGGAAGGGGTGAGTGCCTAATT	49	17	8	6	18		0.510	0.490
TCGCCCTGGATAGGTTTAGAAGATTAGAGAGTACCCTGGCTCAAAGAGG	49	14	11	9	15		0.510	0.490
CCTCCCTGGCTCCATATATTCCAGACGACGATAAACCCTCAAGAGCAAA	49	16	9	17	7		0.510	0.490
TGAATCGAGCCAGCCAAATCAGAGGAAGCCCGAAAAAAGATTGAGGACT	49	20	6	10	13		0.531	0.469
CAATGTCATCTGCCTTTTGCGTTTTTGCGGATGGCTCAACTTCAGCGAT	49	8	18	12	11		0.531	0.469
CCACCACTGCGAATTTGCGGGACGAGGCATAGTAAAAATCAAGAGAGAT	49	18	8	10	13		0.531	0.469
TGGAAAGAGCGTAAGCCACTAGGAAGAAAAATCTAAGACCGGATGCCTG	49	19	7	9	14		0.531	0.469
TTAACGGAGGAACCAGCGCGAGAGATGGTTTAATTTTAGAGCTGACCCT	49	14	12	9	14		0.531	0.469

Supplementary Table 14: list of oligonucleotides (49-bases length) of the 8-helix bundle structure with highest relative GC-content

Supplementary Note 1: Defect probe sequence design considerations.

The problem of detecting unknown single-stranded DNA sequence motifs has two aspects: the space of target sequences that will need to be detected, and the design of a probe for sampling (ideally) the entire target sequence space.

What is the space of potential target sequences that can realistically occur in assembled objects? Our primary goal is to detect unpaired DNA defects in objects made using the scaffolded DNA origami approach. We thus analyzed the sequence of the M13mp18-derived genomic DNA that is typically used in DNA origami for the occurrence of sequence strings of given lengths. Specifically, we counted how many times each of 4^N possible N-base long sequence strings are found in the scaffold DNA sequence and created histograms (i.e for N=2: how many times AA, AT, AG, AC etc pp). The resulting sequence statistics is shown in the figure S10.

One notes that the M13mp18 genomic DNA covers the complete sequence permutation space only for up to 4-base long sequence strings. Some of the 1024 possible 5-base long sequence strings already do not occur anymore (e.g ACTAG or AGAGC or CACGC). For 7-base long strings already most (more than 10,000) of the 16384 possible strings do not occur in the scaffold anymore. Therefore, the sequence space of 5- and longer sequence motifs is incomplete in the target DNA.

It appears very difficult to design a probe that can systematically sample incomplete sequence spaces for strings longer than 4 bases. Hence, we focussed on designing a probe that samples shorter sequence strings. To construct a minimal length probe sequence string we created a set of circular sequence strings that are de-Bruijn sequences of order 2, 3, and 4, respectively, for an alphabet of four letters. The strings were linearized into sets of oligonucleotides (Fig. S11). By design, it is certain that for example all of the 3-base long subsequence in the order-3 probe oligonucleotides have an exact match at least once in the scaffold sequence. In that sense, the de-Bruijn probe of order three is capable of sampling the entire scaffold DNA via 3-base motifs wherever it may be single-stranded. Sequence biases that arise through multiplicity of the search string or that are prescribed by the thermodynamic stability of different sequence motifs, such as AT vs GC rich situations, is unfortunately unavoidable.

To evaluate the degree of sequence bias arising from specific sequence binding to motifs of lengths greater than the order of the probe, we analyzed the number of exact sequence matches between de-Bruijn probe strings and the target DNA for strings with greater length. The result is shown in Supplementary Figure 12 Only the de-Bruijn probes of orders two and three have a low number of exact matches in the scaffold DNA sequence for longer sequence motifs (Fig. S12a, b). Long complementary sequence string matches as they can occur between target DNA and the probe of order 4, however, could increase undesirable specific sequence bias.

Using mfold [1], we also analyzed the de-Bruijn probes for secondary structure formation (Fig S13) and their tendency to form homo-dimers and heterodimers. All these effects could potentially compromise the utility of these DNA strands as defect probes since they compete with the desired binding to the target object. Due to the way the probe sequences are constructed, the set of sequences of the order-4 probe feature more GC-rich motifs and are thus systematically more prone to form sSupplementary Table econdary structures (Fig S13c). Regarding homo-/ and heterodimerization, the de-Bruijn probe of order two can form two homodimers with more than 5kcal/mol binding energy, the de-Bruijn probe of order three can form four homodimers with more than 5kcal/mol binding energy, and the de-Bruijn probe of order four can form a total of 31 different homodimeric structures with more than 5 kcal/mol binding energy. In addition, the de-Bruijn probe of order three can also form five heterodimers with at least 5 kcal/mol, and the de-Bruijn probe of order four can also form a total of 107 heterodimers with at least 5 kcal/mol.

Hence, as one might expect, the shortest probe has also the lowest propensity to react with itself, while the much longer de-Bruijn probe of order 4 that consists of six separate oligonucleotides forms many hairpins and can undergo multiple side reactions that might compromise its ability to sample all desired sequence motifs.

We tested the three de-Bruijn probes for their ability to label GC-rich versus AT-rich unpaired DNA defects in DNA origami structures. The test samples were prepared by omitting appropriately selected staple DNA strands from the assembly reactions. The expectation is that a “good” probe should faithfully label both types of defects, but give a greater signal for GC-rich defects due to the greater binding strength. Supplementary Figure 14 shows the result of these experiments.

One observation is that the absolute labeling intensity by the different de-Bruijn probes increases with the order of the probe (e.g compare Fig. S14 a vs. c vs. e). The brightest signals are provided by the de-Bruijn probe of order 4, while the weakest signals that can hardly be distinguished from background come from the probe of order 2. Most importantly, only the probes of order 2 and 3 exhibit the expected behaviour or labeling GC-rich defects more strongly than AT-rich defects (Fig. S14b and S14d). The probe of order 4, by contrast, labels AT-rich defects much more efficiently than GC-rich defects.

Together with the tendency of the de-Bruijn probe of order 4 to form many secondary structures and homo-/heterodimers we interpret this as an indication of unwanted sequence bias for probe 4. The signals provided by the probe of order 2 were too weak to be practically useful.

Hence, de-Bruijn probe of order 3 was the best choice for a defect probe and was thus analyzed in greater depth and used for our study.

References:

[1] M. Zuker. Mfold web server for nucleic acid folding and hybridization prediction. Nucleic Acids Res. 31 (13), 3406-3415, 2003.
